# Polyketides and Alkaloids from the Marine-Derived Fungus *Dichotomomyces cejpii* F31-1 and the Antiviral Activity of Scequinadoline A against Dengue Virus

**DOI:** 10.3390/md16070229

**Published:** 2018-07-06

**Authors:** Dong-Lan Wu, Hou-Jin Li, Duncan R. Smith, Janejira Jaratsittisin, Xia-Fu-Kai-Ti Xia-Ke-Er, Wen-Zhe Ma, Yong-Wei Guo, Jun Dong, Juan Shen, De-Po Yang, Wen-Jian Lan

**Affiliations:** 1 School of Pharmaceutical Sciences, Sun Yat-sen University, Guangzhou 510006, China; wudlan@mail2.sysu.edu.cn (D.-L.W.); xiakeer@mail2.sysu.edu.cn (X.-F.-K.-T.X.-K.-E.); guoyw8@mail2.sysu.edu.cn (Y.-W.G.); shenj23@mail2.sysu.edu.cn (J.S.); 2 School of Chemistry, Sun Yat-sen University, Guangzhou 510275, China; ceslhj@mail.sysu.edu.cn; 3 Institute of Molecular Bioscience, Mahidol University, Bangkok 10700, Thailand; duncan_r_smith@hotmail.com (D.R.S.); jajanejira@gmail.com (J.J.); 4 State Key Laboratory of Quality Research in Chinese Medicine, Macau Institute for Applied Research in Medicine and Health, Macau University of Science and Technology, Avenida Wai Long, Taipa 519020, Macau, China; wzma@must.edu.mo; 5 School of Traditional Chinese Medicine, Guangdong Pharmaceutical University, Guangzhou 510006, China; Dongjun@gdpu.edu.cn

**Keywords:** marine-derived fungus, *Dichotomomyces cejpii*, polyketides, alkaloids, antiviral activity, dengue virus

## Abstract

In our continuous chemical investigation on the marine-derived fungus *Dichotomomyces cejpii* F31-1, two new polyketides dichocetides B-C (**1**, **2**), two new alkaloids dichotomocejs E-F (**3**, **4**), and three known fumiquinozalines: scequinadoline A (**5**), quinadoline A (**6**), and scequinadoline E (**7**) were discovered from the culture broth and the mycelium in the culture medium, by the addition of l-tryptophan and l-phenylalanine. Their chemical structures were established by one dimensional (1D), two dimensional (2D) nuclear magnetic resonance (NMR) and high resolution mass spectrometry (HR-MS) data. Among them, scequinadoline A (**5**) exhibited significant inhibitory activity against dengue virus serotype 2 production by standard plaque assay, equivalent to the positive control andrographlide. Scequinadoline A (**5**) possesses the potential for further development as a dengue virus inhibitor.

## 1. Introduction

Dengue, the most prevalent mosquito-borne infection, is induced by four genetically similar, however, antigenically distinct serotypes of dengue virus (DENV)—DENV 1–4 [1,2]. DENV is rapidly spreading throughout the tropical and subtropical areas worldwide [3]. It is also an epidemic in provinces in Southern China, such as Guangdong, Fujian, and Jiangxi. Dengue has emerged as a severe public health problem and has imposed a heavy burden on both the infected individuals and the healthcare systems [4]. However, no licensed vaccines or specific antiviral drugs have been successfully developed for this disease [1]. The discovery and development of safe and effective antiviral drugs have therefore become imperative [3]. Natural products from marine microorganisms are chemically unique and exhibit a variety of interesting pharmaceutical properties [5]; indeed several compounds with anti-DENV activity have been isolated from the marine environment [6]. Among the compounds isolated from marine organisms, small metabolites of fungal origin represent a very important source for drug discovery [7,8]. Therefore, the search for small molecular dengue virus inhibitors from marine-derived fungi represents an attractive approach to treat the disease.

**Figure 1:**
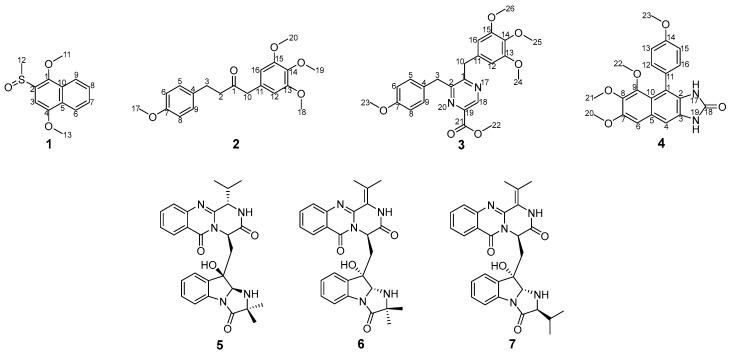
Chemical structures of compounds **1**–**7**.

The fungal strain *Dichotomomyces cejpii* F31-1 was isolated from the soft coral *Lobophytum crassum*. Previously, through the addition of amino acids, twenty-eight diverse compounds were obtained from the culture broth [9]. When searching for dengue virus inhibitors, a methanol extract of the mycelium displayed inhibitory activity against DENV 2. By bioactivity-guided isolation and purification, three known fumiquinozalines: scequinadoline A (**5**), quinadoline A (**6**), and scequinadoline E (**7**) have been obtained. Scequinadoline A (**5**) inhibited DENV 2 production by standard plaque assay, equivalent to the positive control andrographlide [10]. Furthermore, an additional two new polyketides dichocetides B-C (**1**, **2**) and two new alkaloids dichotomocejs E-F (**3**, **4**) were discovered from the culture broth (Figure 1).

## 2. Results and Discussion

### 2.1. Structural Elucidation

**Figure 2:**
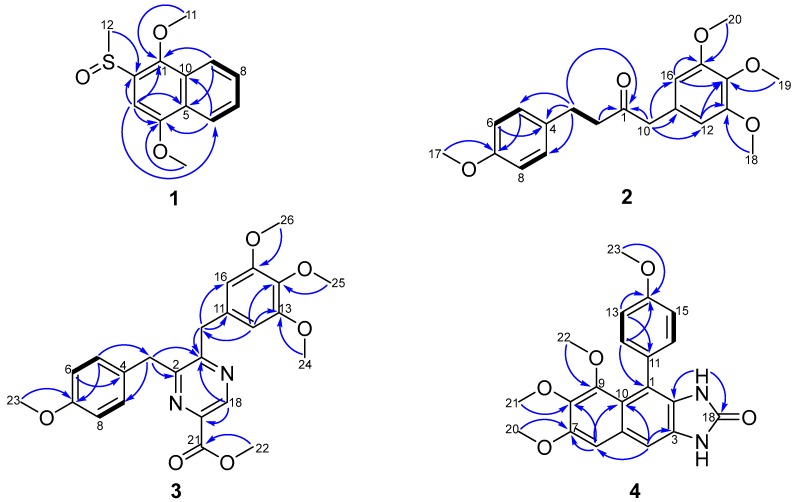
^1^H–^1^H COSY (bold line) and the key HMBC correlations (arrows) of compounds **1**–**4**.

Dichocetide B (**1**) was isolated as a yellow oil which resulted in an [M]^+^ ion peak by the high-resolution mass spectrometry (HR-ESI-MS) at *m*/*z* 251.0727 (calcd. for 251.0736), appropriate for a molecular formula of C_13_H_14_O_3_S, indicating seven sites of unsaturation. On the basis of 1D NMR spectra (Table 1 and Figures S3–S7), ten aromatic carbons were present (*δ*_C_ 96.5, 121.9, 123.2, 127.1, 127.6, 128.1, 128.2, 133.4, 145.3, 153.7). The skeletal structure for **1** was identified as a naphthalene ring in light of the distinct ^1^H–^1^H COSY cross-peaks of H-6 (*δ*_H_ 8.32, dd, 7.2, 1.6)/H-7 (*δ*_H_ 7.57, ddd, 7.2, 7.2, 1.2), H-7 (*δ*_H_ 7.57, ddd, 7.2, 7.2, 1.2)/H-8 (*δ*_H_ 7.61, ddd, 7.2, 7.2, 1.6), H-8 (*δ*_H_ 7.61, ddd, 7.2, 7.2, 1.6)/H-9 (*δ*_H_ 8.04, dd, 7.2, 1.2), and the heteronuclear multiplebond correlation spectroscopy (HMBC) correlations from H-3 (*δ*_H_ 7.25, s) to C-1 (*δ*_C_ 145.3), C-2 (*δ*_C_ 133.4), C-4 (*δ*_C_ 153.7), C-5 (*δ*_C_ 128.1) and C-6 (*δ*_C_ 123.2), from H-6 (*δ*_H_ 8.32, dd, 7.2, 1.6) to C-4 (*δ*_C_ 153.7) and C-10 (*δ*_C_ 128.2), from H-9 (*δ*_H_ 8.04, dd, 7.2, 1.2) to C-1 (*δ*_C_ 145.3) and C-5 (*δ*_C_ 128.1). Eventually, the HMBC correlations of H-11 (*δ*_H_ 3.98, s) to C-1 (*δ*_C_ 145.3), from H-12 (*δ*_H_ 2.84, s) to C-2 (*δ*_C_ 133.4) and from H-13 (*δ*_H_ 4.08, s) to C-4 (*δ*_C_ 153.7) revealed that C-2 was bound to a sulfur methyl, and C-1 and C-4 were connected to a methoxyl group respectively (Figure 2). Therefore, the chemical structure of **1** was elucidated as shown in Figure 1.

Dichocetide C (**2**) was obtained as a yellow oil. Its molecular formula was established to be C_20_H_24_O_5_, owing to the presence of an [M + Na]^+^ ion peak in the HR-ESI-MS at *m*/*z* 367.1504 (calcd. for 367.1516), indicating nine degrees of unsaturation. The ^13^C NMR and distortionless enhancement by polarization transfer (DEPT135) spectra (Table 2 and Figures S15 and S16) showed the presence of six sp^2^ quaternary carbons [*δ*_C_ 129.8, 133.0, 137.1, 153.5 (2C), 158.1] and six sp^2^ methines [*δ*_C_ 106.5 (2C), 114.0 (2C), 129.4 (2C)]. The ^1^H–^1^H COSY cross-peak of H-5 (H-9) (*δ*_H_ 7.05, d, 8.4, 2H)/H-6 (H-8) (*δ*_H_ 6.79, d, 8.4, 2H) and the HMBC correlations from H-5 (H-9) (*δ*_H_ 7.05, d, 8.4, 2H) to C-7 (*δ*_C_ 158.1), from H-6 (H-8) (*δ*_H_ 6.79, d, 8.4, 2H) to C-4 (*δ*_C_ 133.0) indicated the existence of a para-substituted aromatic ring. A further aromatic ring was identified to be 3,4,5-trimethoxyphenyl groups, on the grounds of the key HMBC correlations from H-12 (H-16) (*δ*_H_ 6.34, s) to C-14 (*δ*_C_ 137.1)/C-13 (C-15) (*δ*_C_ 153.5) and from H-10 (*δ*_H_ 3.57, s) to C-11 (*δ*_C_ 129.8)/C-12 (C-16) (*δ*_C_ 106.5). The HMBC correlations from H-10 (*δ*_H_ 3.57, s) and H-2 (*δ*_H_ 2.76, dd, 14.8, 8.0) to C-1 (*δ*_C_ 207.7), and the ^1^H–^1^H COSY cross-peak of H-2 (*δ*_H_ 2.76, 14.8, 8.0)/H-3 (*δ*_H_ 2.82, dd, 14.0, 7.2), demonstrated the presence of a –CH_2_–CH_2_–CO–CH_2_– moiety. The HMBC correlations from H-3 (*δ*_H_ 2.82, dd, 14.0, 7.2) to C-4 (*δ*_C_ 133.0)/C-5 (C-9) (*δ*_C_ 129.4) and from H-10 (*δ*_H_ 3.57, s) to C-11 (*δ*_C_ 129.8)/C-12 (C-16) (*δ*_C_ 106.5) proved that the –CH_2_–CH_2_–CO–CH_2_– moiety formed the connecting bridge between the para-substituted and 1,3,4,5-tetrasubstituted benzene ring, as shown in Figure 1. Furthermore, the HMBC correlations of H-17 (*δ*_H_ 3.77, s) to C-7 (*δ*_C_ 158.1), of H-18 (H-20) (*δ*_H_ 3.82, s) to C-13 (C-15) (*δ*_C_ 153.5) and H-19 (*δ*_H_ 3.83, s) to C-14 (*δ*_C_ 137.1) displayed that C-7, C-13 (C-15) and C-14 were each connected to a methoxyl group (Figure 2). Consequently, the chemical structure of 2 was constructed as shown in Figure 1.

Dichotomocej E (**3**) was isolated as a yellow oil. The molecular formula was determined as C_24_H_26_N_2_O_5_ in accordance with the HR-ESI-MS protonated molecular ion detected at *m*/*z* 439.1880 [M + H]^+^ (calcd. for 439.1884), indicating nine indices of hydrogen deficiency. According to the 1D NMR spectra (Table 2 and Figures S22 and S23), there were seventeen aromatic carbons present [*δ*_C_ 106.1 (2C), 114.3 (2C), 129.5, 129.8 (2C), 132.9, 137.0, 140.6, 143.7, 153.4 (2C), 154.8, 158.6 (2C), 165.1]. The same carbon skeleton as 2, in which the –CH_2_–CH_2_–CO–CH_2_– moiety formed the bridge connecting the para-substituted and 1,3,4,5-tetrasubstituted benzene ring, was confirmed by comparing the spectra (Table 2 and Figures S14–S19, S22–S30 and S32–S37) of the two compounds. The difference between 3 and 2 was a pyrazine in 3 taking the place of the –CH_2_–CO– fragment in 2; concluded due to the presence of chemical shifts typical of pyrazine (*δ*_C_ 140.6, 143.7) and the key HMBC cross-peaks from H-3 (*δ*_H_ 4.26, s), H-10 (*δ*_H_ 4.10, s) and H-18 (*δ*_H_ 9.15, s) to C-1 (*δ*_C_ 158.6), from H-3 (*δ*_H_ 4.26, s) to C-2 (*δ*_C_ 154.8) and from H-18 (*δ*_H_ 9.15, s) to C-1 (*δ*_C_ 158.6). The key HMBC from H-18 (*δ*_H_ 9.15, s) to C-1 (*δ*_C_ 158.6)/C-19 (*δ*_C_ 140.6), from H-22 (*δ*_H_ 4.04, s) to C-21 (*δ*_C_ 165.1) suggested a methyl acetate attached to C-19 and two methylenes attached to C-1 and C-2 respectively (Figure 2). As a result, the chemical structure of 3 was illustrated as shown in Figure 1.

Dichotomocej F (**4**) was isolated as a yellow powder with a molecular formula of C_21_H_20_N_2_O_5_ based on the negative HR-ESI-MS [M − H]^−^ peak at *m*/*z* 379.1299 (calcd. for 379.1299), implying thirteen degrees of unsaturation. Inspection of the 1D NMR spectra recorded for 4 in CDCl_3_ (Table 2 and Figures S40–S43) indicated resonances that could be assigned to seventeen sp^2^ carbons and four methoxy groups. Careful analysis of the 1D and 2D NMR spectra of 4 displayed that an AA′BB′ spin system of a para-substituted aromatic ring was present from the key ^1^H–^1^H COSY cross-peak of H-12 (H-16) (*δ*_H_ 7.29, d, 8.4 2H)/H-13 (H-15) (*δ*_H_ 7.00, d, 8.4, 2H) and the HMBC correlations observed between C-11 (*δ*_C_ 130.4) and H-13 (*δ*_H_ 7.00, d, 8.4, 2H), between C-14 (*δ*_C_ 158.8) and H-12 (*δ*_H_ 7.29, d, 8.4 2H), H-13 (*δ*_H_ 7.00, d, 8.4, 2H) and H-23 (*δ*_H_ 3.88, s). In addition to the four degrees of unsaturation, a further seven sites of unsaturation are attributed to a naphthalene ring built on the HMBC spectrum from H-4 (*δ*_H_ 7.24, s) to C-3 (*δ*_C_ 128.4), C-6 (*δ*_C_ 102.9) and C-10 (*δ*_C_ 119.1), from H-6 (*δ*_H_ 6.94, s) to C-7 (*δ*_C_ 151.7), C-8 (*δ*_C_ 141.2) and C-10 (*δ*_C_ 119.1). The HMBC correlations of H-20 (*δ*_H_ 3.96, s) to C-7 (*δ*_C_ 151.7), from H-21 (*δ*_H_ 3.88, s) to C-8 (*δ*_C_ 141.2) and H-22 (*δ*_H_ 3.28, s) to C-9 (*δ*_C_ 149.8) showed that three methoxyl groups were attached to C-7, C-8 and C-9 separately. The existence of an imidazolidin was verified by the chemical signals of two active protons [(*δ*_H_ 7.34, brs), (*δ*_H_ 8.58, brs)] and the pivotal HMBC correlations from NH-17 (*δ*_H_ 7.34, brs) to C-2 (*δ*_C_ 128.7) and C-18 (*δ*_C_ 155.9), matching with the remaining two sites of unsaturation (Figure 2). The strict comparison of the NMR spectra with a synthetic compound named 1,3-Dihydro-5,6,7-trimethoxy-4-(4-methoxyphenyl)-1-methyl-2*H*-naphth[2,3-*d*]imidazole-2-one highlighted that the only subtle difference between the known compound and 4 was a methoxyl group in N-19 being replaced by a hydrogen in 4 [11]. Therefore, the chemical structure of 4 was elucidated as shown in Figure 1.

In accordance with the comparison of the spectroscopic data of compounds **5**–**7** (Figures S48–S53) with those reported in the literature previously, the chemical structures were interpreted as scequinadoline A (**5**) [12], quinadoline A (**6**) [13], and scequinadoline E (**7**) [12].

### 2.2. Biological Activity

#### 2.2.1. Cytotoxicity of Scequinadoline A (**5**), Quinadoline A (**6**), and Scequinadoline E (**7**)

Cytotoxicity of the compounds was initially determined by observation of alterations in cell morphology. HEK293T/17 cells were incubated with various concentrations of scequinadoline A (**5**), quinadoline A (**6**), and scequinadoline E (**7**) (1, 10 and 50 μM) for 24 h, in parallel with andrographolide (1, 10, 50 and 100 μM) treated cells as a positive control, in addition to a dimethyl sulfoxide (DMSO) control. No significant cytotoxicity was observed for all concentrations of scequinadoline A (**5**), quinadoline A (**6**), and scequinadoline E (**7**) 24 h post treatment, compared to the controls (Figure S1).

#### 2.2.2. Optimization of DENV 2 Infection

**Figure 3:**
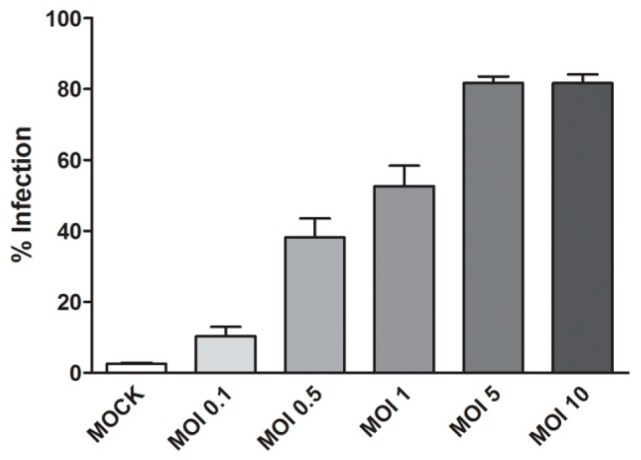
HEK293T/17 cells were infected with DENV 2 at MOIs of 0.1, 0.5, 1, 5, 10 for 2 h. At 24 h post infection (p.i.) the cells were collected and the percentage of infection determined by flow cytometry. All experiments were performed independently in triplicate. Error bars show standard deviation (SD).

The percentage of DENV 2 infection was optimized by varying the multiplicity of infection. HEK293T/17 cells were infected with DENV 2 at multiplicity of infections (MOI) of 0.1, 0.5, 1, 5, 10 for 2 h. The infected cells were collected at 24 h post infection (p.i.) and the level of infection determined by flow cytometry. The levels of infection increased with increasing MOI. Based on the results (Figure 3), a MOI of 5 was selected.

#### 2.2.3. Effect of Compounds **5**–**7** on DENV Infection

**Figure 4:**
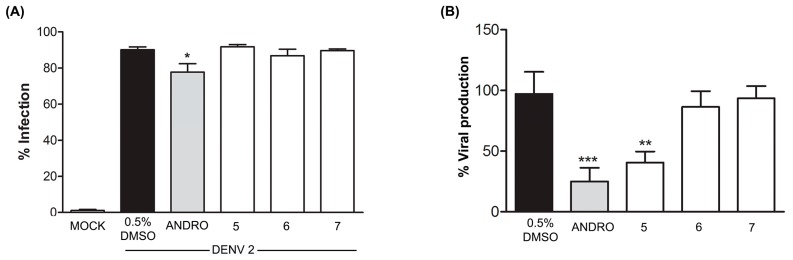
DENV2 infected HEK293T/17 cells were treated with 50 μM of scequinadoline A (**5**), quinadoline A (**6**) and scequinadoline E (**7**), in parallel with 50 μM of andrographlide and DMSO as a control. After 24 h of incubation, the treated cells were collected for evaluation of the percentage of infection by flow cytometry (**A**) and the supernatant was collected to determine the viral production by plaque assay (**B**). All experiments were undertaken as independent triplicates. Data is shown normalized against the control (0.5% DMSO). Error bars represent SD (* *p* < 0.05, ** *p* < 0.01, *** *p* < 0.001).

The levels of DENV 2 infection in cells treated with compounds **5**–**7** were not significantly different from control (DMSO) treated cells, while cells treated with andrographolide showed a significant reduction in DENV 2 infection levels, in agreement with our earlier observation [10]. While quinadoline A (**6**) and scequinadoline E (**7**) showed no effect on DENV virus production as assessed by standard plaque assay, a significant reduction in virus output was observed upon treatment with scequinadoline A (**5**) as compared to control (DMSO) treated cells (Figure 4).

## 3. Materials and Methods

### 3.1. General Procedures

The Shimadzu UV-Vis-NIR spectrophotometer (Shimadzu Corporation, Nakagyo-ku, Kyoto, Japan) was used for UV spectra analysis. The Fourier transform infrared (FT-IR) spectrophotometer (PerkinElmer Frontier, Waltham, MA, USA) with an Ever-Glomid/near-IR source was applied for IR spectra measurement. 1D and 2D NMR spectra were recorded on the Bruker Avance II 400 and 500 spectrometers (Bruker Bio Spin AG, Industriestrasse 26, Fällanden, Switzerland) in CDCl_3,_ and the chemical shifts are corresponding to the residual solvent signals (CDCl_3_: δ_H_ 7.260 and δ_C_ 77.160). The low and high resolution ESI-MS spectra were obtained with Thermo LCQ DECA XP (San Diego, CA, USA) liquid chromatography-mass spectrometry and performed on Thermo Fisher LTQ Orbitrap Elite High Resolution liquid chromatography-mass spectrometry (Thermo Fisher Scientific Inc., Waltham, MA, USA) severally. Preparative high performance liquid chromatography (HPLC) was performed on a Shimadzu LC-20AT HPLC pump (Shimadzu Corporation, Nakagyo-ku, Kyoto, Japan) outfitted with an SPD-20A dual *λ* absorbance detector (Shimadzu Corporation, Nakagyo-ku, Kyoto, Japan), and a Shim-pack PRC-ODS HPLC column I (250 × 20 mm i.d., 5 μm, Shimadzu Corporation, Nakagyo-ku, Kyoto, Japan). Silica gel (SiO_2_, 200–300 mesh, Qingdao Marine Chemical Inc., Qingdao, China) and Sephadex LH-20 (green herbs, Beijing, China) were used for column chromatography.

### 3.2. Fungal Material

*Dichotomomyces cejpii* F31-1, the marine fungus, was isolated from the inner tissue of the soft coral *Lobophytum crassum* which was acquired from Hainan Sanya National Coral Reef Reserve, P. R. China. The fungal strain was preserved in 15.0% (*v*/*v*) glycerol aqueous solution at −80 °C. A voucher specimen was stored in the School of Pharmaceutical Sciences, Sun Yat-sen University, Guangzhou, P. R. China. The ITS rDNA (GenBank EF669956) analysis by BLAST database screening furnished a hundred percent match to *Dichotomomyces cejpii*. 

### 3.3. Culture, Extraction, and Isolation

Fungus strain *Dichotomomyces cejpii* F31-1 was cultured in the medium (yeast extract 2 g/L, sea salt 30 g/L, glucose 20 g/L, peptone 5 g/L, L-Phe 2 g/L, D, L-Trp 2 g/L, H_2_O 1 L, pH 7.5). Fungal mycelia were cut uniformly and transferred aseptically to 1 L Erlenmeyer flasks with each containing 600 mL sterilized liquid medium. The flasks were incubated for 60 days at 25 °C. Cheesecloth was used for filtration of 90 liters of liquid culture. The culture was extracted three times by using EtOAc and then was concentrated under reduced pressure to provide crude extract (35 g) when the mycelium was extracted three times by using MeOH and then was concentrated under reduced pressure to furnish a crude extract (15 g). 

A silica gel column (diameter: 8 cm, length: 30 cm, silica gel: 125 g) with a gradient of petroleum ether-EtOAc (10:0–0:10, *v*/*v*) to EtOAc-MeOH (10:0–0:10, *v*/*v*) was carried on the extract of mycelium (15 g) to yield eleven fractions (Fr. 1–Fr. 7). Compound **6** (4.5 mg) was crystallized from Fr. 7, and compound **5** (3.3 mg) and compound **7** (3.0 mg) were gained from fractions (Fr. 7) by a Sephadex LH-20 column and eluted with MeOH.

Compound **1** (1.3 mg) and compound **2** (2.0 mg) were acquired from the extract of culture (35 g) by silica gel column (diameter: 8 cm, length: 80 cm, silica gel: 300 g) with a gradient of petroleum ether-EtOAc-MeOH (10:0:0–0:0:10, *v*/*v*) and the fractions were further purified with Sephadex LH-20 and preparative HPLC (MeOH-H_2_O, 45:55, *v*/*v*, column I). Compound **3** (1.6 mg) was isolated by column chromatography over ODS (MeOH-H_2_O, 7:3–1:9, *v*/*v*) and preparative HPLC (MeOH-H_2_O, 65:35, *v*/*v*, column I). Compound **4** (1.1 mg) was obtained by chromatographing on SiO_2_ by a gradient solvent system of petroleum ether-EtOAc (10:1–0:10, *v*/*v*, 100–200 mesh) and using Sephadex LH-20 and preparative HPLC (MeOH-H_2_O, 65:35, *v*/*v*, column I) of further purification.

Dichocetide B (**1**): Yellow oil, UV (MeOH) *λ*_max_ (log *ε*): 201 (4.02), 215 (4.20), 248 (4.03), 304 (3.45), 318 (3.39), 333 (3.31) nm; IR (KBr) ν_max_ 1658, 1583, 1540, 1446 cm^−1^. ^1^H and ^13^C NMR data, shown in Table 1; [M]^+^ in the HR-ESI-MS *m*/*z* 251.0728 (calcd. for C_13_H_14_O_3_S, 251.0736).

Dichocetide C (**2**): Yellow oil, UV (MeOH) *λ*_max_ (log *ε*): 2.03 (4.52), 226 (4.22), 277 (3.67) nm; IR (KBr) *ν*_max_ 1713, 1590, 1529, 1511, 1462, 1421 cm^−1^. ^1^H and ^13^C NMR data, shown in Table 2; [M + Na]^+^ in the HR-ESI-MS *m*/*z* 367.1504 (calcd. for C_24_H_26_N_2_O_5_, 367.1516).

Dichotomocej E (**3**): Yellow oil, UV (MeOH) *λ*_max_ (log *ε*): 203 (4.87), 224 (4.62), 277 (4.26) nm; IR (KBr) ν_max_ 3252, 2995, 2932, 2835, 1723, 1588, 1508, 1457, 1422, 1243, 808, 777 cm^−1^. ^1^H and ^13^C NMR data, shown in Table 2; [M + H]^+^ in the HR-ESI-MS *m*/*z* 439.1884 (calcd. for C_20_H_24_O_5_, 439.1864).

Dichotomocej F (**4**): Yellow powder, UV (MeOH) *λ*_max_ (log *ε*): 201 (4.64), 250 (4.68), 308 (3.94), 340 (3.83) nm; IR (KBr) ν_max_ 3004, 2926, 2854, 1707, 1608, 1460, 1422, 1244, 1102, 1006, 861, 754, 672 cm^−1^. ^1^H and ^13^C NMR data, shown in Table 2; [M − H]^−^ in the HR-ESI-MS *m*/*z* 379.1299 (calcd. for C_21_H_20_N_2_O_5_, 379.1299).

### 3.4. Cell line and DENV2

Human embryonic kidney HEK293T/17 cells were cultured as described previously [10] in Dulbecco’s Modified Eagle Medium (DMEM; Gibco, Invitrogen, Carlsbad, CA, USA) with 10% heat-inactivated fetal bovine serum (FBS, Gibco, Invitrogen) and 100 units/mL of penicillin/streptomycin (Gibco, Invitrogen), and incubated at 37 °C with 5% CO_2_. DENV 2 (strain 16,681) was propagated in Aedes albopictus C6/36 cells. Supernatant containing virus progeny was harvested, supplemented with 20% heat-inactivated FBS and stored at −80 °C. DENV 2 viral titer was determined by standard plaque assay on LLC-MK2 (Rhesus monkey kidney) cells essentially as described previously [14].

### 3.5. Compounds Preparation

Scequinadoline A (**5**), quinadoline A (**6**) and scequinadoline E (**7**) were dissolved in 100% DMSO to a final concentration of 10 mM, and stored at −80 °C. Compounds were diluted with complete DMEM. Andrographolide (365645, Sigma, St. Louis, MO, USA) was used as a positive control for compound screening. Final concentration of andrographolide at 10 mM was prepared by dissolving with 100% DMSO. Control of experiments contained 0.5% DMSO.

### 3.6. Cytotoxicity Assay

Cytotoxicity of the compounds was initially determined by observation of alterations of cell morphology. HEK293T/17 cells were seeded on six well plates under standard growth condition. Cells were incubated with 1, 10 and 50 μM of scequinadoline A (**5**), quinadoline A (**6**) and scequinadoline E (**7**). At 24 h post treatment, cell morphology was directly observed under inverted light microscope (Nikon EclipseTS100, Nikon Instruments Inc., Melville, NY, USA). The treated cells were compared with individual DMSO concentrations and various concentrations of andrographolide were used as a positive control.

### 3.7. Antiviral Activity against DENV 2

HEK293T/17 cells were cultured in six well plates until they reached approximately 90% confluency. Cells were mock infected or infected with DENV 2 at 5 p.f.u/mL at 37 °C with 5% CO_2_ for 2 h. Then virus was removed and the 50 μM of scequinadoline A (**5**), quinadoline A (**6**), scequinadoline E (**7**) and andrographolide added to the cells at 0 h after infection, together with DMSO control. For further experiments, supernatant and cells were collected at 24 h post treatment.

Mock and DENV 2 infected cells from compound treatments were collected at 24 h.p.i and incubated with 100 μL of 10% normal goat serum (NGS; Gibco^TM^ Invitrogen) in PBS/IFA on ice for 30 min. Then, cells were washed with 800 μL of 1% BSA in PBS/IFA followed by fixing with 200 μL of 4% paraformaldehyde in PBS/IFA in dark at room temperature for 20 min. After washing cells twice with 1% BSA in PBS/IFA, cells were permeabilized with 200 μL of 0.2% Triton X in PBS/IFA at room temperature in dark for 10 min. The cell were washed twice with 1% BSA in PBS/IFA and incubated with 50 μL of a mouse monoclonal anti-DENV E protein antibody (HB114) with dilution 1:150 at 4 °C for overnight. Subsequently, cells were washed twice with 1% BSA in PBS/IFA, followed by incubation with 50 μL goat anti-mouse IgG antibody conjugated with FITC (Milipore Corp., Burlington, MA, USA) at room temperature for 1 h in dark at room temperature. Finally, cells were washed twice with 1% BSA in PBS/IFA and analyzed by flow cytometry on BD FACSCalibur cytometer (Becton Dickinson, San Jose, CA, USA) using CELLQuest™ software. All experiments were performed as independent triplicates.

All data were analyzed using GraphPad Prism program (GraphPad Software). Statistical analysis was performed using the PASW statistics 18 (SPSS Inc., Chicago, IL, USA). The percentage of infection and viral production from triplicate value of independent experiments are shown as mean ± S.D. A *p* value less than 0.05 was considered as statistically significant.

## 4. Conclusions

Overall, an amino acid strategy is effective for eliciting the fungus *Dichotomomyces cejpii* F31-1 to produce alkaloids with chemical diversity. Among the thirty-five compounds produced by the fungus in total, twenty-six compounds are alkaloids. The fungus showed amazing potential for the biosynthesis of alkaloids and other types of natural products. Additionally, scequinadoline A (**5**) exhibited a significant inhibitory activity against DENV 2 production as assessed by standard plaque assay. The inhibition of virus production but not infection level suggests that this compound inhibits virus formation or virus release from the cell. Scequinadoline A (**5**) displays the potential for development as a dengue virus inhibitor. Dichocetide C (**2**) and dichotomocejs E-F (**3**, **4**) were assayed for cytotoxic activity against a macrophage cell line (RAW264.7) and displayed no significant inhibitory effect.

## Figures and Tables

**Table 1:** ^1^H (400 Hz) and ^13^C (100 Hz) NMR data for compound **1** in CDCl_3_.

No.		1
	*δ*_C_, Type	*δ*_H_, Mult. (*J*, Hz)
1	145.3, C	
2	133.4, C	
3	96.5, CH	7.25, s
4	153.7, C	
5	128.1, C	
6	123.2, CH	8.32, dd (7.2, 1.6)
7	127.1, CH	7.57, ddd (7.2, 7.2, 1.2)
8	127.6, CH	7.61, ddd (7.2, 7.2, 1.6)
9	121.9, CH	8.04, dd (7.2, 1.2)
10	128.2, C	
11	63.0, OCH_3_	3.98, s
12	42.5, SOCH_3_	2.84, s
13	56.3, OCH_3_	4.08, s

**Table 2:** ^1^H (400 Hz) and ^13^C (100 Hz) NMR data for compounds **2**–**4** in CDCl_3_.

No.		2		3		4
	*δ*_C_, Type	*δ*_H_, Mult. (*J*, Hz)	*δ*_C_, Type	*δ*_H_, Mult. (*J*, Hz)	*δ*_C_, Type	*δ*_H_, Mult. (*J*, Hz)
1	207.7, CO		158.6, C		116.6, C	
2	43.7, CH_2_	2.76, dd (14.8, 8.0)	154.8, C		128.7, C	
3	29.1, CH_2_	2.82, dd (14.0, 7.2)	40.6, CH_2_	4.26, s	128.4, C	
4	133.0, C		129.5, C		104.3, CH	7.24, s
5	129.4, CH	7.05, d (8.4)	129.8, CH	7.04, d (8,4)	128.4, C	
6	114.0, CH	6.79, d (8.4)	114.3, CH	6.80, d (8.4)	102.9, CH	6.94, s
7	158.1, C		158.6, C		151.7, C	
8	114.0, CH	6.79, d (8.4)	114.3, CH	6.80, d (8.4)	141.2, C	
9	129.4, CH	7.05, d (8.4)	129.8, CH	7.04, d (8,4)	149.8, C	
10	50.8, CH_2_	3.57, s	41.2, CH_2_	4.10, s	119.1, C	
11	129.8, C		132.9, C		130.4, C	
12	106.5, CH	6.34, s	106.1, CH	6.21, s	130.3, CH	7.29, d (8.4)
13	153.5, C		153.4, C		113.6, CH	7.00, d (8.4)
14	137.1, C		137.0, C		158.8, CH	7.24, s
15	153.5, C		153.4, C		113.6, CH	7.00, d (8.4)
16	106.5, CH	6.34, s	106.1, CH	6.22, s	130.3, CH	7.29, d (8.4)
17	55.4, CH_3_	3.77, s			NH	7.34, brs
18	56.2, CH_3_	3.82, s	143.7, CH	9.15, s	155.9, CO	
19	61.0, CH_3_	3.83, s	140.6, C		NH	8.58, brs
20	56.2, CH_3_	3.82, s			55.9, OCH_3_	3.96, s
21			165.1, CO		61.2, OCH_3_	3.88, s
22			53.2, OCH_3_	4.04, s	60.7, OCH_3_ 55.5, OCH_3_	3.28, s 3.88, s
23			55.4, OCH_3_	3.73, s		
24			56.2, OCH_3_	3.74, s		
25			61.0, OCH_3_	3.80, s		
26			56.2, OCH_3_	3.73, s		

## Supplementary Materials

The evaluation of cytotoxicity of **5**–**7** and the HR-ESI-MS and NMR spectra of compounds **1**–**7** (Figures S1–S53) are available online.
