# Supplementary material for: Polyketides and Alkaloids from the Marine-Derived Fungus Dichotomomyces cejpii F31-1 and the Antiviral Activity of Scequinadoline A against Dengue Virus
Source: Mar Drugs. 2018 Jul 6;16(7):229. doi: 10.3390/md16070229 (PMC6071211; doi:10.3390/md16070229)

Supporting Information

Polyketides and Alkaloids from the Marine-Derived Fungus *Dichotomomyces cejpii* F31-1 and the Antiviral Activity of Scequinadoline A against Dengue Virus

Dong-Lan Wu [1], Hou-Jin Li [2], Duncan R. Smith [3], Janejira Jaratsittisin [3], Xia-Fu-Kai-Ti Xia-Ke-Er [1], Wen-Zhe Ma [4], Yong-Wei Guo [1], Jun Dong [5], Juan Shen [1], De-Po Yang [1,*], Wen-Jian Lan [1,*]

[1] School of Pharmaceutical Sciences, Sun Yat-sen University, Guangzhou 510006, China; wudlan@mail2.sysu.edu.cn (D.W.); xiakeer@mail2.sysu.edu.cn (X.X.); guoyw8@mail2.sysu.edu.cn (Y.G.); shenj23@mail2.sysu.edu.cn (J.S.)
[2] School of Chemistry, Sun Yat-sen University, Guangzhou 510275, China; ceslhj@mail.sysu.edu.cn (H.L.)
[3] Institute of Molecular Bioscience, Mahidol University, Bangkok 10700, Thailand; duncan_r_smith@hotmail.com (D.S.); jajanejira@gmail.com (J.J.)
[4] State Key Laboratory of Quality Research in Chinese Medicine, Macau Institute for Applied Research in Medicine and Health, Macau University of Science and Technology, Avenida Wai Long, Taipa 519020, Macau (SAR), China; wzma@must.edu.mo (W.M.)
[5] School of Traditional Chinese Medicine, Guangdong Pharmaceutical University, Guangzhou 510006, China; Dongjun@gdpu.edu.cn
[*] Correspondence: lssydp@mail.sysu.edu.cn (D.Y.); lanwj@mail.sysu.edu.cn (W.L.); Tel.: +86-020-3994-3043 (D.Y.); Tel.: +86-020-3994-3042 (W.L.)

List of Contents

Figure S1. The evaluation of cytotoxicity of scequinadolines A (**5**), quinadoline A (**6**) and scequinadolines E (**7**)........S5

Figure S2. HR-ESI-MS spectrum of dichocetide B (**1**)........S6

Figure S3. ^{1}H NMR spectrum of dichocetide B (**1**) in $CDCl_3$ (400MHz)........S7

Figure S4. An expansion of ^{1}H NMR spectrum of dichocetide B (**1**) in $CDCl_3$ (400MHz)........S8

Figure S5. ^{13}C NMR spectrum of dichocetide B (**1**) in CDCl3 (100MHz)........S9

Figure S6. An expansion of ^{13}C NMR spectrum of dichocetide B (**1**) in $CDCl_3$ (100MHz)........S10

Figure S7. DEPT 135 spectrum of dichocetide B (**1**) in $CDCl_3$ (100MHz)........S11

Figure S8. HMQC spectrum of dichocetide B (**1**) in $CDCl_3$........S12

Figure S9. ^{1}H-^{1}H COSY spectrum of dichocetide B (**1**) in $CDCl_3$........S13

Figure S10. HMBC spectrum of dichocetide B (**1**) in $CDCl_3$........S14

Figure S11. An expansion of HMBC spectrum of dichocetide B (**1**) in $CDCl_3$........S15

Figure S12. NOESY spectrum of dichocetide B (**1**) in $CDCl_3$........S16

Figure S13. HR-ESI-MS spectrum of dichocetide C (**2**)........S17

Figure S14. ^{1}H NMR spectrum of dichocetide C (**2**) in $CDCl_3$ (400MHz)........S18

Figure S15. ^{13}C NMR spectrum of dichocetide C (**2**) in $CDCl_3$ (100MHz)........S19

Figure S16. DEPT 135 spectum of dichocetide C (**2**) in $CDCl_3$ (100MHz)........S20

Figure S17. HMQC spectrum of dichocetide C (**2**) in $CDCl_3$........S21

Figure S18. ^{1}H-^{1}H COSY spectrum of dichocetide C (**2**) in $CDCl_3$........S22

Figure S19. HMBC spectrum of dichocetide C (**2**) in $CDCl_3$........S23

Figure S20. NOESY spectrum of dichocetide C (**2**) in $CDCl_3$........S24

Figure S21. HR-ESI-MS spectrum of dichotomocej E (**3**)........S25

Figure S22. ^{1}H NMR spectrum of dichotomocej E (**3**) in $CDCl_3$ (400MHz)........S26

Figure S23. ^{13}C NMR spectrum of dichotomocej E (**3**) in $CDCl_3$ (100MHz)............................S27

Figure S24. ^{1}H NMR spectrum of dichotomocej E (**3**) in $CDCl_3$ (400MHz)............................S28

Figure S25. ^{13}C NMR spectrum of dichotomocej E (**3**) in $CDCl_3$ (100MHz)............................S29

Figure S26. An expansion of ^{13}C NMR spectrum of dichotomocej E (**3**) in $CDCl_3$ (100MHz)............................S30

Figure S27. DEPT 135 spectrum of dichotomocej E (**3**) in $CDCl_3$ (100MHz)............................S31

Figure S28. HMQC spectrum of dichotomocej E (**3**) in $CDCl_3$............................S32

Figure S29. ^{1}H-^{1}H COSY spectrum of dichotomocej E (**3**) in $CDCl_3$............................S33

Figure S30. HMBC spectrum of dichotomocej E (**3**) in $CDCl_3$............................S34

Figure S31. NOESY spectrum of dichotomocej E (**3**) in $CDCl_3$............................S35

Figure S32. ^{1}H NMR spectrum of dichotomocej E (**3**) in $CDCl_3$ (500MHz)............................S36

Figure S33. ^{13}C NMR spectrum of dichotomocej E (**3**) in $CDCl_3$ (125Hz)............................S37

Figure S34. DEPT 135 spectrum of dichotomocej E (**3**) in $CDCl_3$ (125MHz)............................S38

Figure S35. HMQC spectrum of dichotomocej E (**3**) in $CDCl_3$............................S39

Figure S36. ^{1}H-^{1}H COSY spectrum of dichotomocej E (**3**) in $CDCl_3$............................S40

Figure S37. HMBC spectrum of dichotomocej E (**3**) in $CDCl_3$............................S41

Figure S38. NOESY spectrum of dichotomocej E (**3**) in $CDCl_3$............................S42

Figure S39. HR-ESI-MS spectrum of dichotomocej F (**4**)............................S43

Figure S40. ^{1}H NMR spectrum of dichotomocej F (**4**) in $CDCl_3$ (400MHz)............................S44

Figure S41. An expansion of ^{1}H NMR spectrum of dichotomocej F (**4**) in $CDCl_3$ (400MHz)............................S45

Figure S42. ^{13}C NMR spectrum of dichotomocej F (**4**) in $CDCl_3$ (100MHz)............................S46

Figure S43 DEPT 135 spectum of dichotomocej F (**4**) in $CDCl_3$ (100MHz)............................S47

Figure S44. HMQC spectrum of dichotomocej F (**4**) in $CDCl_3$............................S48

Figure S45. ^{1}H-^{1}H COSY spectrum of dichotomocej F (**4**) in $CDCl_3$............................S49

Figure S46. HMBC spectrum of dichotomocej F (**4**) in $CDCl_3$............................S50

Figure S47. NOESY spectrum of dichotomocej F (**4**) in $CDCl_3$............................S51

Figure S48. ^{1}H NMR spectrum of scequinadolines A (**5**) in $CDCl_3$ (400MHz)............................S52

Figure S49. ^{13}C NMR spectrum of scequinadolines A (**5**) in $CDCl_3$ (100MHz)..............................S53

Figure S50. ^{1}H NMR spectrum of quinadoline A (**6**) in $CDCl_3$ (400MHz)……......................S54

Figure S51. ^{13}C NMR spectrum of quinadoline A (**6**) in $CDCl_3$ (100MHz)..............................S55

Figure S52. ^{1}H NMR spectrum of scequinadolines E (**7**) in $CDCl_3$ (400MHz)……......................S56

Figure S53. ^{13}C NMR spectrum of scequinadolines E (**7**) in $CDCl_3$ (100MHz)..............................S57

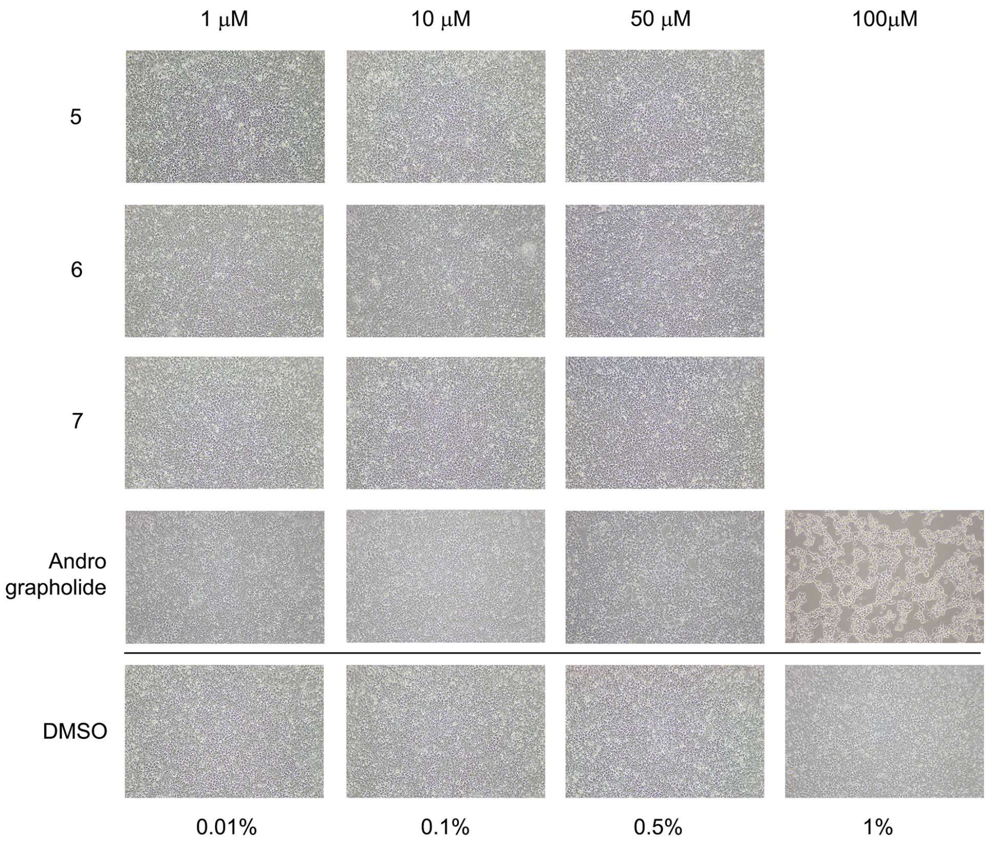

Figure S1. The evaluation of cytotoxicity of scequinadolines A (**5**), quinadoline A (**6**) and scequinadolines E (**7**) was observed of alterations of cell morphology. HEK293T/17 cells were incubated with 1, 10 and 50 mM of TL01, 02, 03, in parallel with andrographolide and DMSO as a control. At 24 hrs post treatment, cell morphology of the treated cells was observed under inverted light microscope.

Figure S2. HR-ESI-MS spectrum of dichocetide B (**1**)

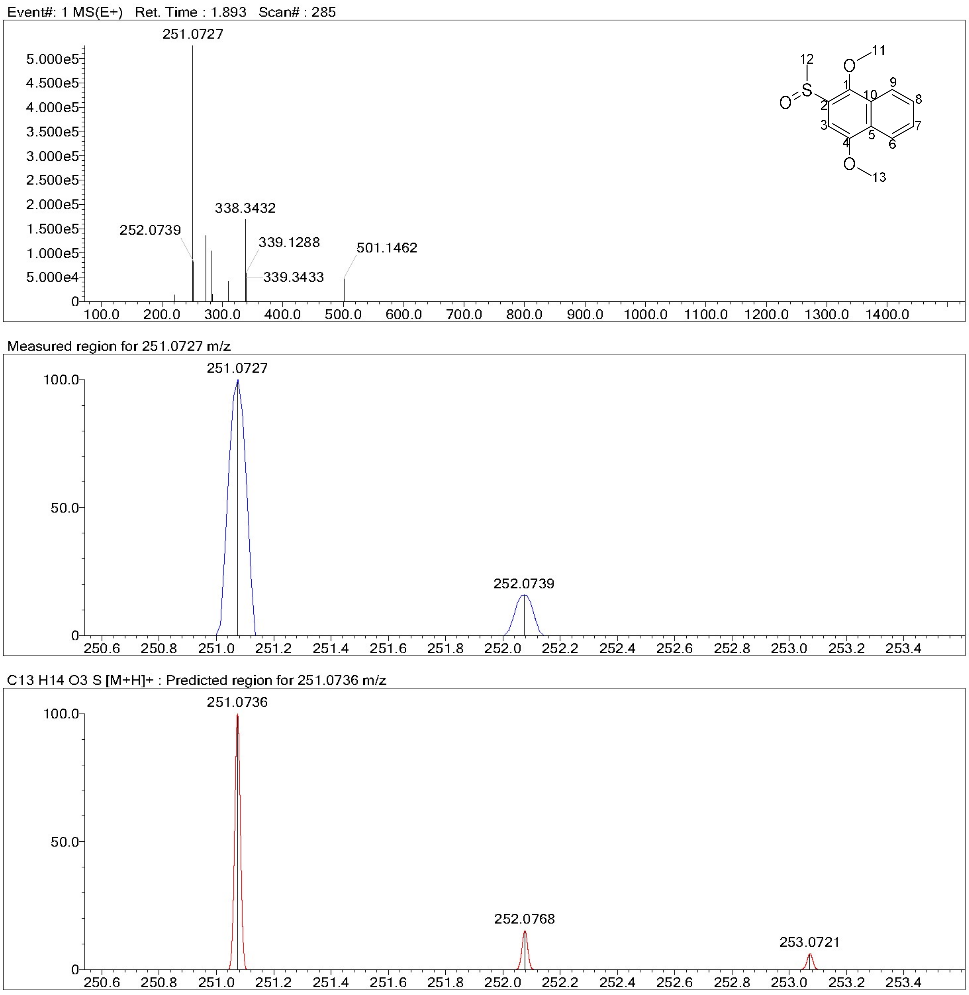

Rank	Score	Formula (M)	Ion	Meas. m/z	Pred. m/z	Df. (mDa)	Df. (ppm)	Iso	DBE
3	77.10	C13 H14 O3 S	[M+H]+	251.0727	251.0736	-0.9	-3.58	82.41	7.0

Mar. Drugs **2018**, *16*, 229; doi:10.3390/md16070229 www.mdpi.com/journal/marinedrugs

Figure S3. ^{1}H NMR spectrum of dichocetide B (**1**) in $CDCl_3$ (400MHz)

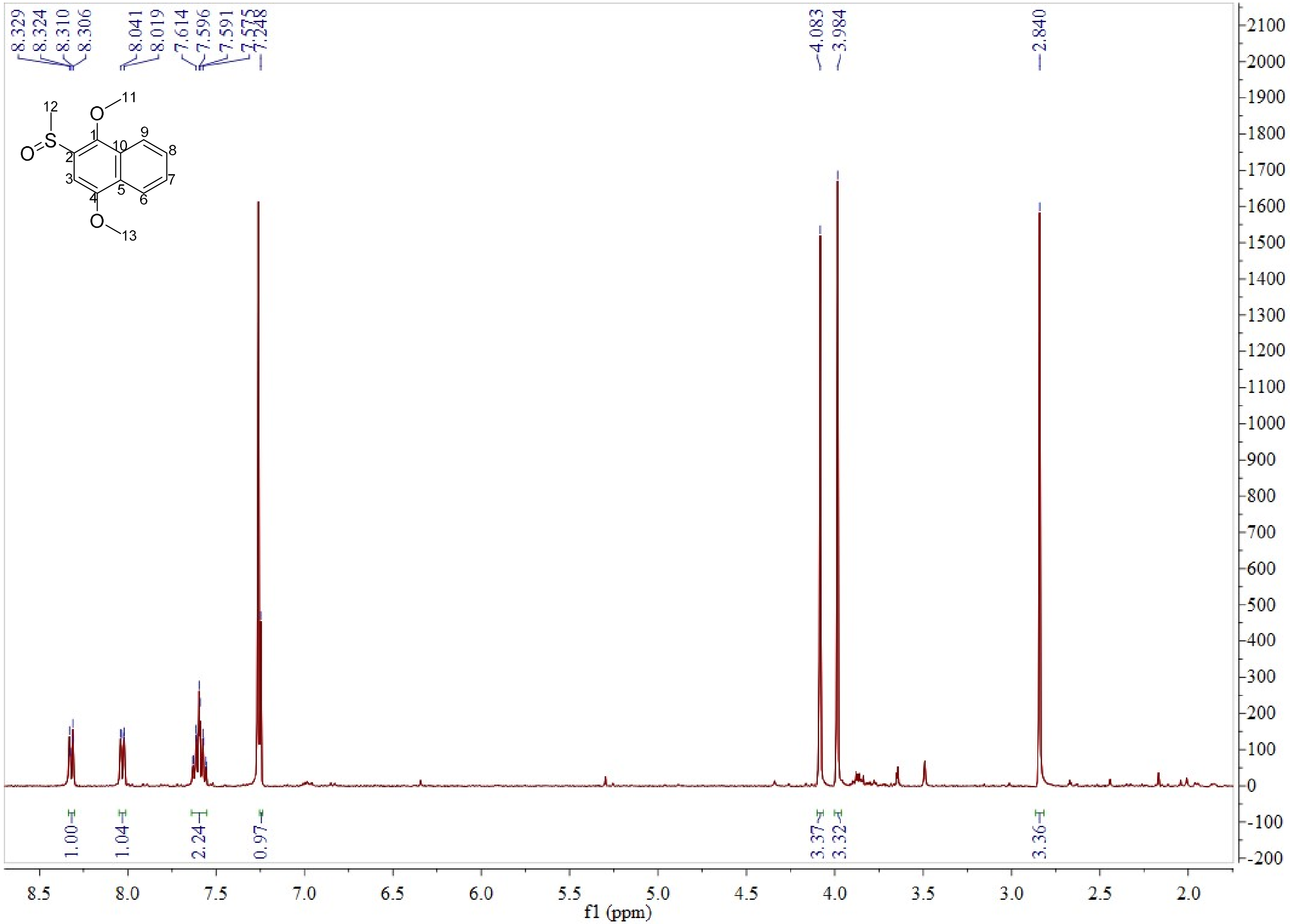

Figure S4. An expansion of ^{1}H NMR spectrum of dichocetide B (**1**) in $CDCl_3$ (400MHz)

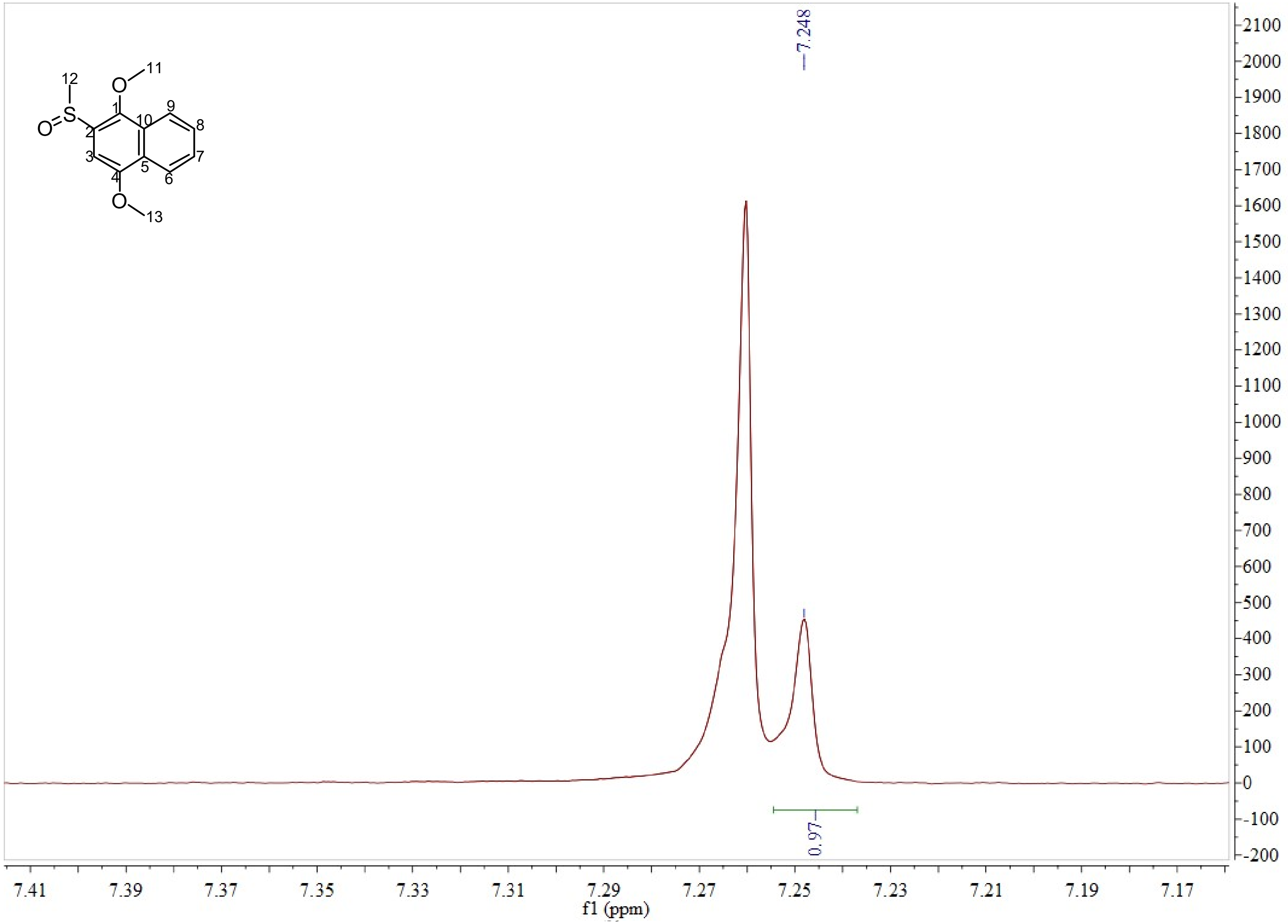

Figure S5. ^{13}C NMR spectrum of dichocetide B (**1**) in $CDCl_3$ (100MHz)

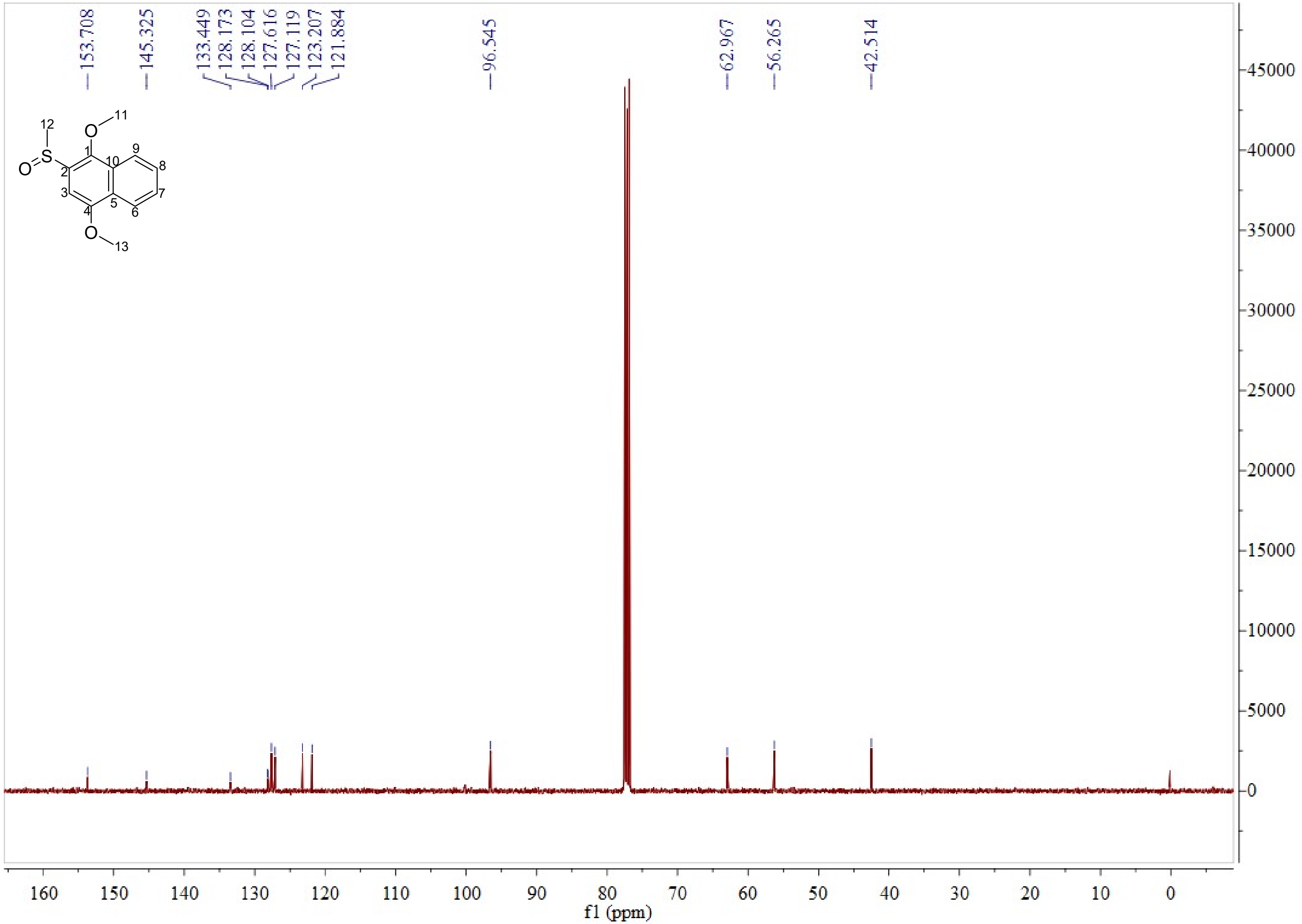

Figure S6. An expansion of ^{13}C NMR spectrum of dichocetide B (**1**) in $CDCl_3$ (100MHz)

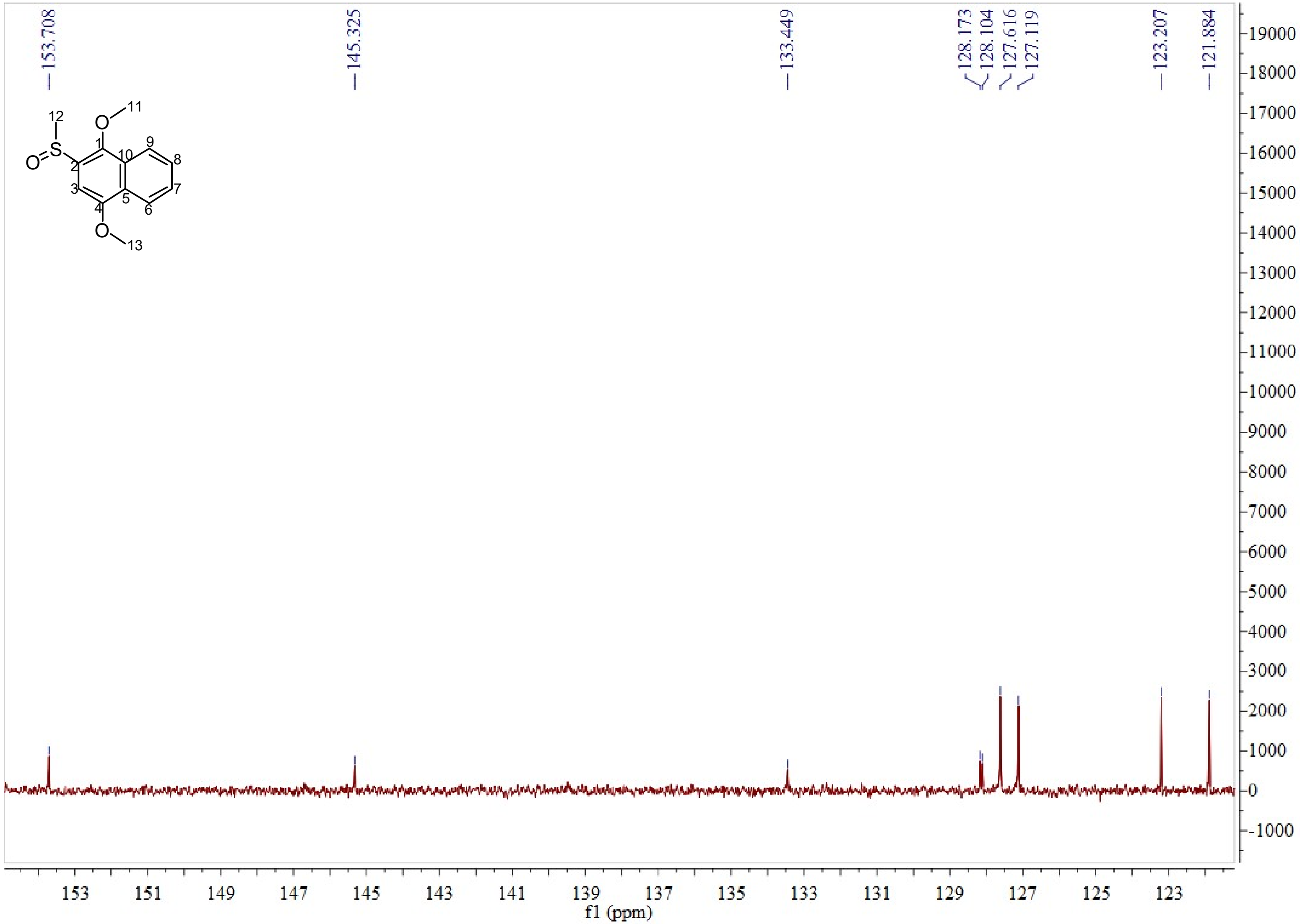

Figure S7. DEPT 135 spectrum of dichocetide B (**1**) in $CDCl_3$ (100MHz)

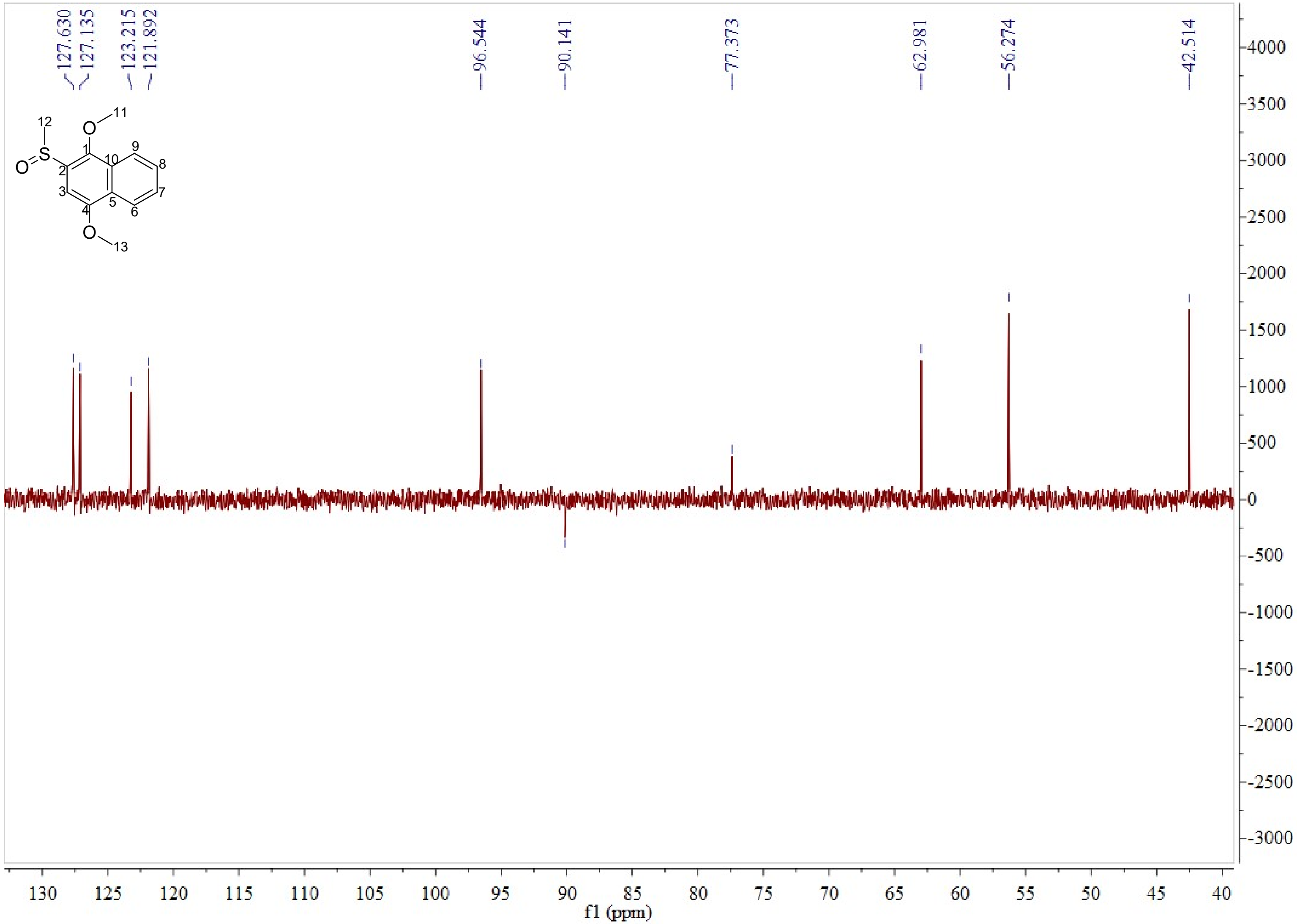

Figure S8. HSQC spectrum of dichocetide B (**1**) in $CDCl_3$

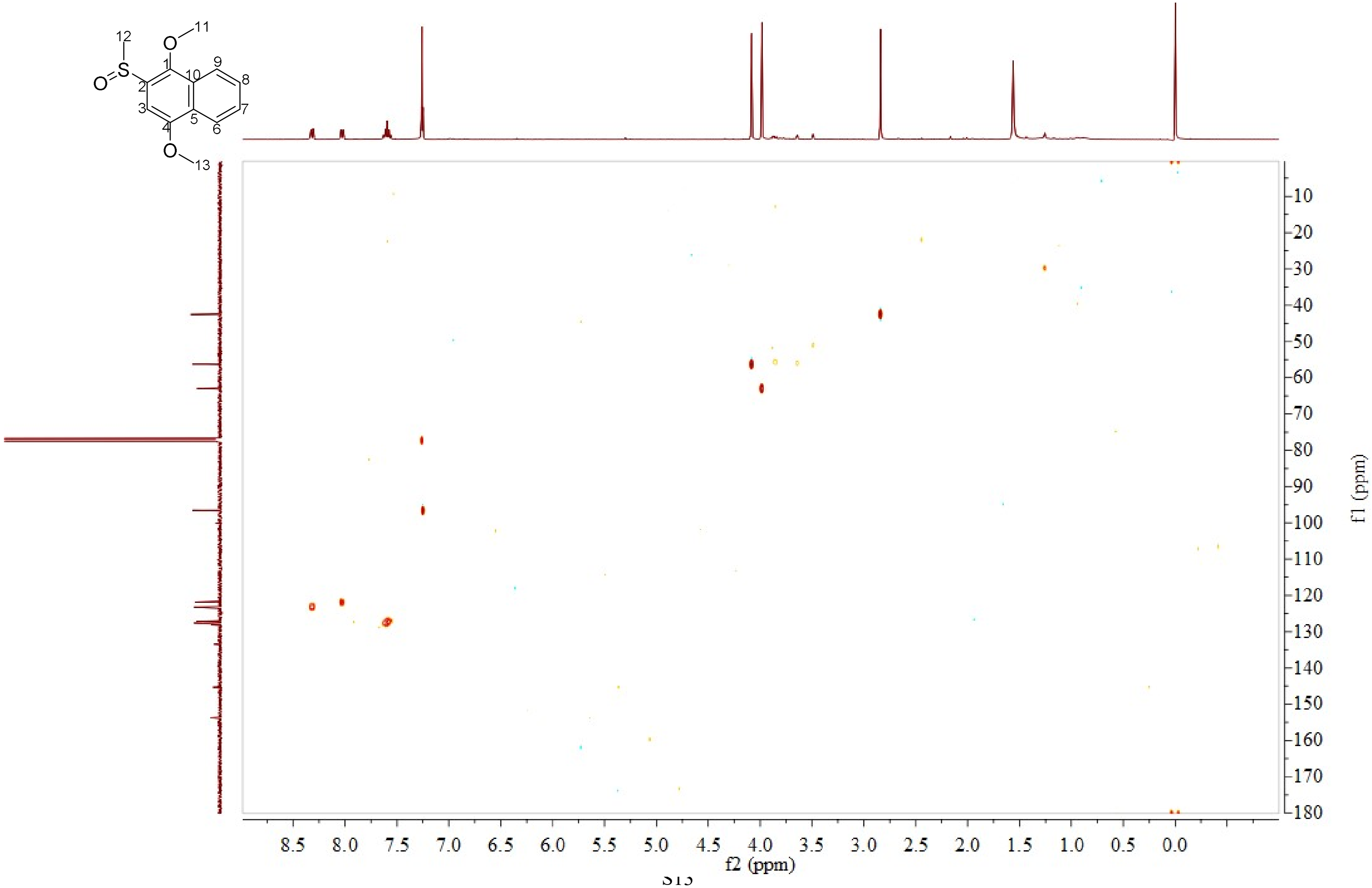

S13

Figure S9. ^{1}H-^{1}H COSY spectrum of dichocetide B (**1**) in $CDCl_3$

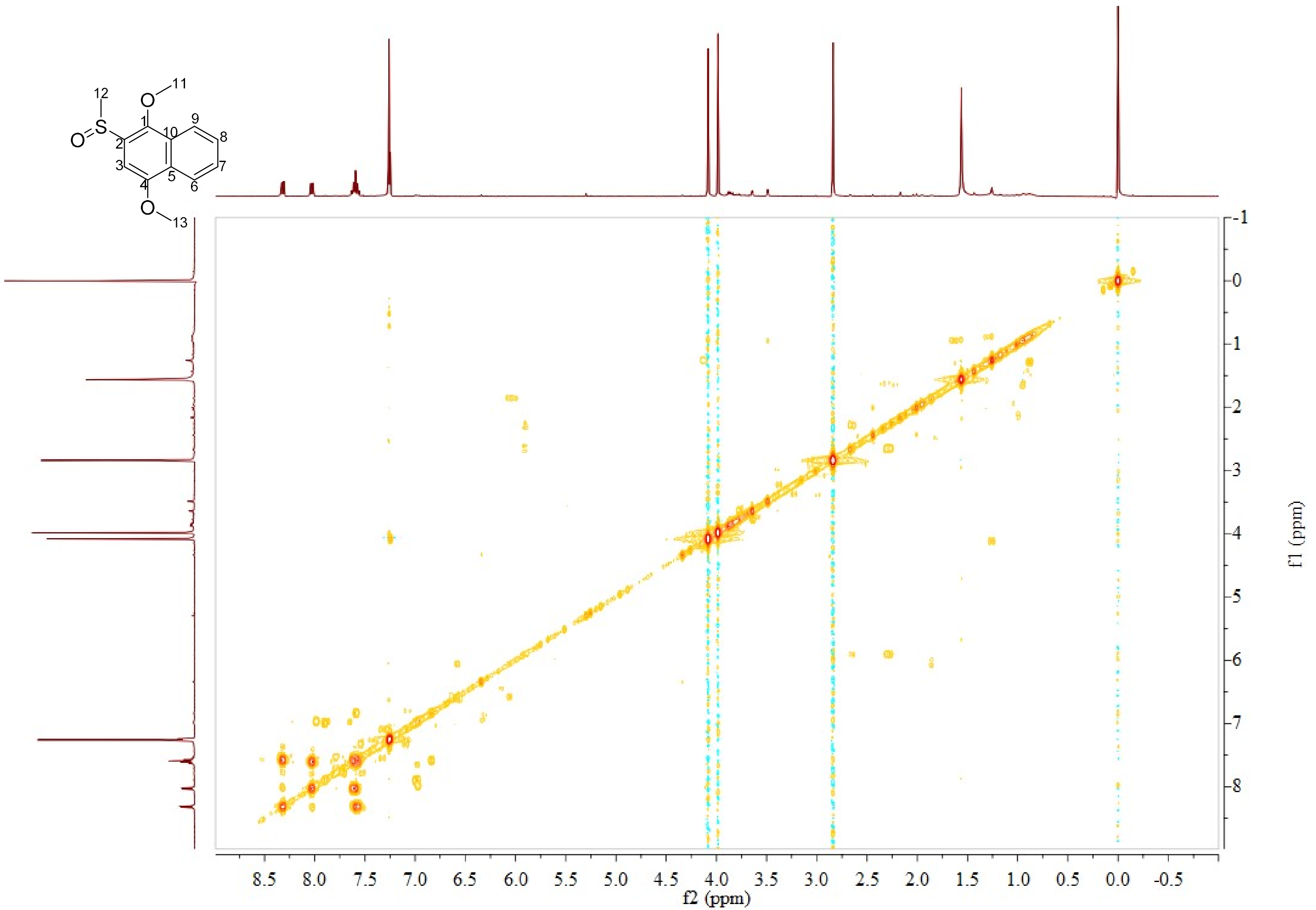

Figure S10. HMBC spectrum of dichocetide B (**1**) in $CDCl_3$

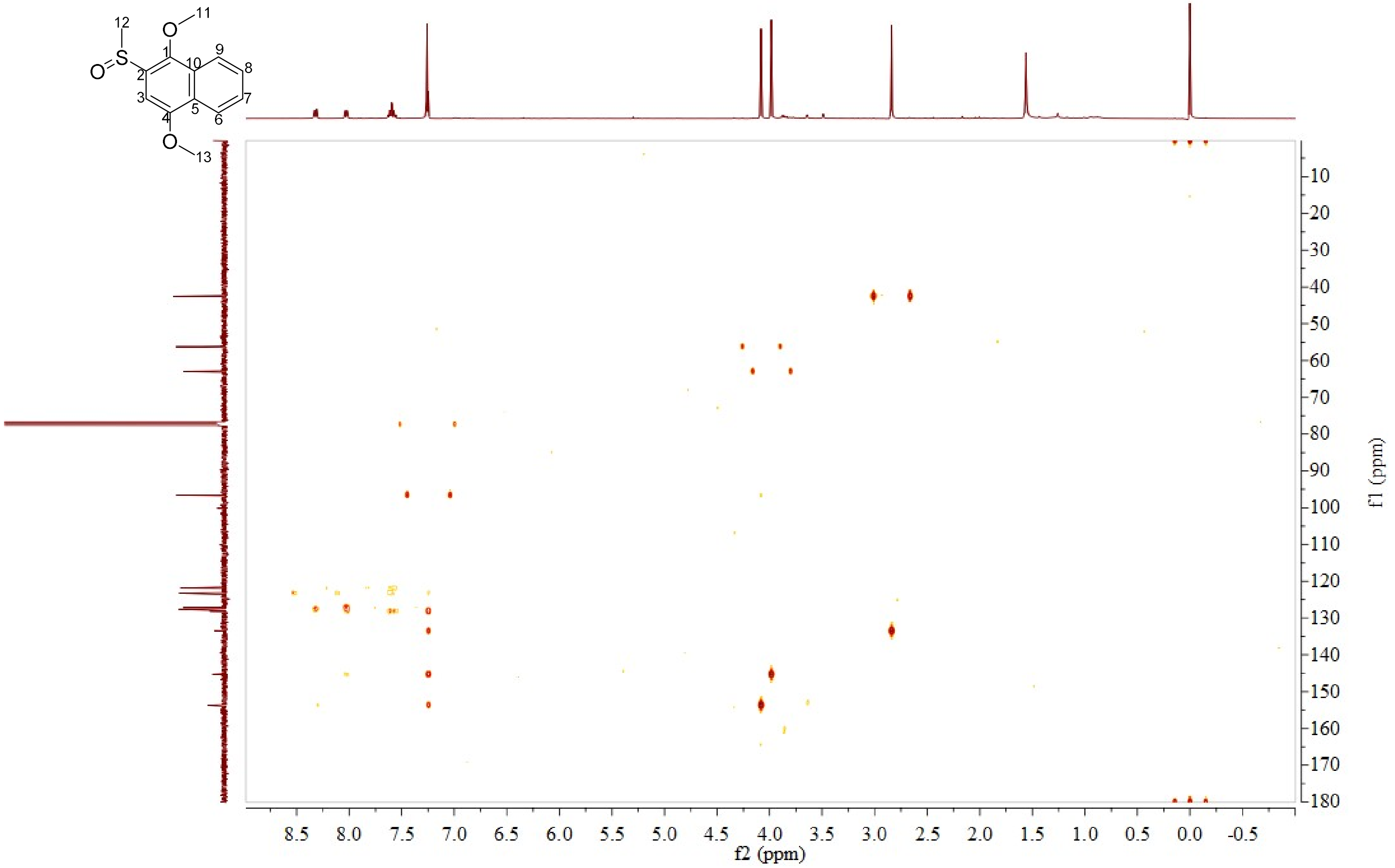

Figure S11. An expansion of HMBC spectrum of dichocetide B (**1**) in $CDCl_3$

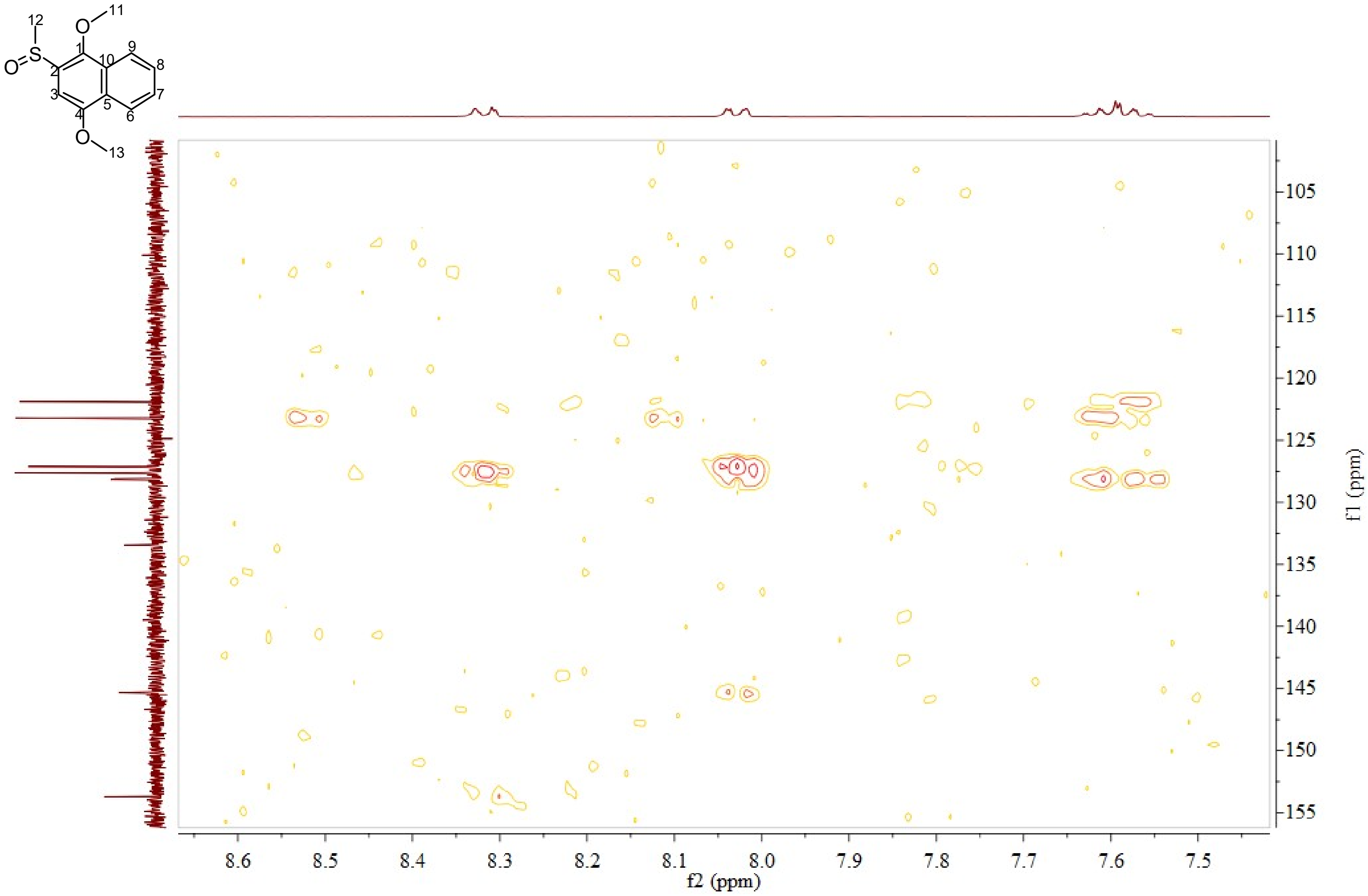

Figure S12. NOESY spectrum of dichocetide B (**1**) in $CDCl_3$

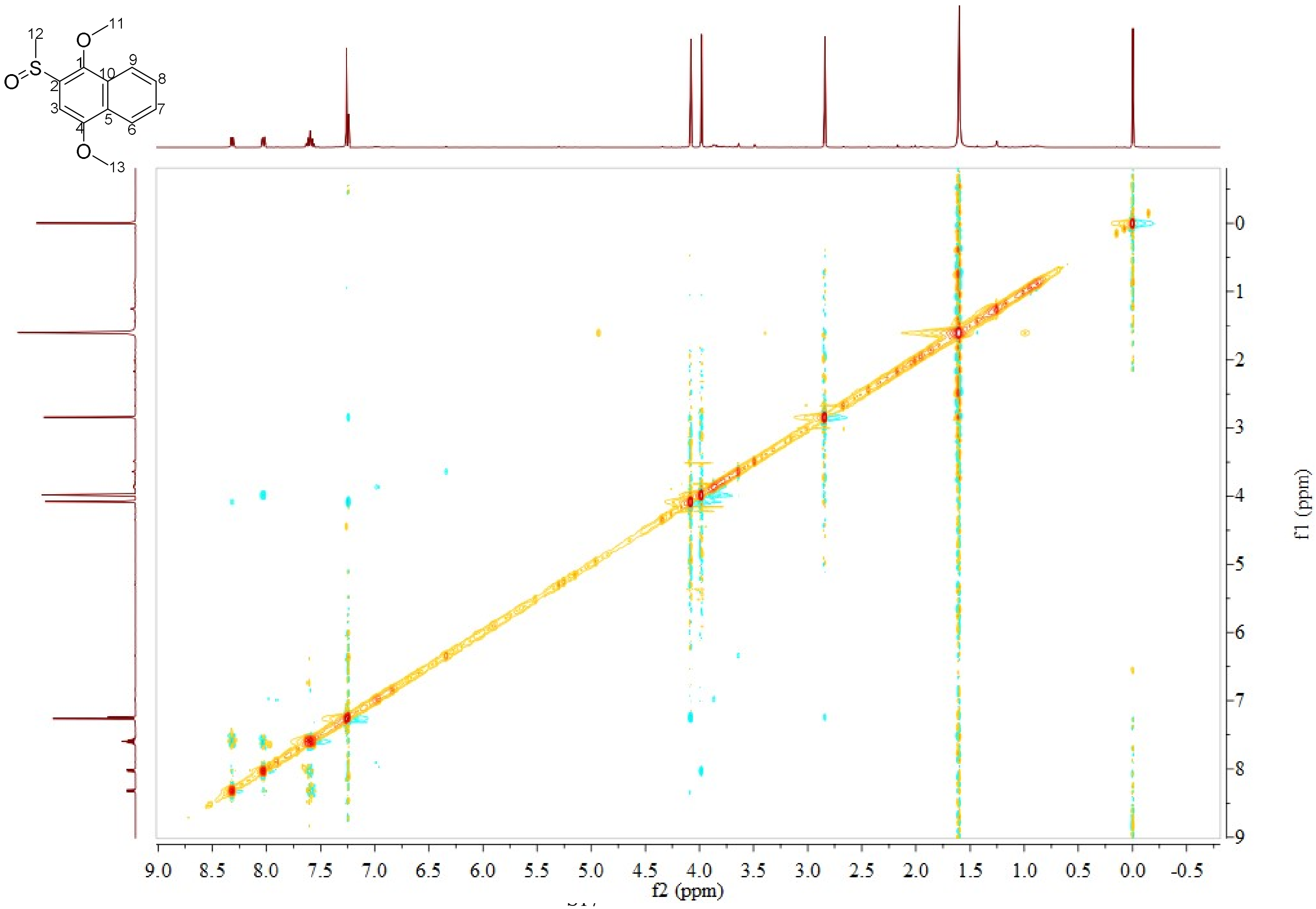

Figure S13. HR-ESI-MS spectrum of dichocetide C (**2**)

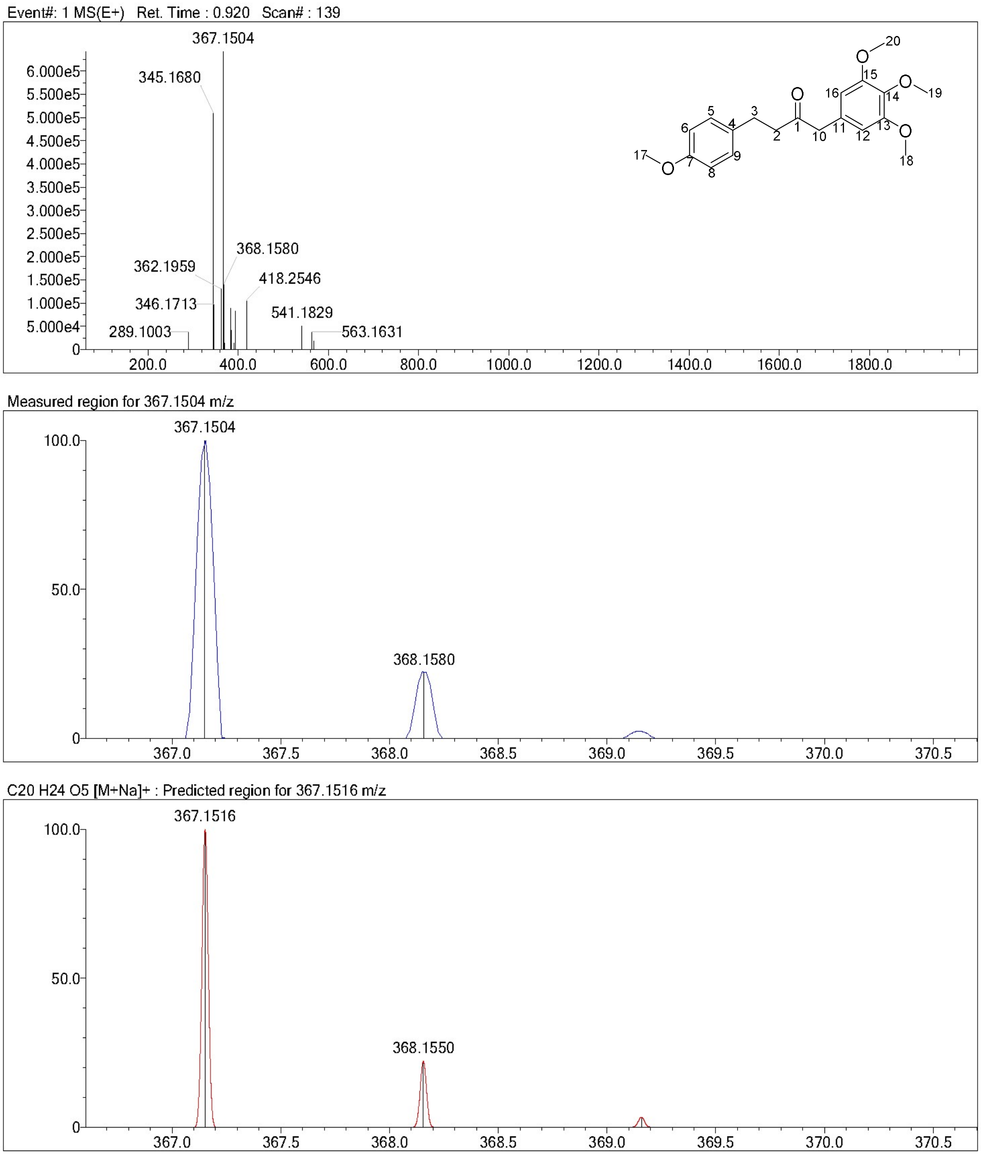

Rank	Score	Formula (M)	Ion	Meas. m/z	Pred. m/z	Df. (mDa)	Df. (ppm)	Iso	DBE
2	74.53	C20 H24 O5	[M+Na]+	367.1504	367.1516	-1.2	-3.27	79.01	9.0

Figure S14. ^{1}H NMR spectrum of dichocetide C (**2**) in $CDCl_3$ (400MHz)

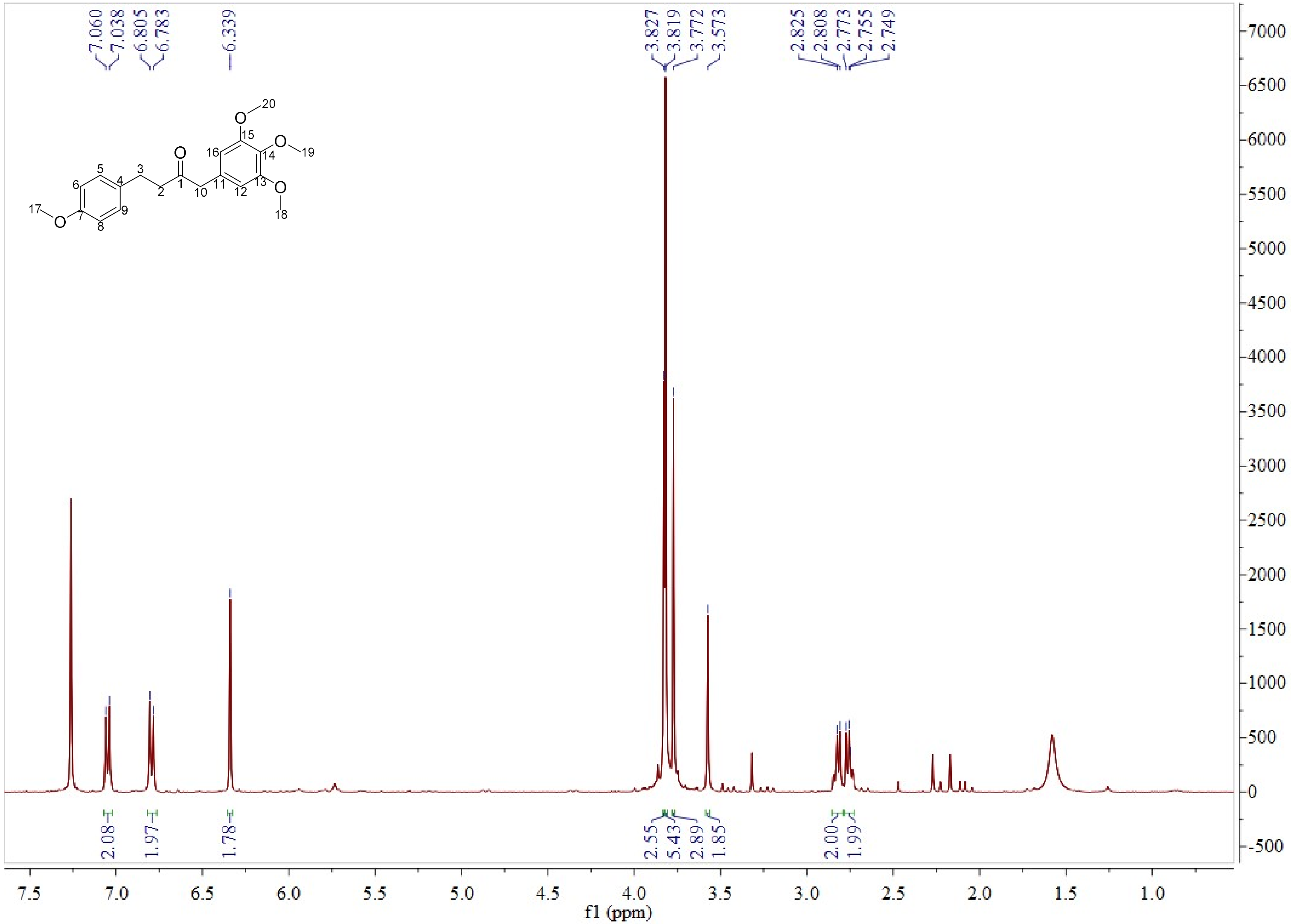

Figure S15. 13 C NMR spectrum of dichocetide C (**2**) in $CDCl_3$ (100MHz)

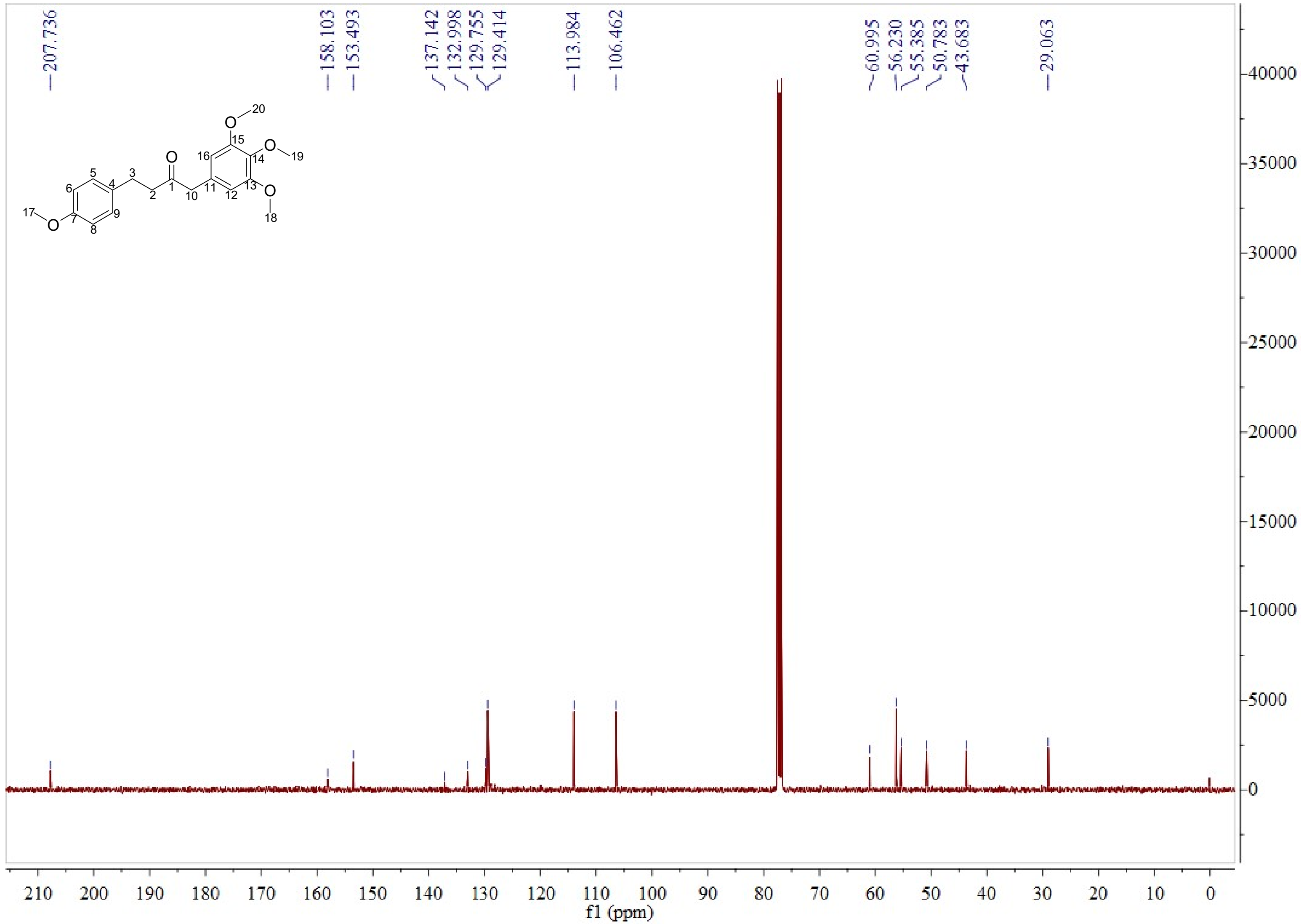

Figure S16. DEPT 135 spectrum of dichocetide C (**2**) in $CDCl_3$ (100MHz)

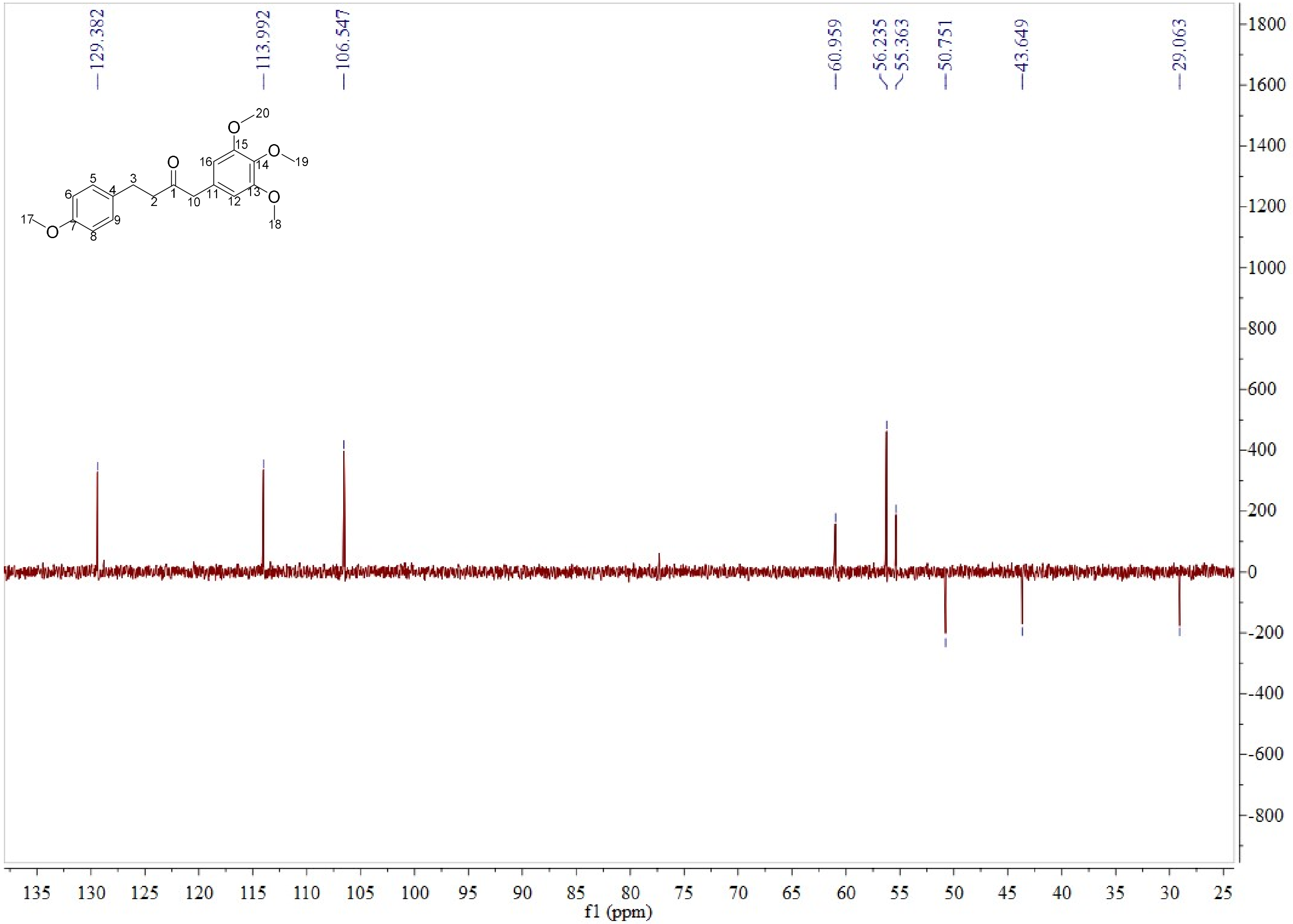

Figure S17. HSQC spectrum of dichocetide C (**2**) in $CDCl_3$

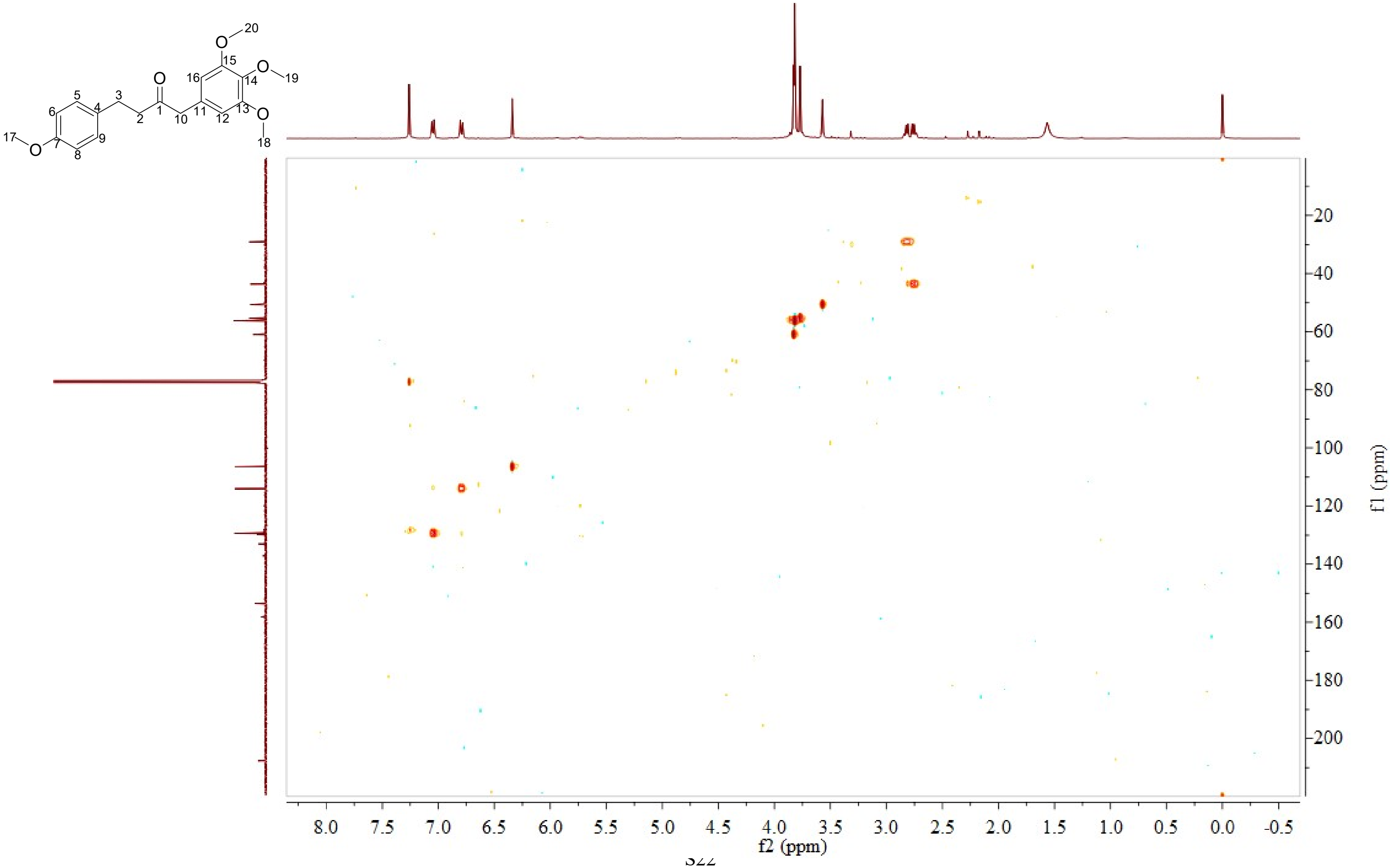

Figure S18. ^1H-^1H COSY spectrum of dichocetide C (**2**) in $CDCl_3$

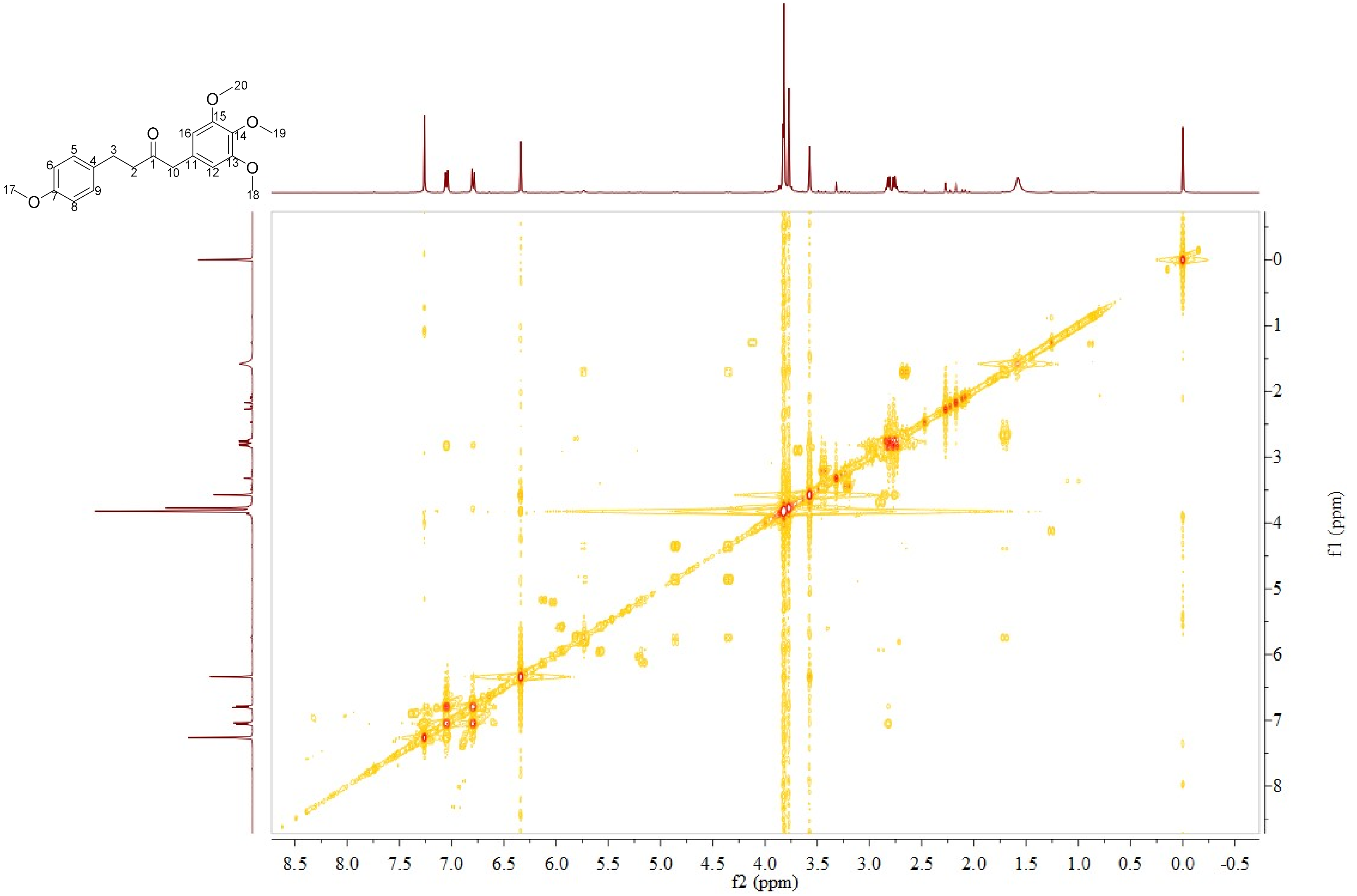

Mar. Drugs **2018**, *16*, 229; doi:10.3390/md16070229 www.mdpi.com/journal/marinedrugs

Figure S19. HMBC spectrum of dichocetide C (**2**) in $CDCl_3$

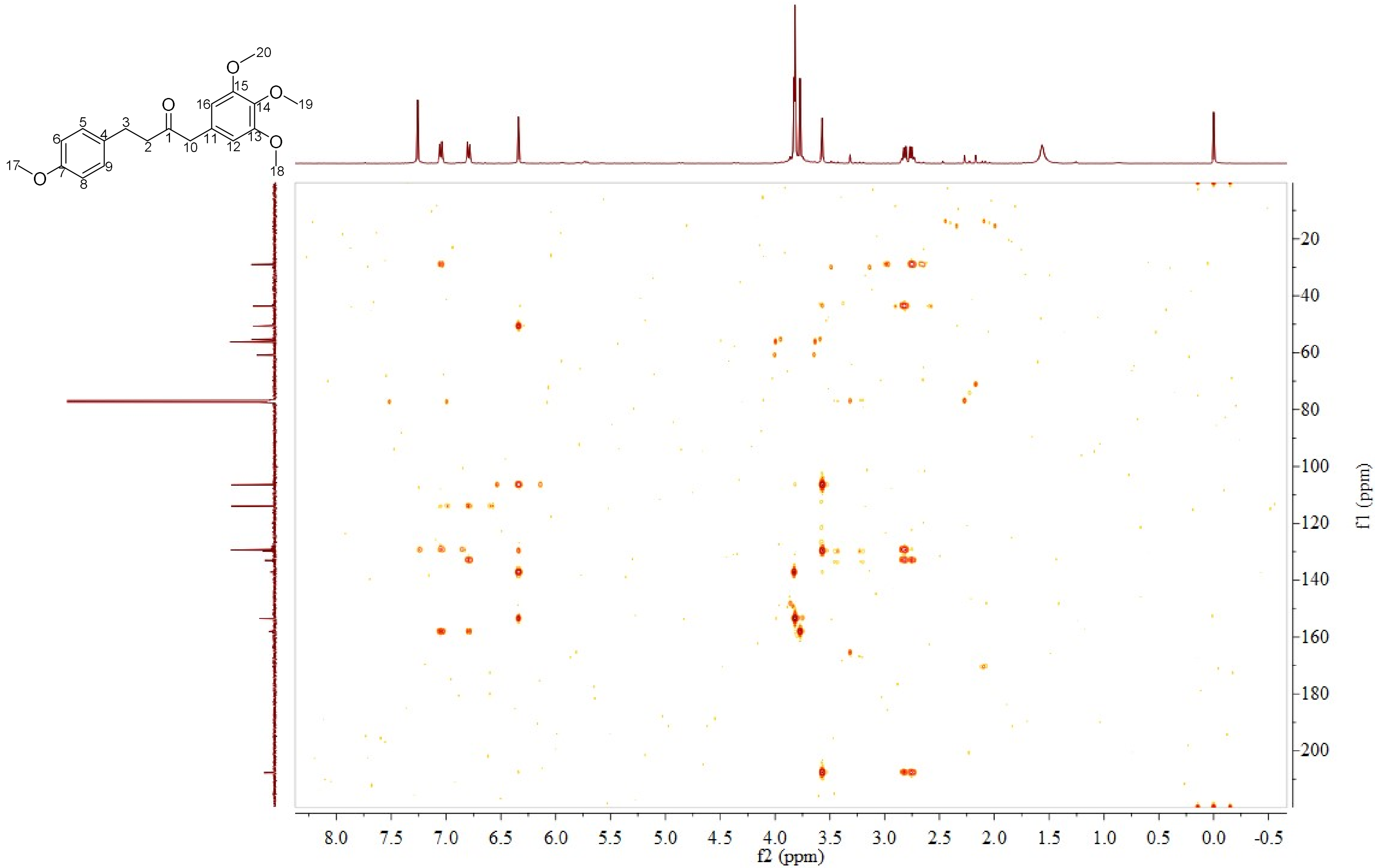

Figure S20. NOESY spectrum of dichocetide C (**2**) in $CDCl_3$

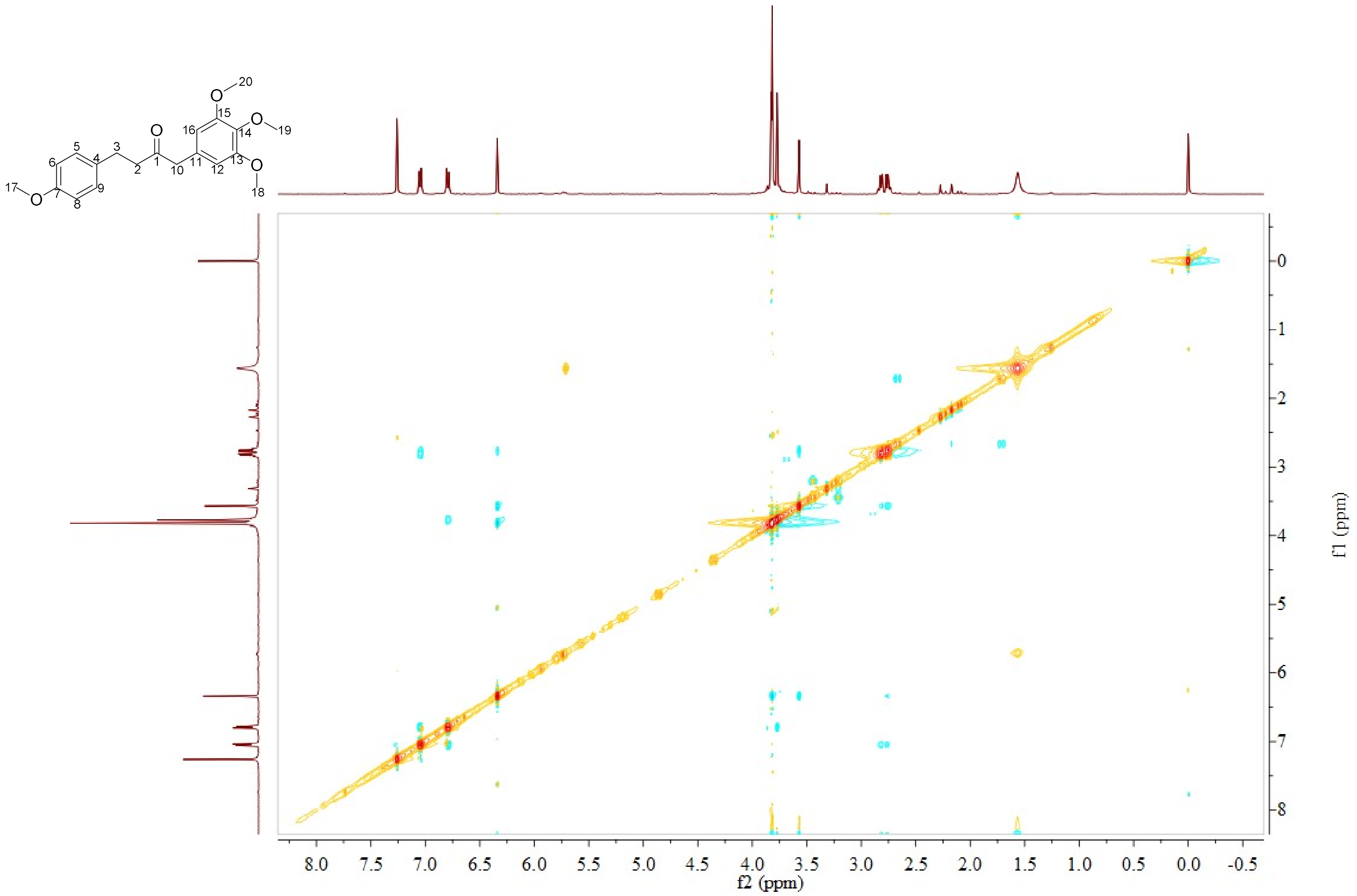

Figure S21. HR-ESI-MS spectrum of dichotomocej E (**3**)

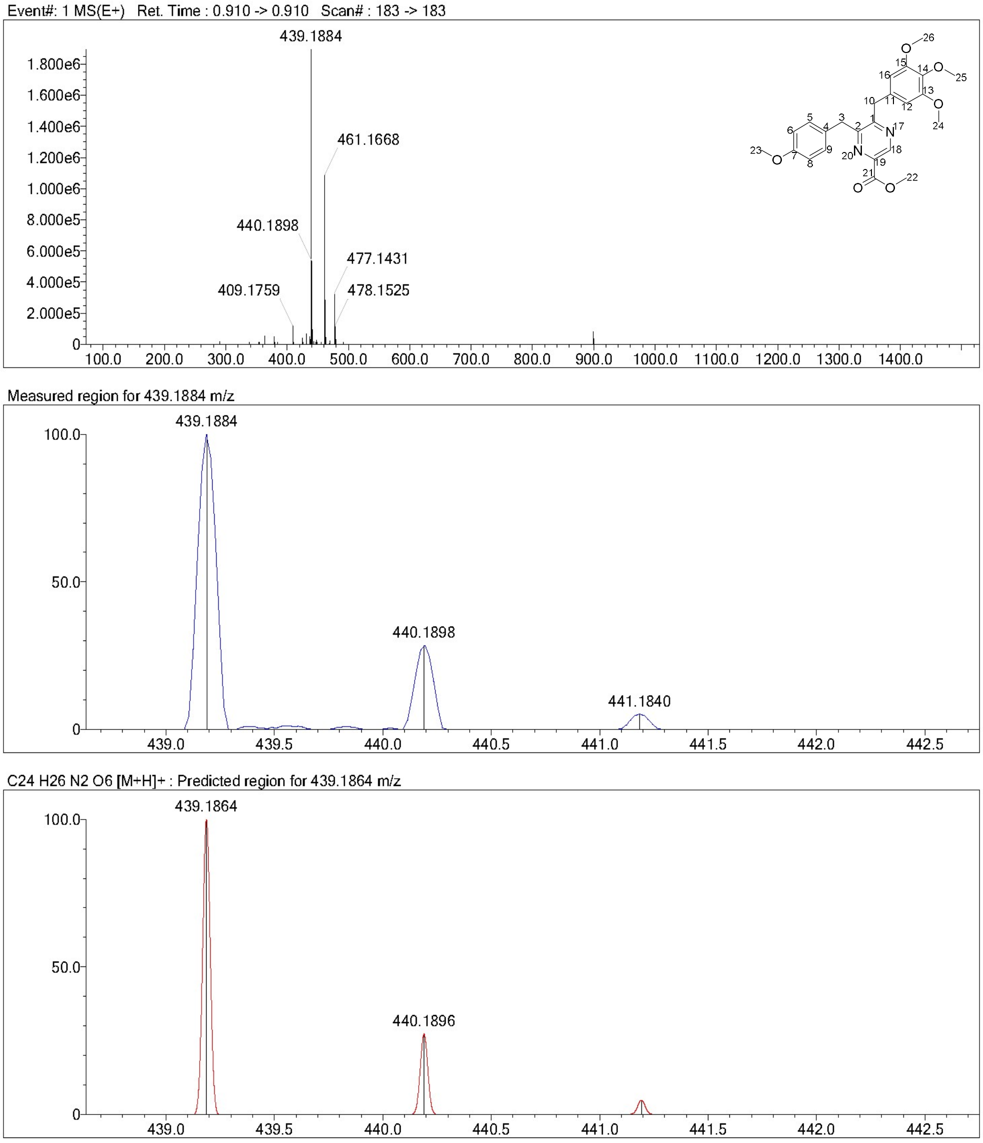

Rank	Score	Formula (M)	Ion	Meas. m/z	Pred. m/z	Df. (mDa)	Df. (ppm)	Iso	DBE
7	77.54	C24 H26 N2 O6	[M+H]+	439.1884	439.1864	2.0	4.55	85.10	13.0

Figure S22. ^{1}H NMR spectrum of dichotomocej E (**3**) in $CDCl_3$ (400MHz)

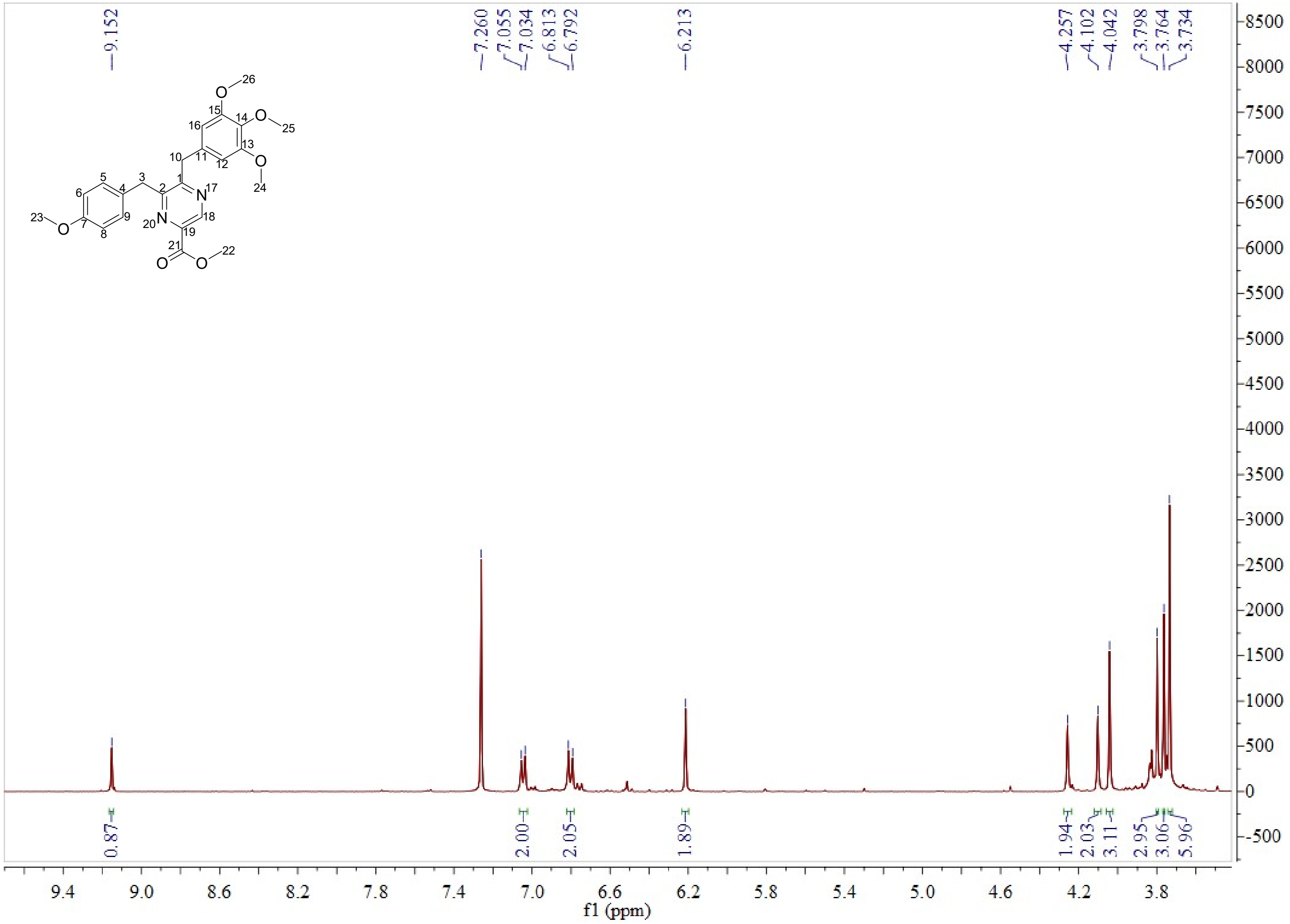

Figure S23. ^{13}C NMR spectrum of dichotomocej E (**3**) in $CDCl_3$ (100MHz)

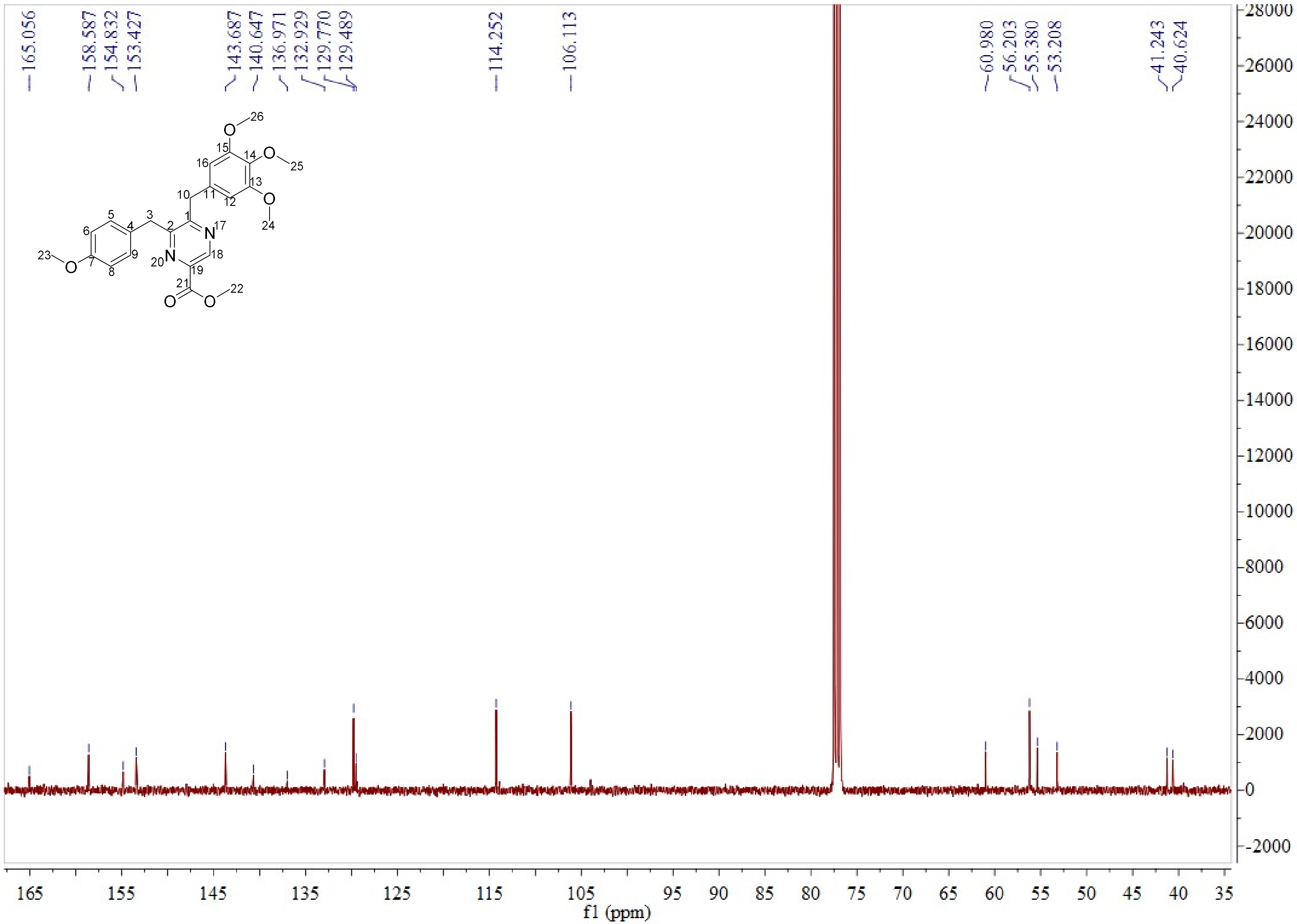

Figure S24. ^{1}H NMR spectrum of dichotomocej E (**3**) in $CDCl_3$ (400MHz)

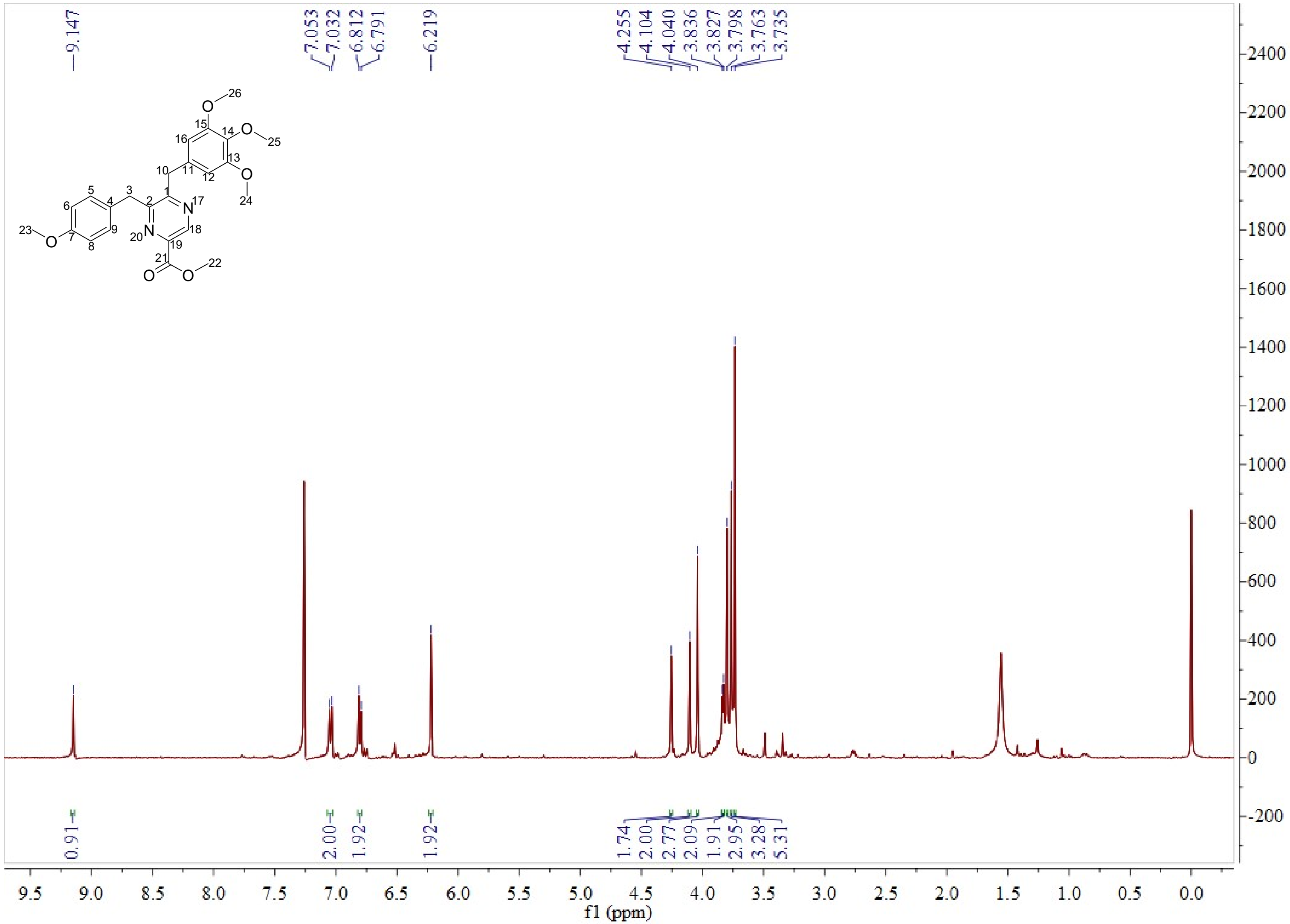

Figure S25. ^{13}C NMR spectrum of dichotomocej E (**3**) in $CDCl_3$ (100MHz)

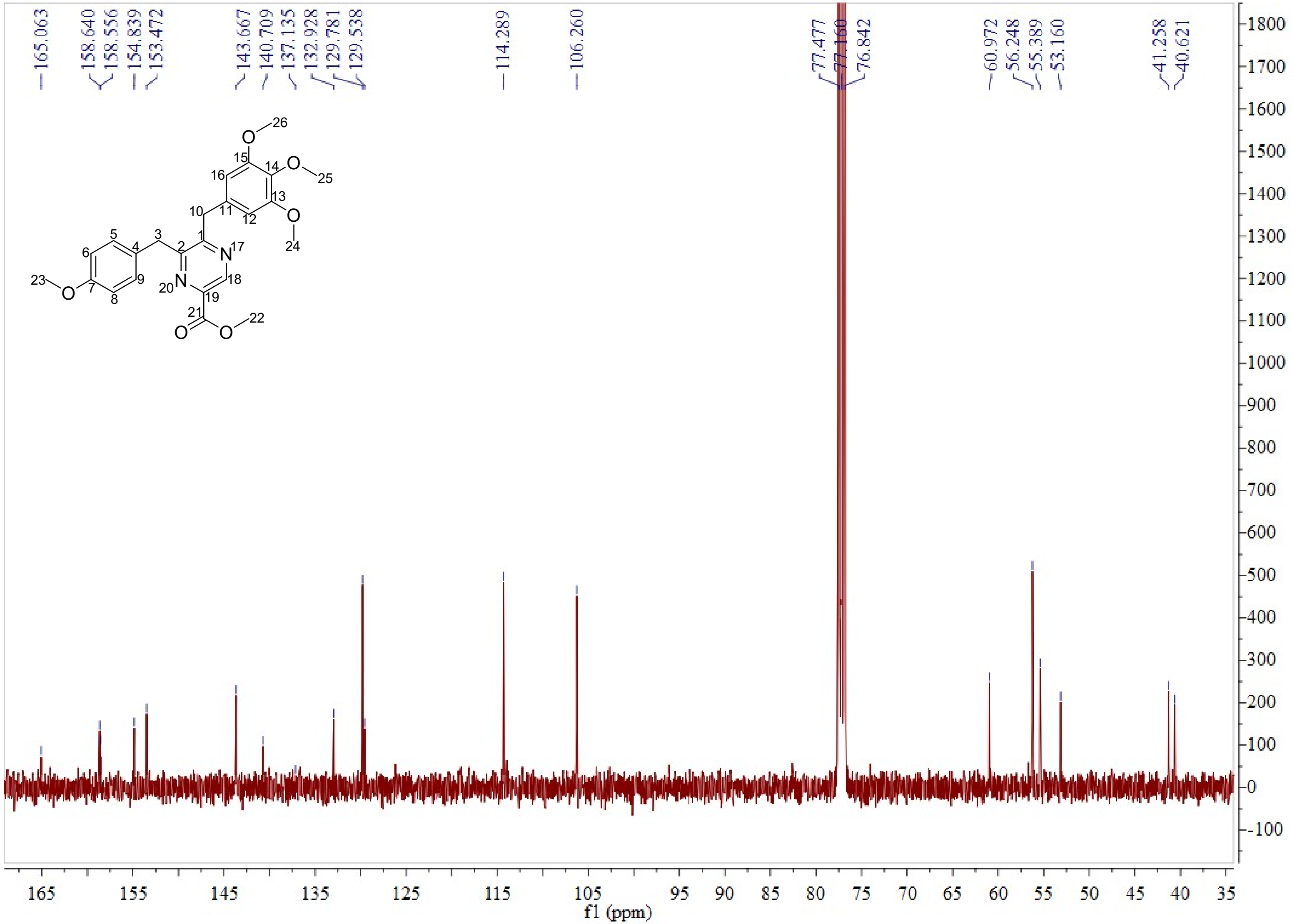

Mar. Drugs **2018**, *16*, 229; doi:10.3390/md16070229 www.mdpi.com/journal/marinedrugs

Figure S26. An expansion of ^{13}C NMR spectrum of dichotomocej E (**3**) in $CDCl_3$ (100MHz)

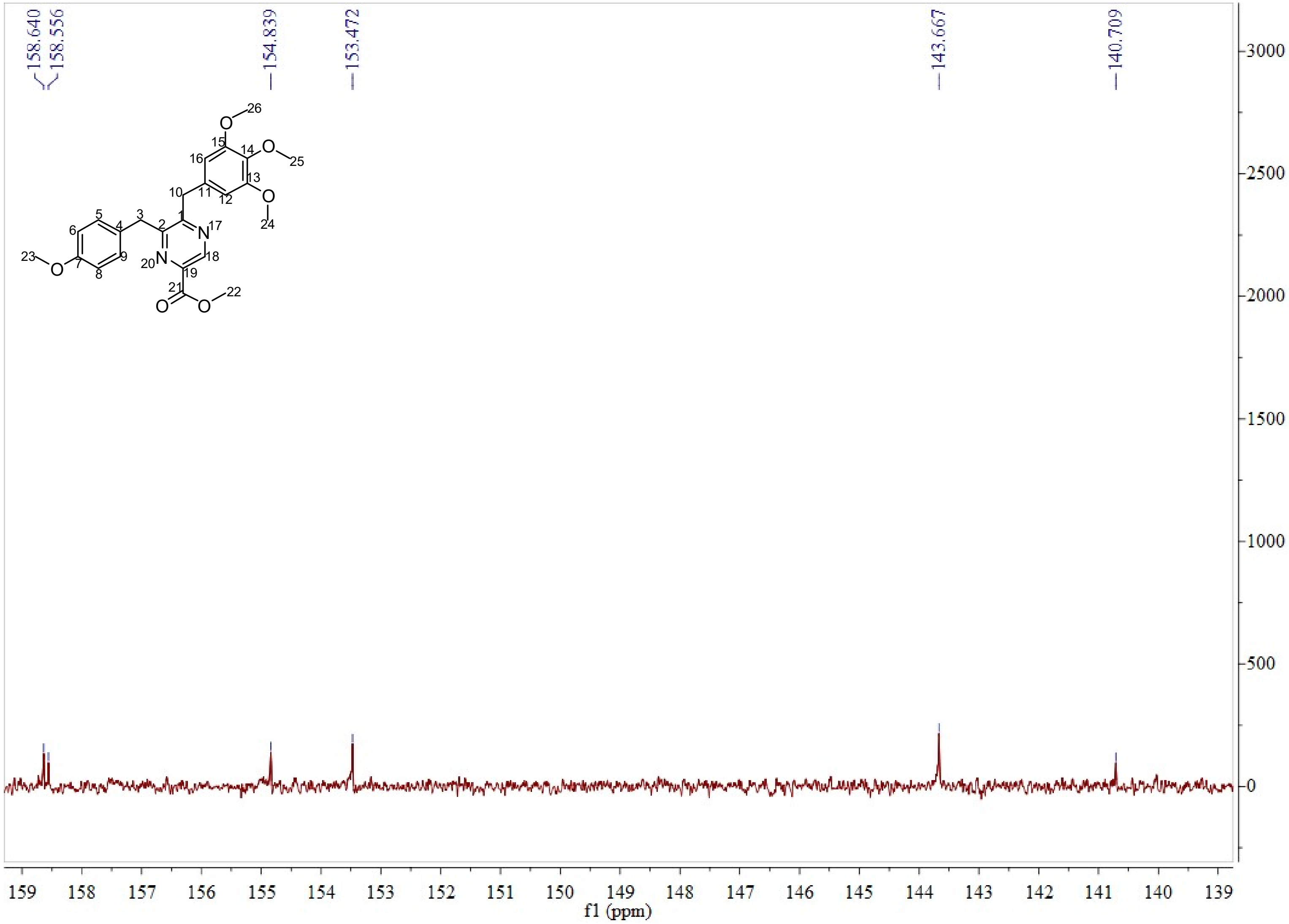

Figure S27. DEPT 135 spectrum of dichotomocej E (**3**) in $CDCl_3$ (100MHz)

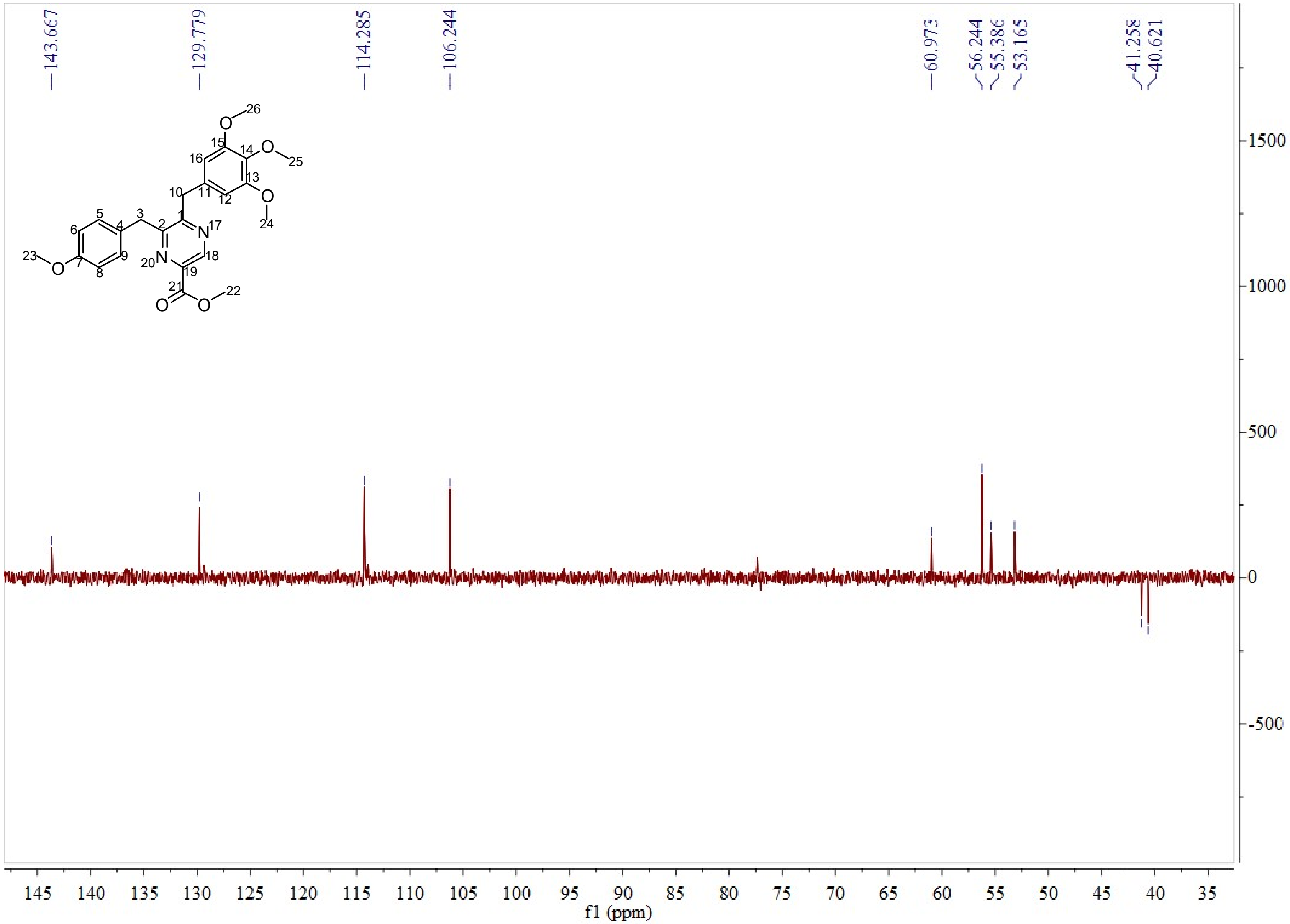

Figure S28. HSQC spectrum of dichotomocej E (**3**) in $CDCl_3$

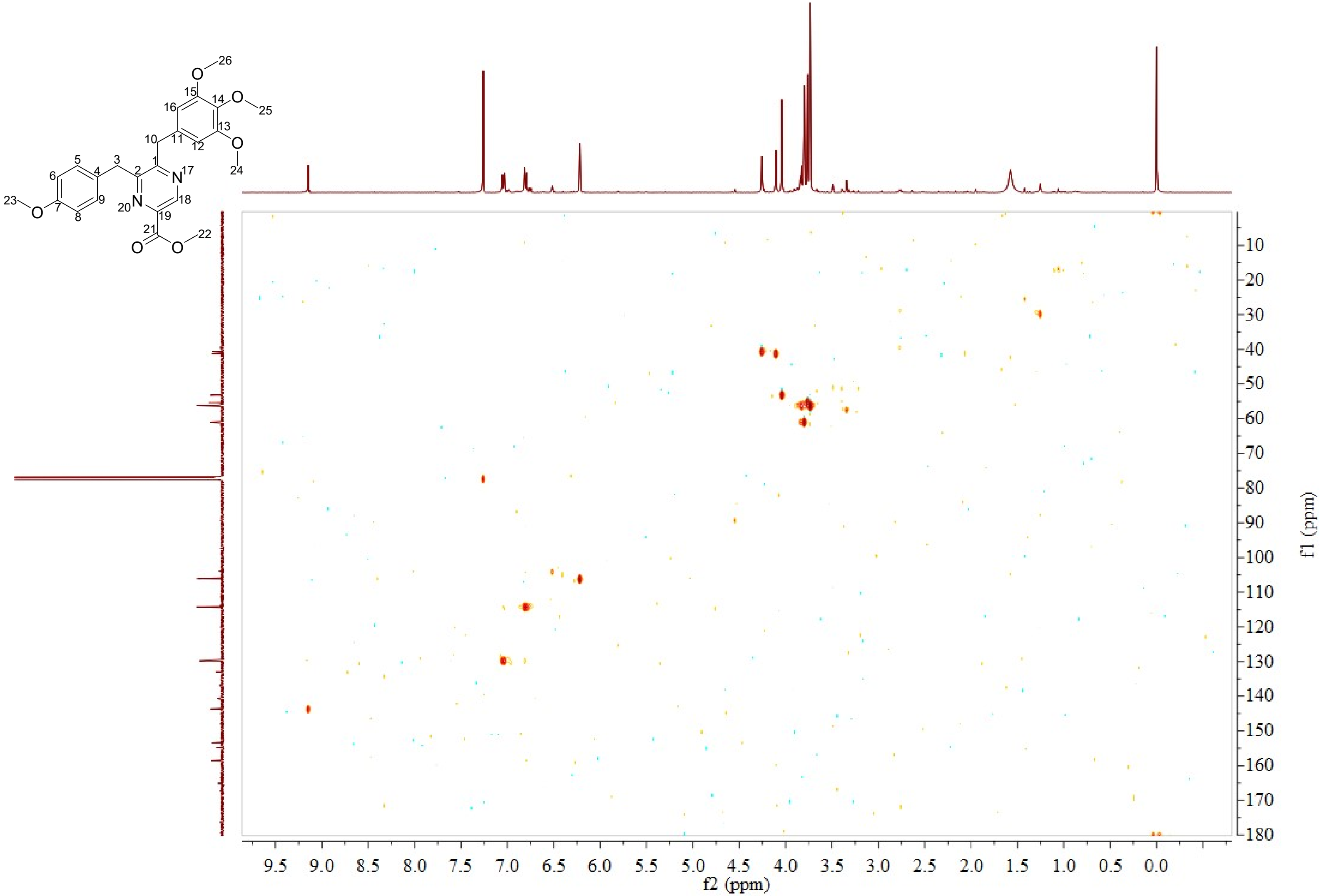

Figure S29. ^{1}H-^{1}H COSY spectrum of dichotomocej E (**3**) in $CDCl_3$

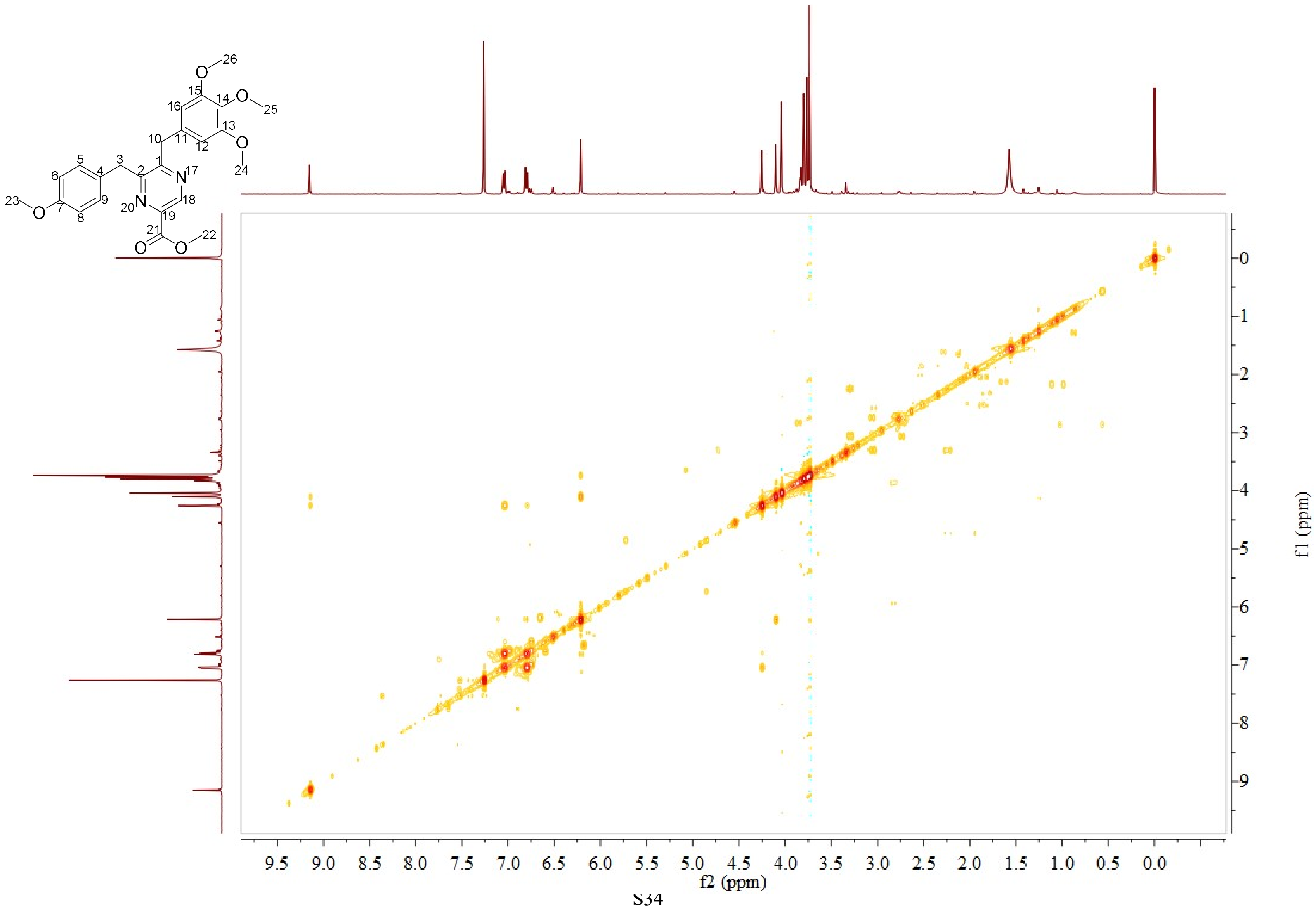

Figure S30. HMBC spectrum of dichotomocej E (**3**) in $CDCl_3$

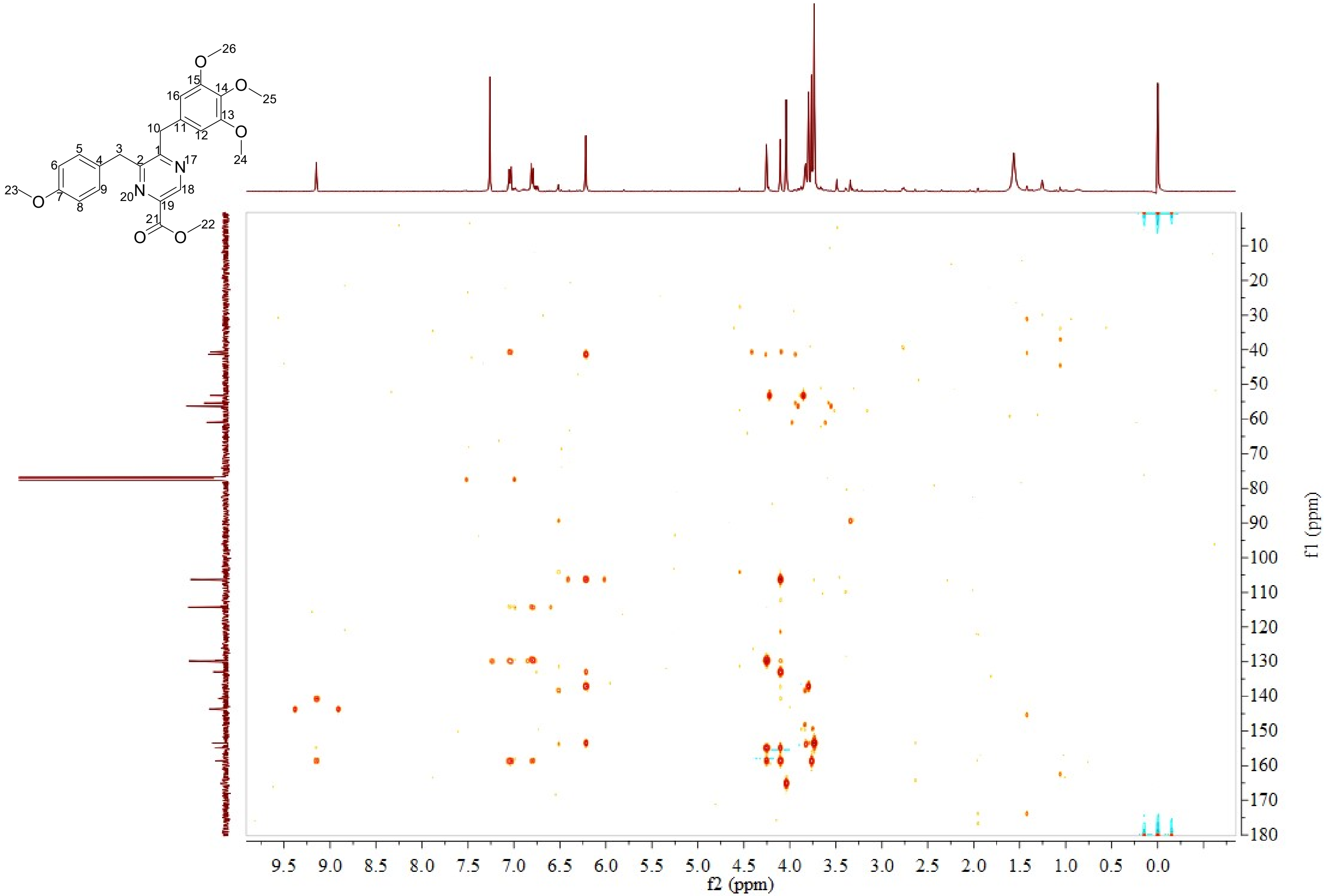

Figure S31. NOESY spectrum of dichotomocej E (**3**) in $CDCl_3$

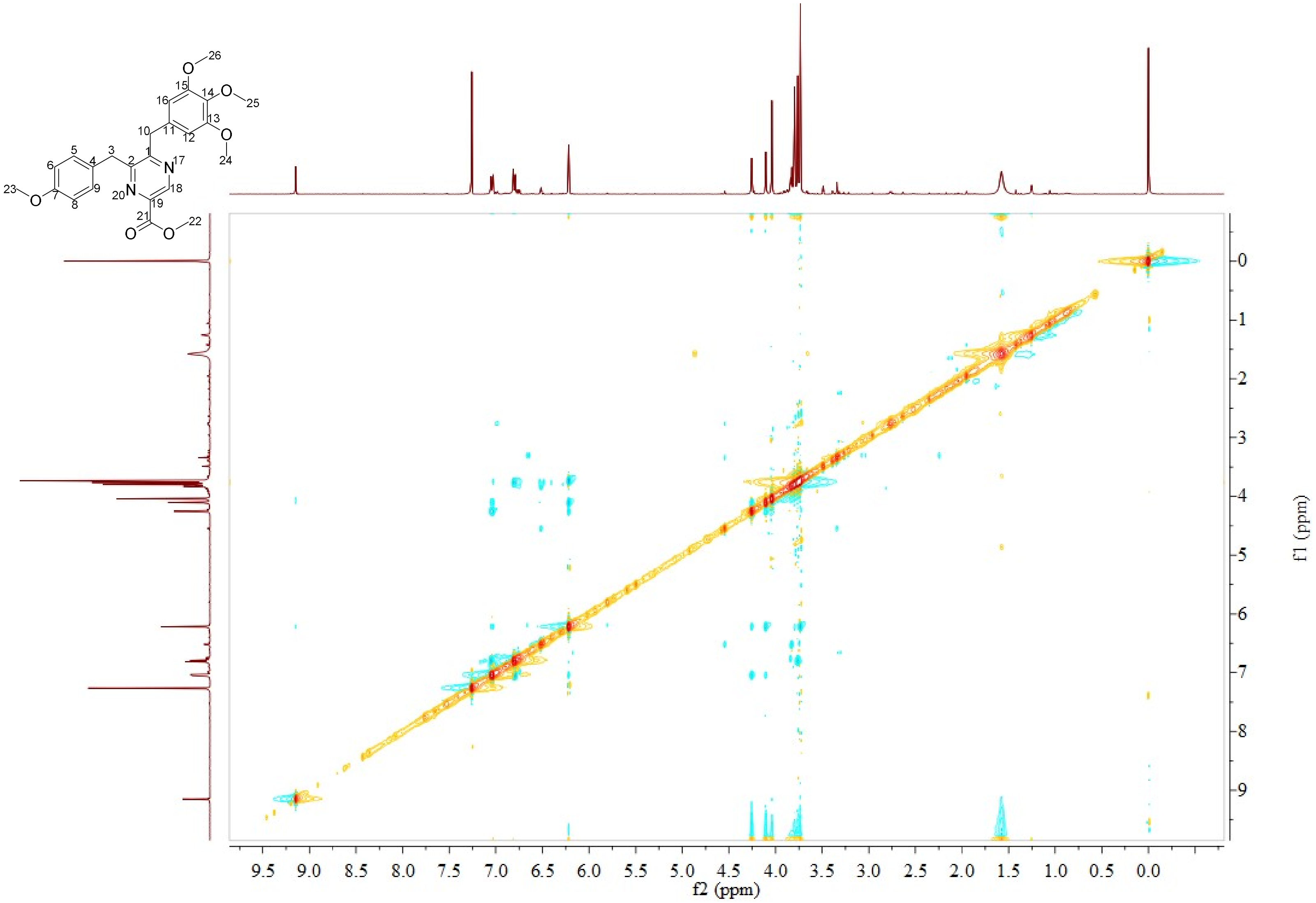

Figure S32. ^{1}H NMR spectrum of dichotomocej E (**3**) in $CDCl_3$ (500MHz)

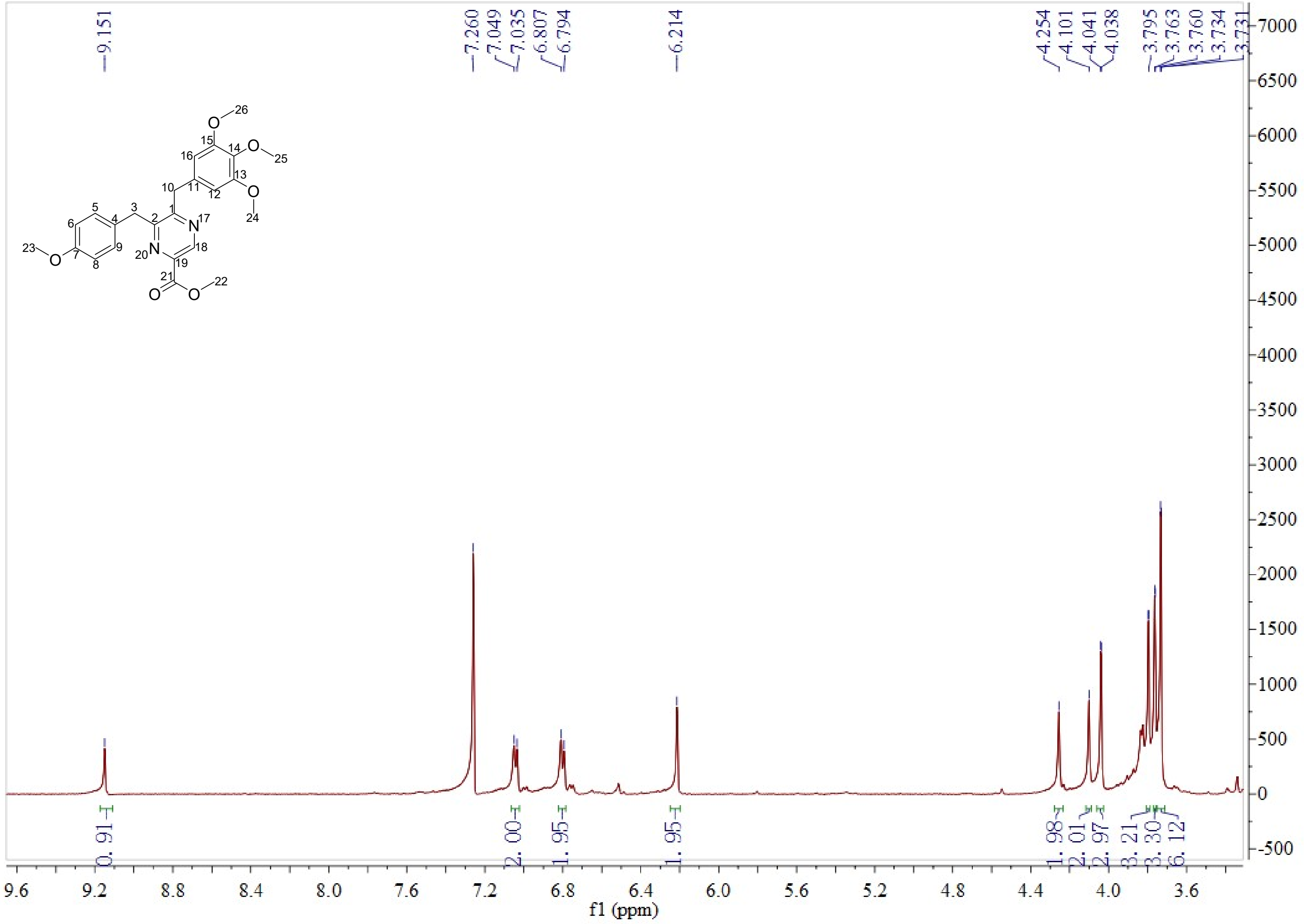

Figure S33. ^{13}C NMR spectrum of dichotomocej E (**3**) in $CDCl_3$ (125Hz)

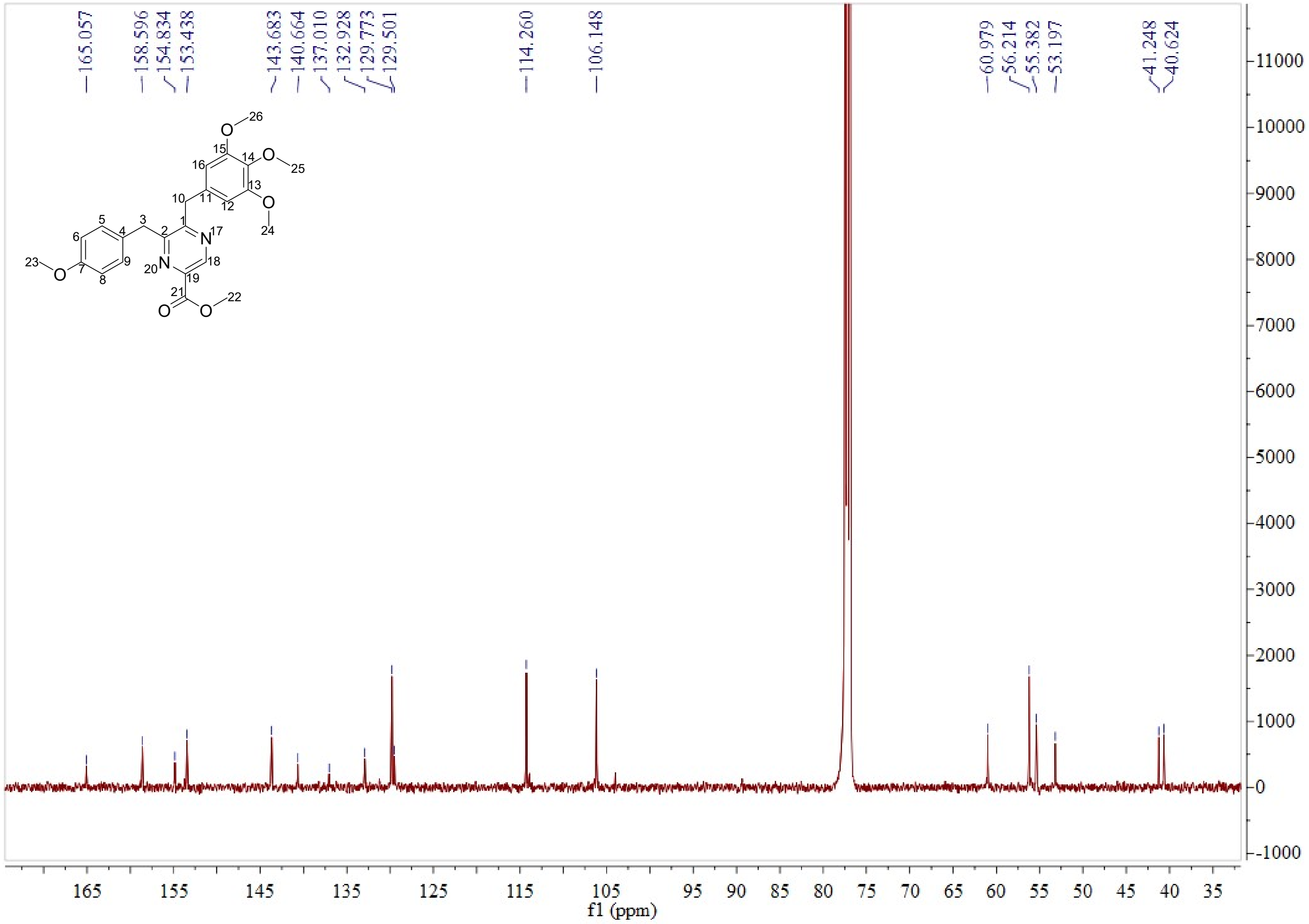

Figure S34. DEPT 135 spectrum of dichotomocej E (**3**) in $CDCl_3$ (125MHz)

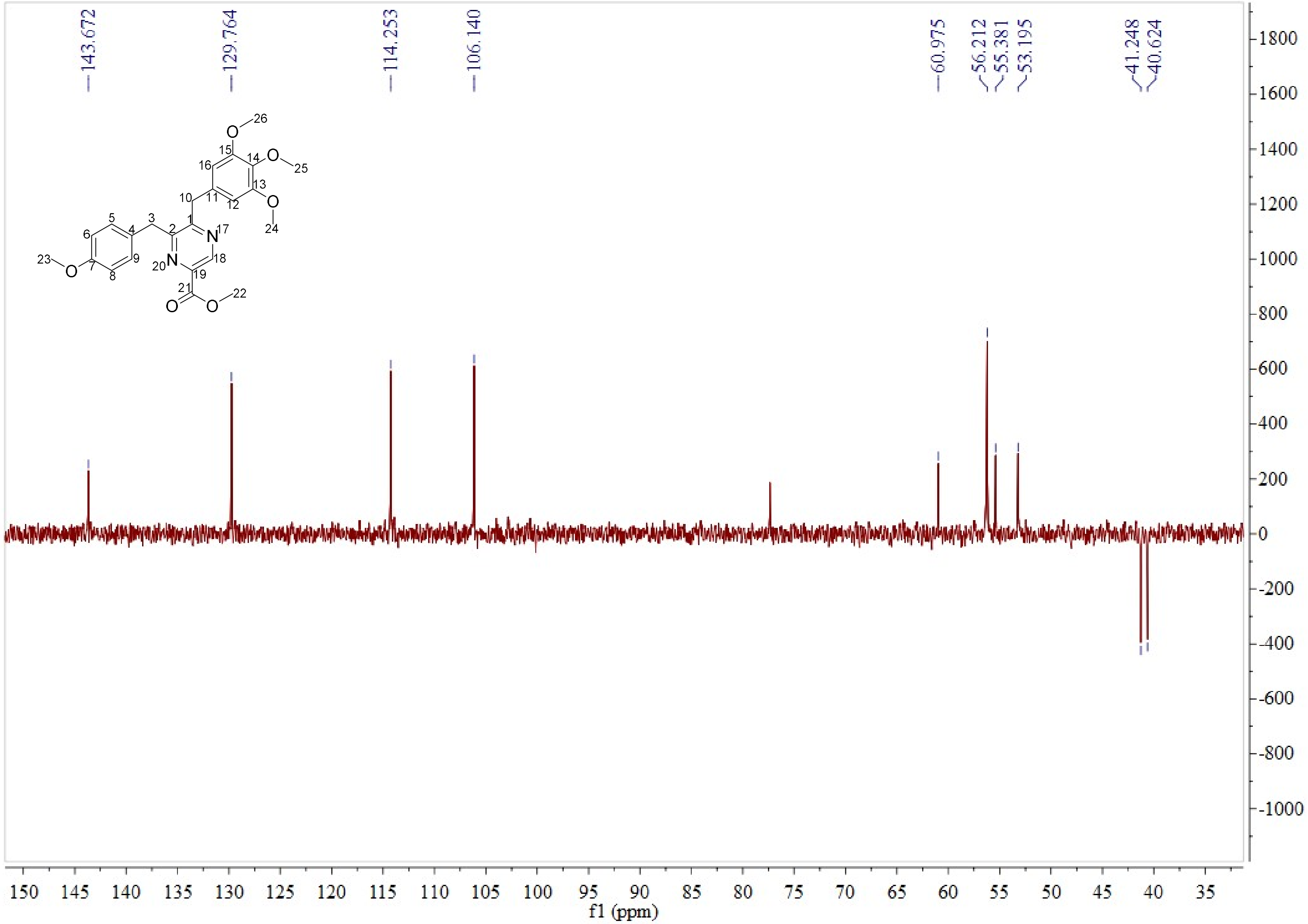

Figure S35. HMQC spectrum of dichotomocej E (**3**) in $CDCl_3$

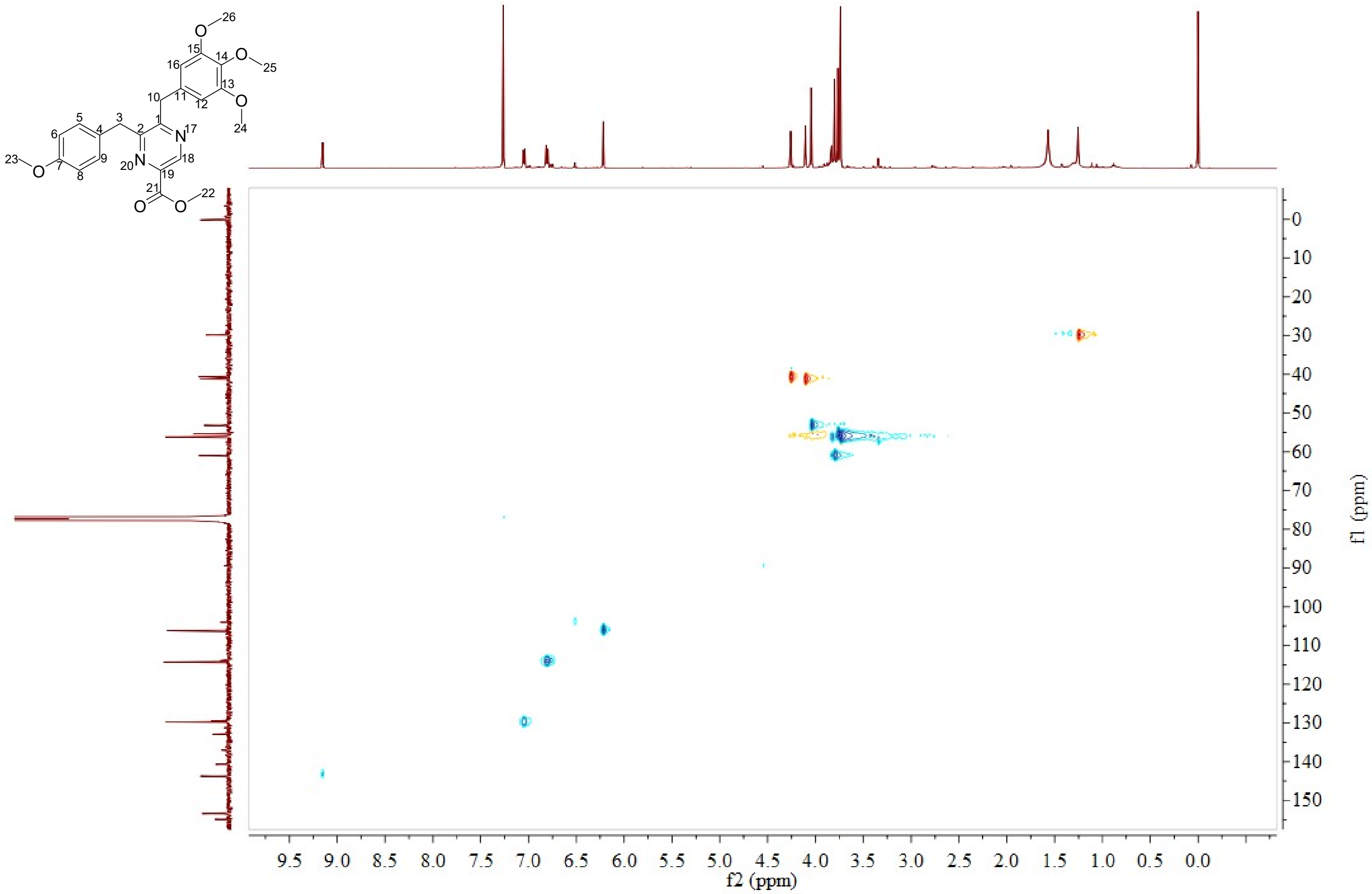

Figure S36. ^{1}H-^{1}H COSY spectrum of dichotomocej E (**3**) in $CDCl_3$

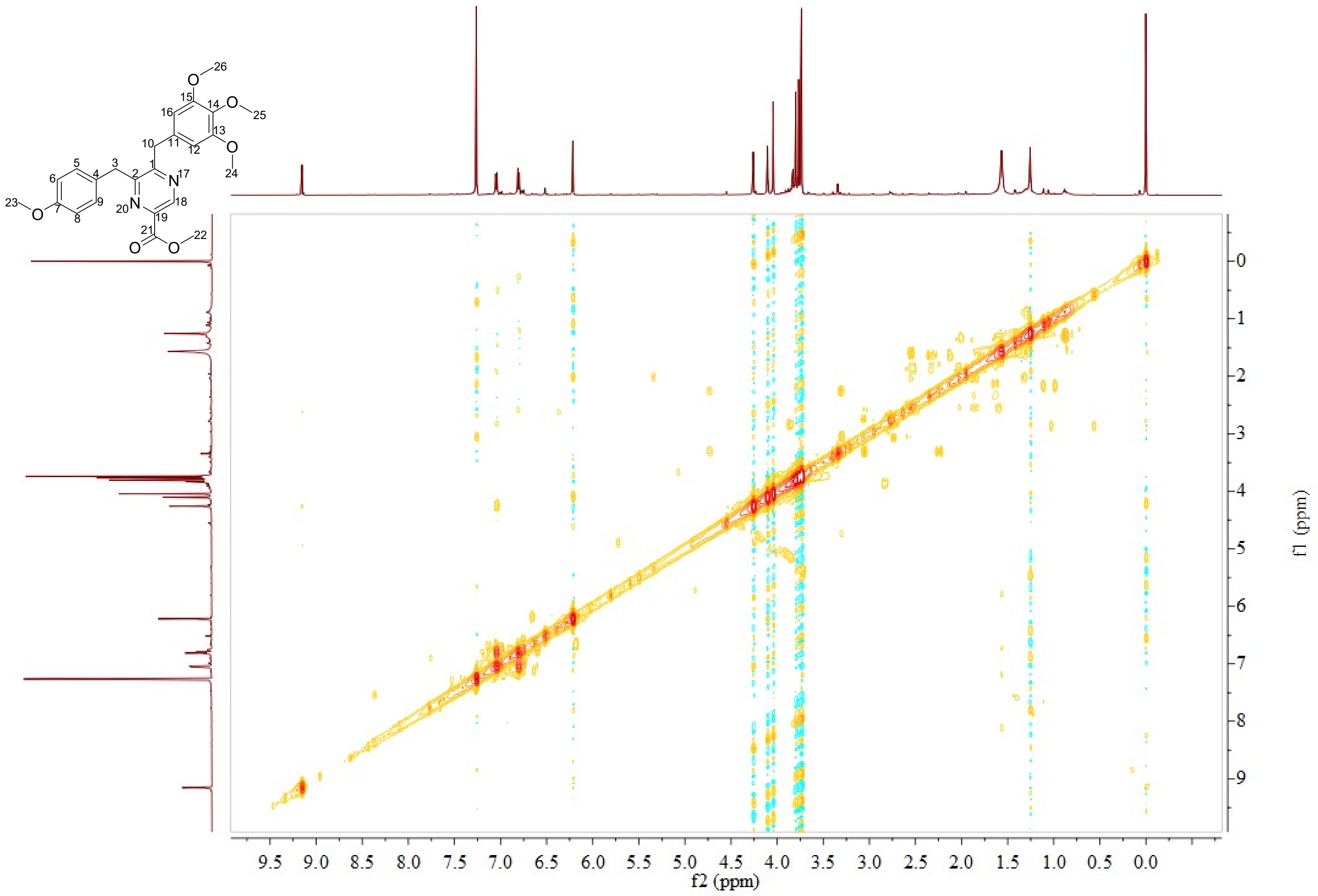

Figure S37. HMBC spectrum of dichotomocej E (**3**) in $CDCl_3$

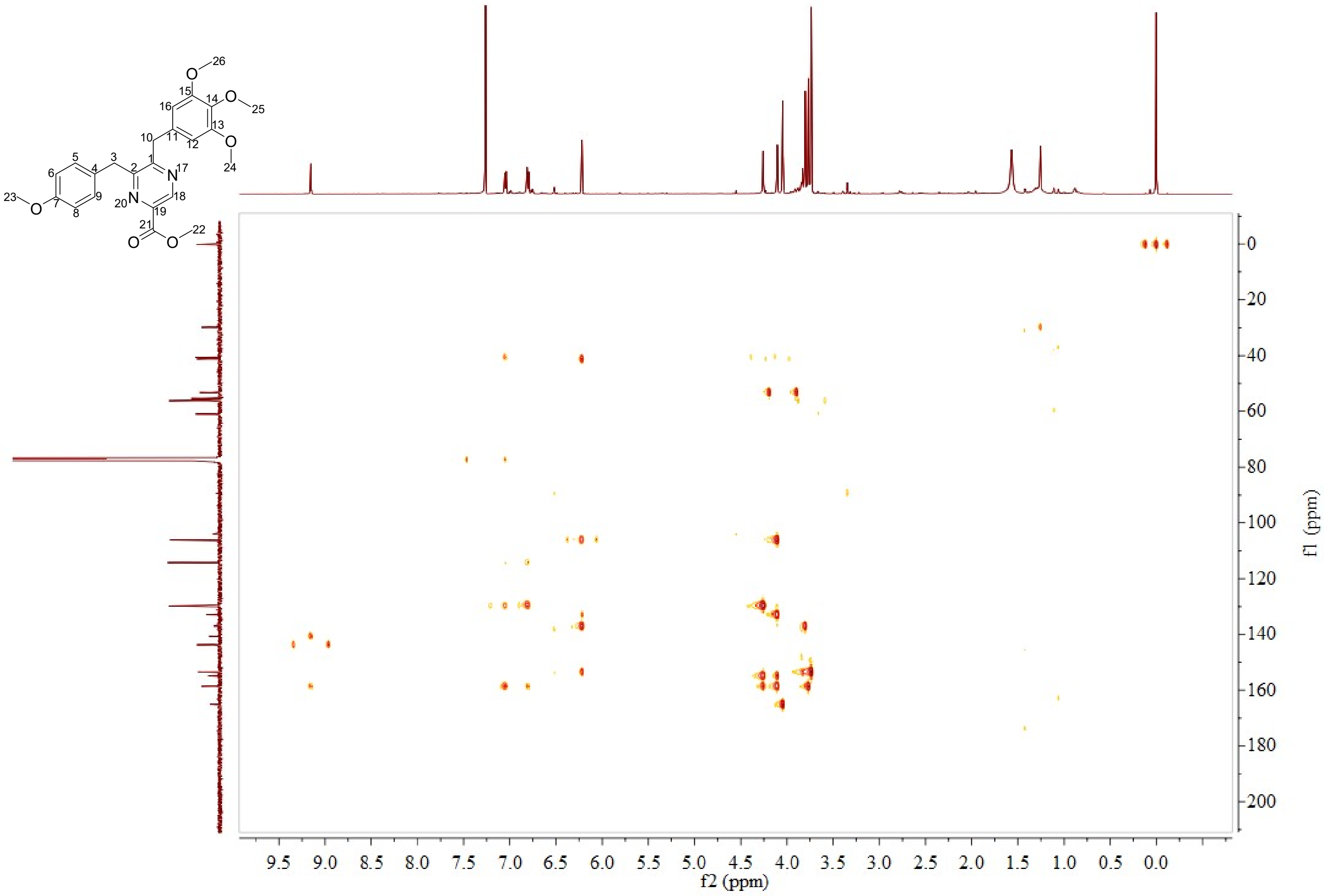

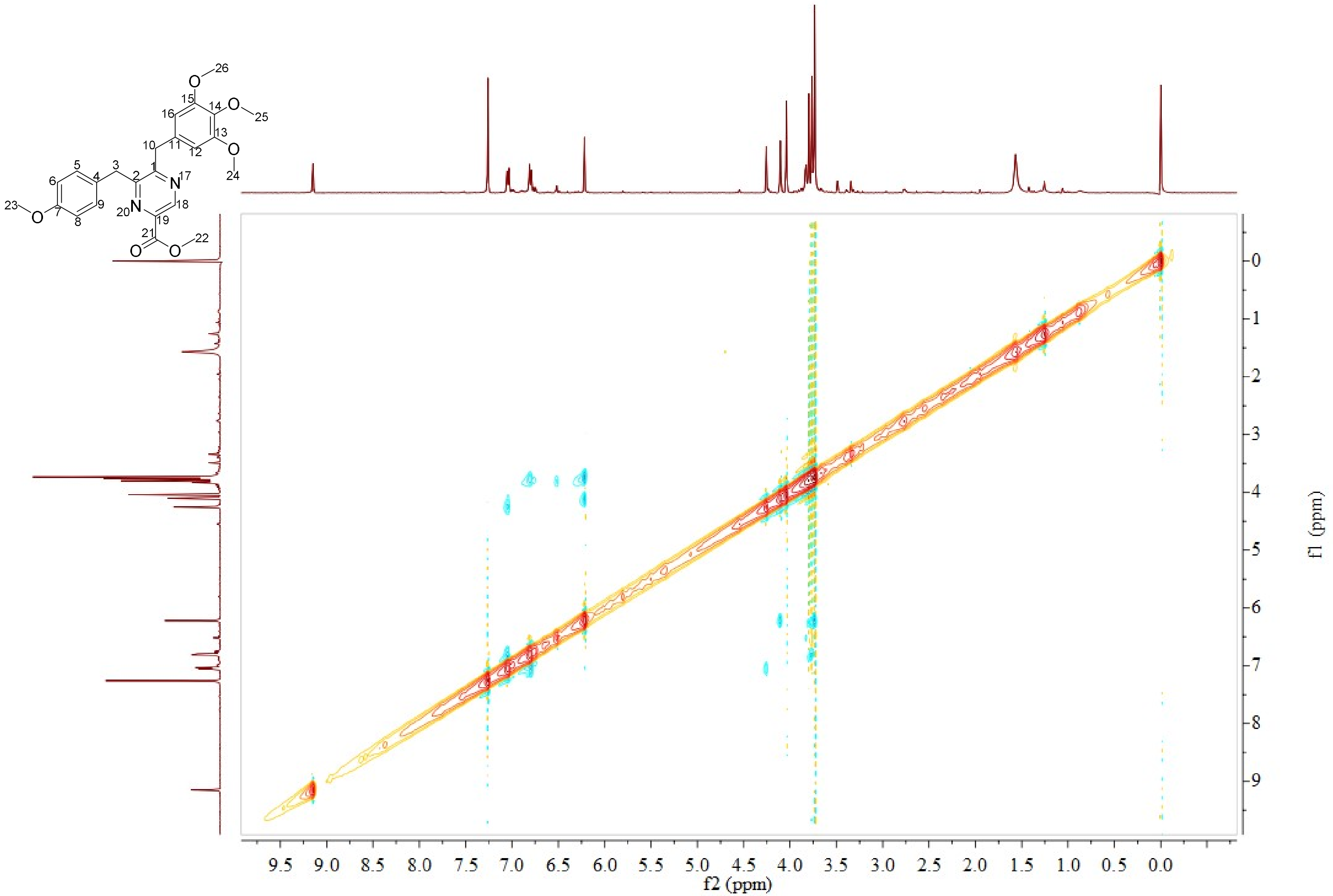

Figure S38. NOESY spectrum of dichotomocej E (**3**) in $CDCl_3$

Figure S39. HR-ESI-MS spectrum of dichotomocej F (**4**)

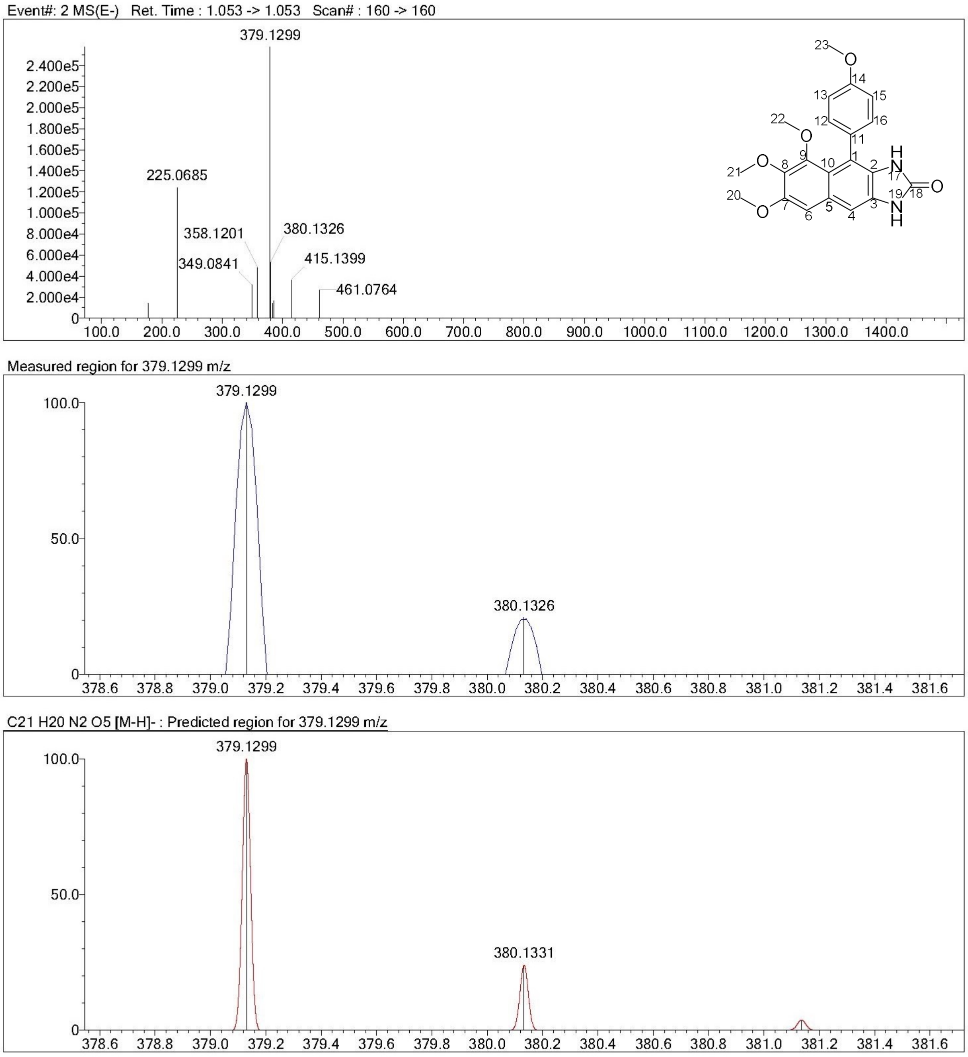

Rank	Score	Formula (M)	Ion	Meas. m/z	Pred. m/z	Df. (mDa)	Df. (ppm)	Iso	DBE
1	82.68	C21 H20 N2 O5	[M-H]-	379.1299	379.1299	-0.0	0.00	82.68	13.0

Figure S40. ^{1}H NMR spectrum of dichotomocej F (**4**) in $CDCl_3$ (400MHz)

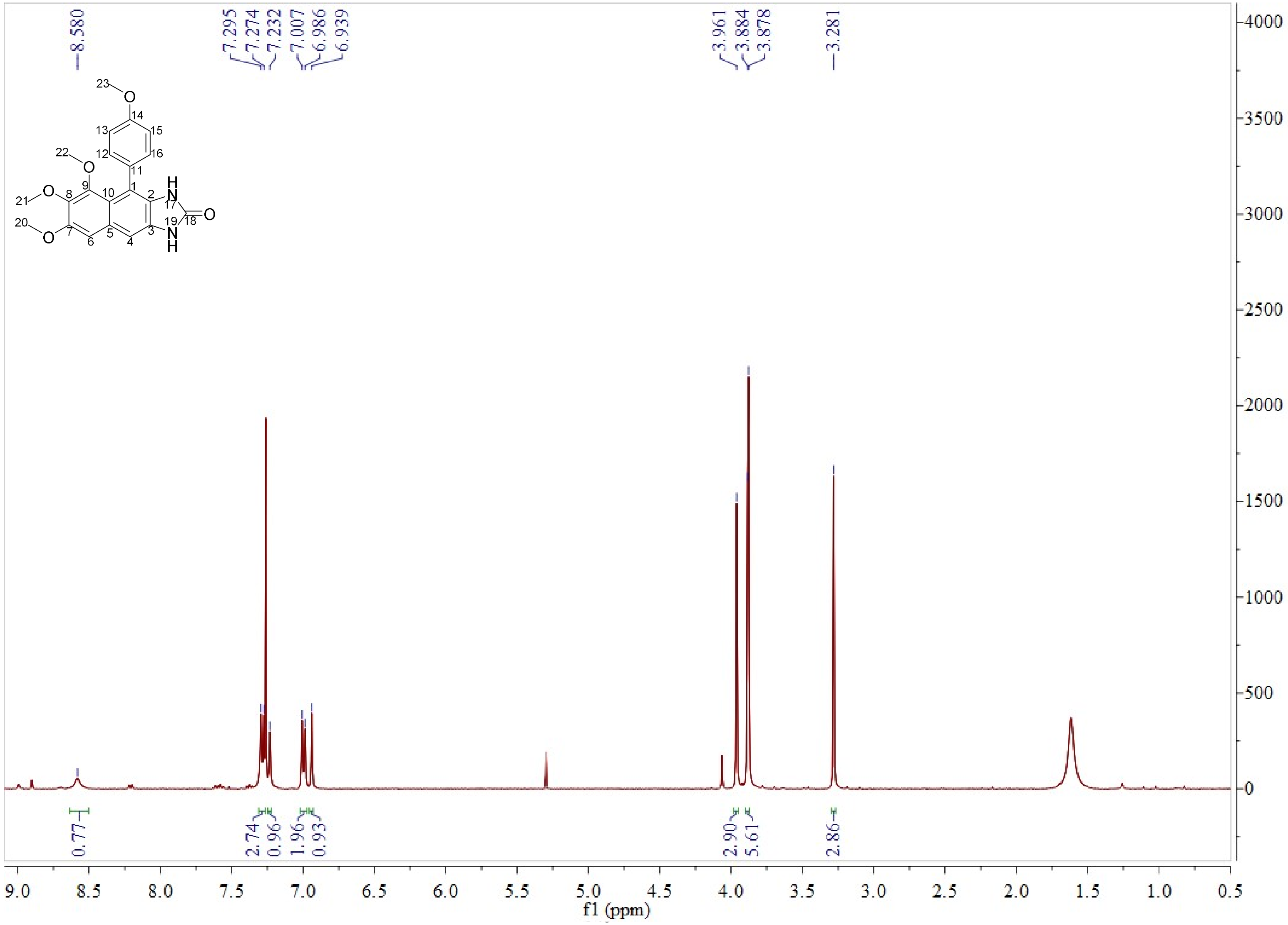

Figure S41. An expansion of ^{1}H NMR spectrum of dichotomocej F (**4**) in $CDCl_3$ (400MHz)

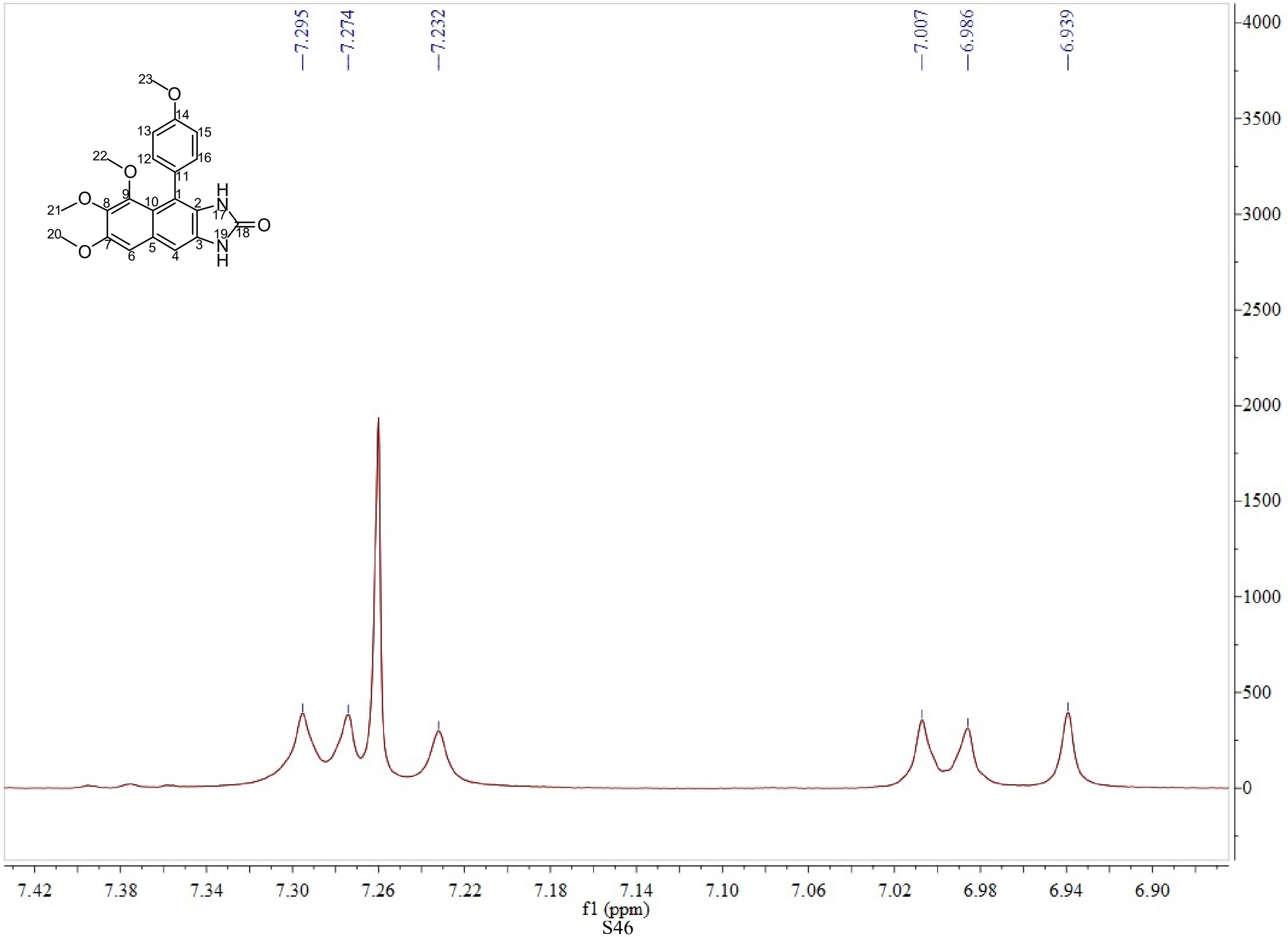

Figure S42. ^{13}C NMR spectrum of dichotomocej F (**4**) in $CDCl_3$ (100MHz)

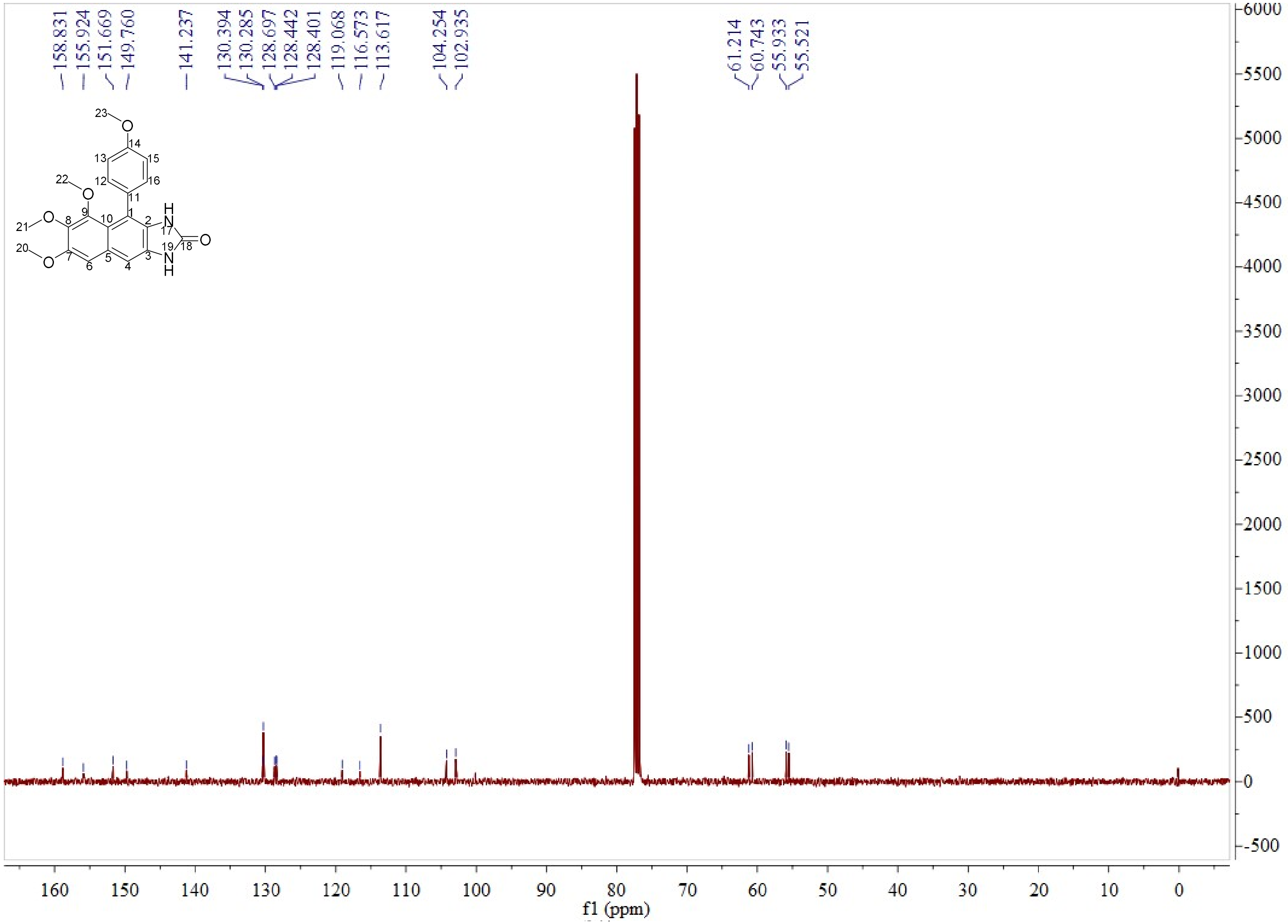

Figure S43. DEPT 135 spectrum of dichotomocej F (**4**) in $CDCl_3$ (100MHz)

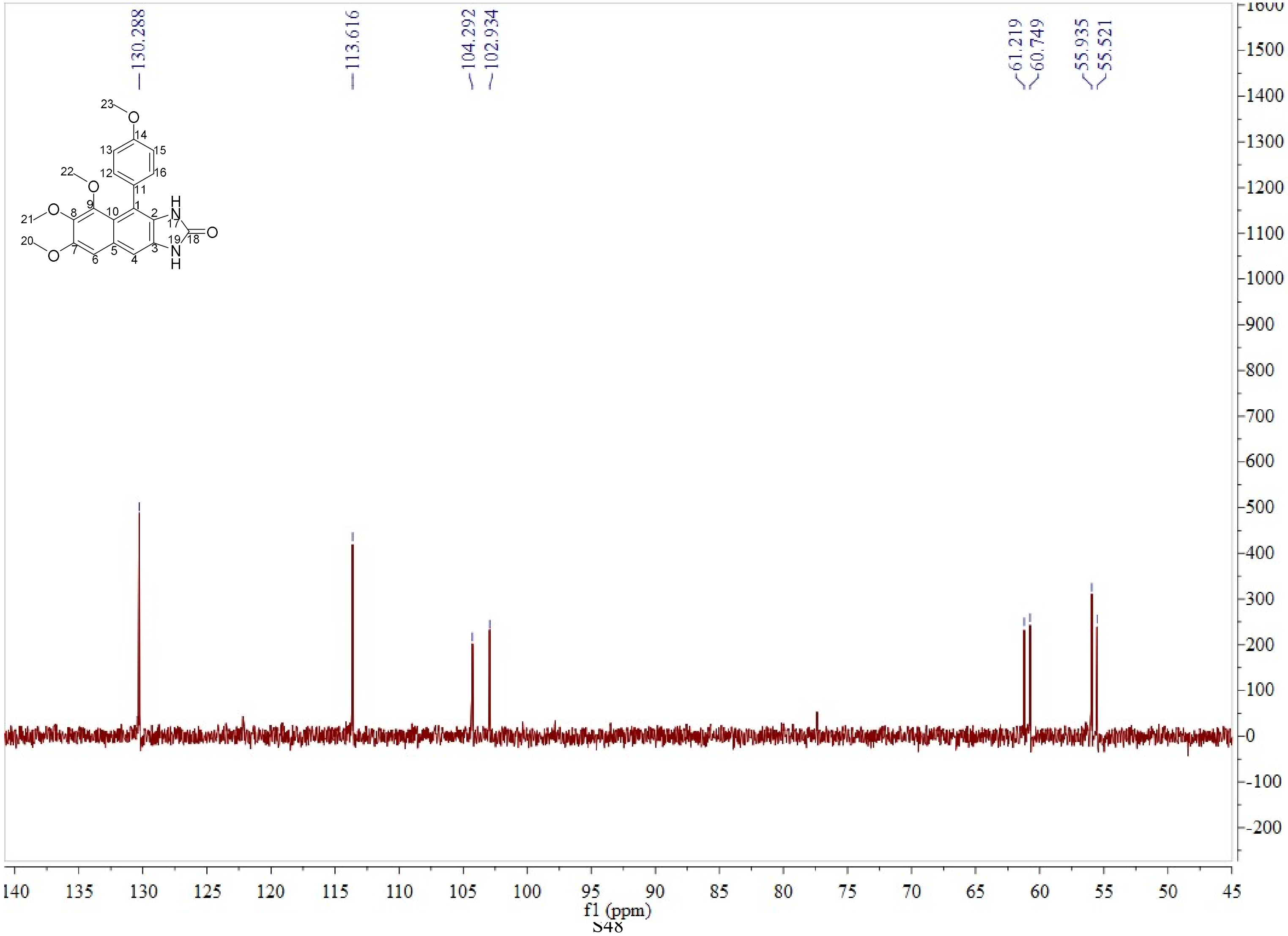

Figure S44. HSQC spectrum of dichotomocej F (**4**) in $CDCl_3$

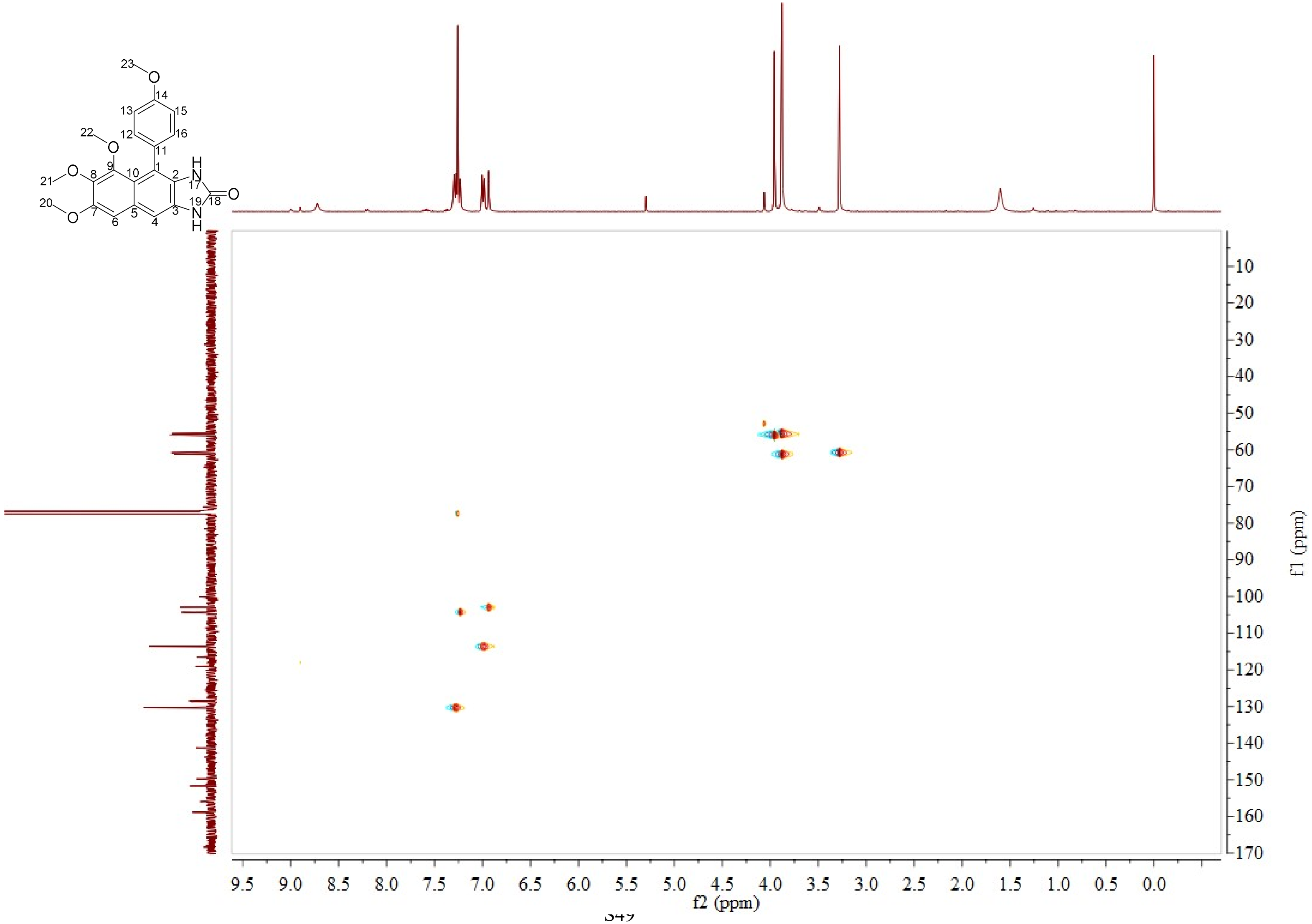

Figure S45. ^{1}H-^{1}H COSY spectrum of dichotomocej F (**4**) in $CDCl_3$

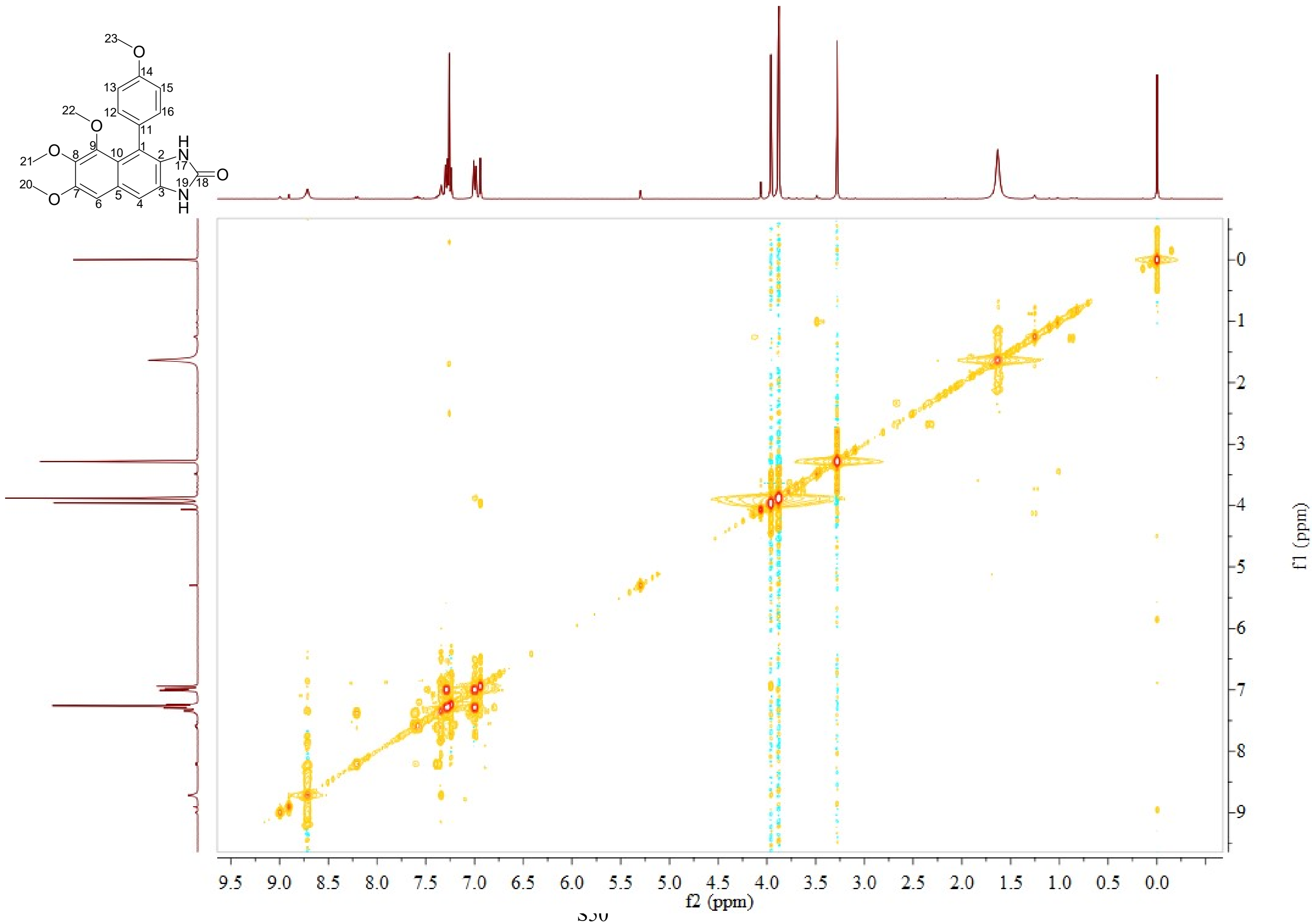

Figure S46. HMBC spectrum of dichotomocej F (**4**) in $CDCl_3$

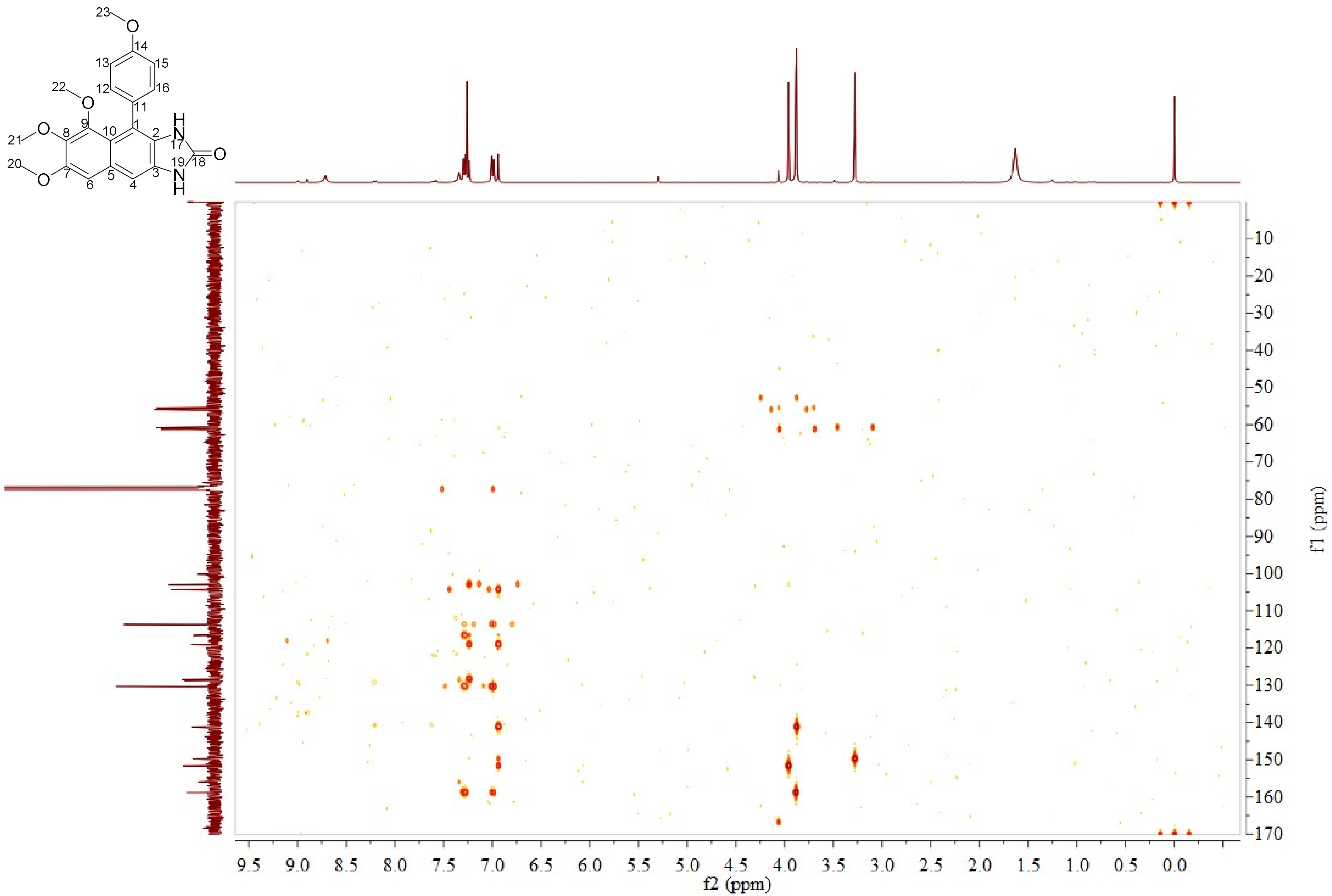

Figure S47. NOESY spectrum of dichotomocej F (**4**) in $CDCl_3$

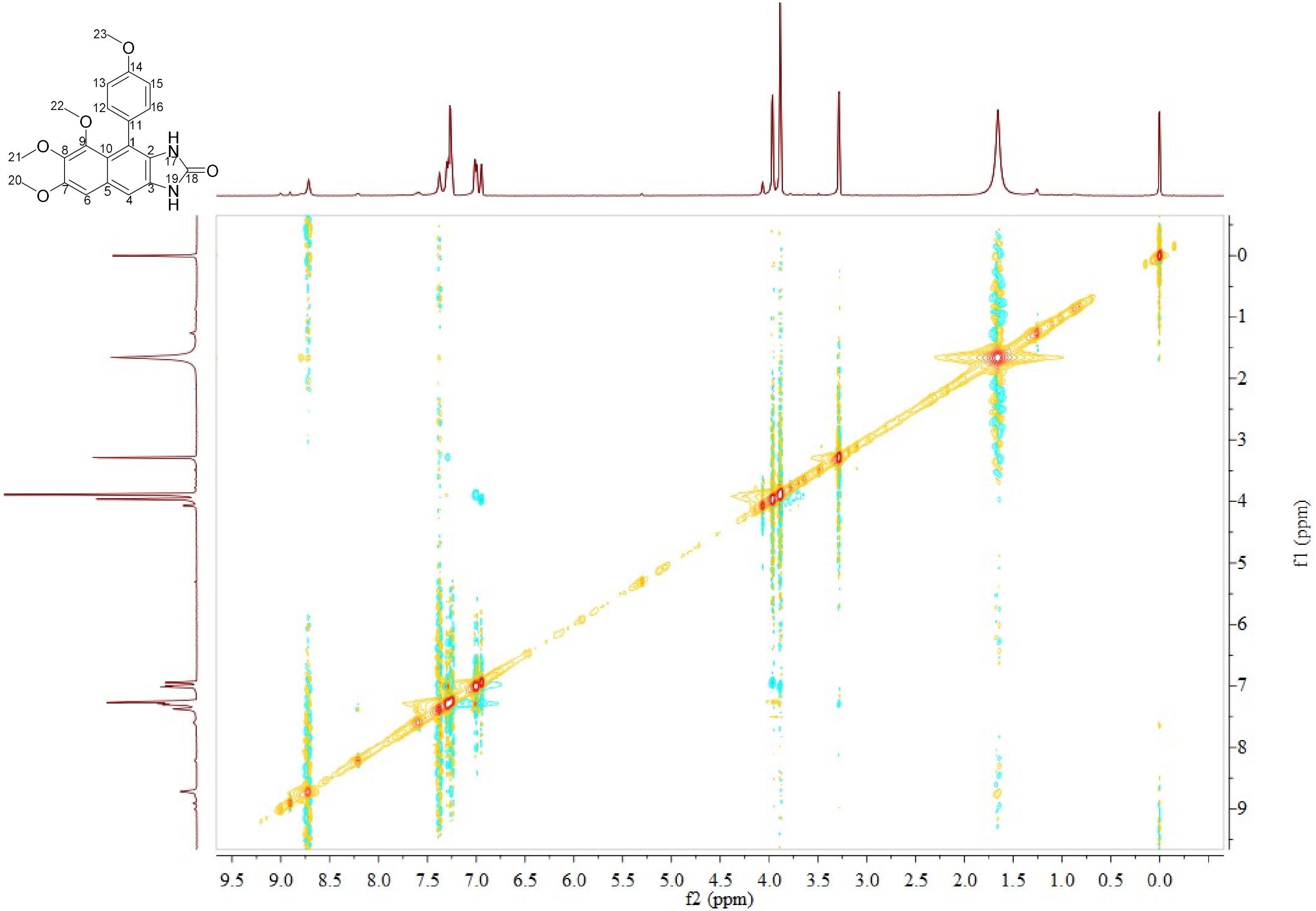

Figure S48. ^{1}H NMR spectrum of scequinadolines A (**5**) in $CDCl_3$

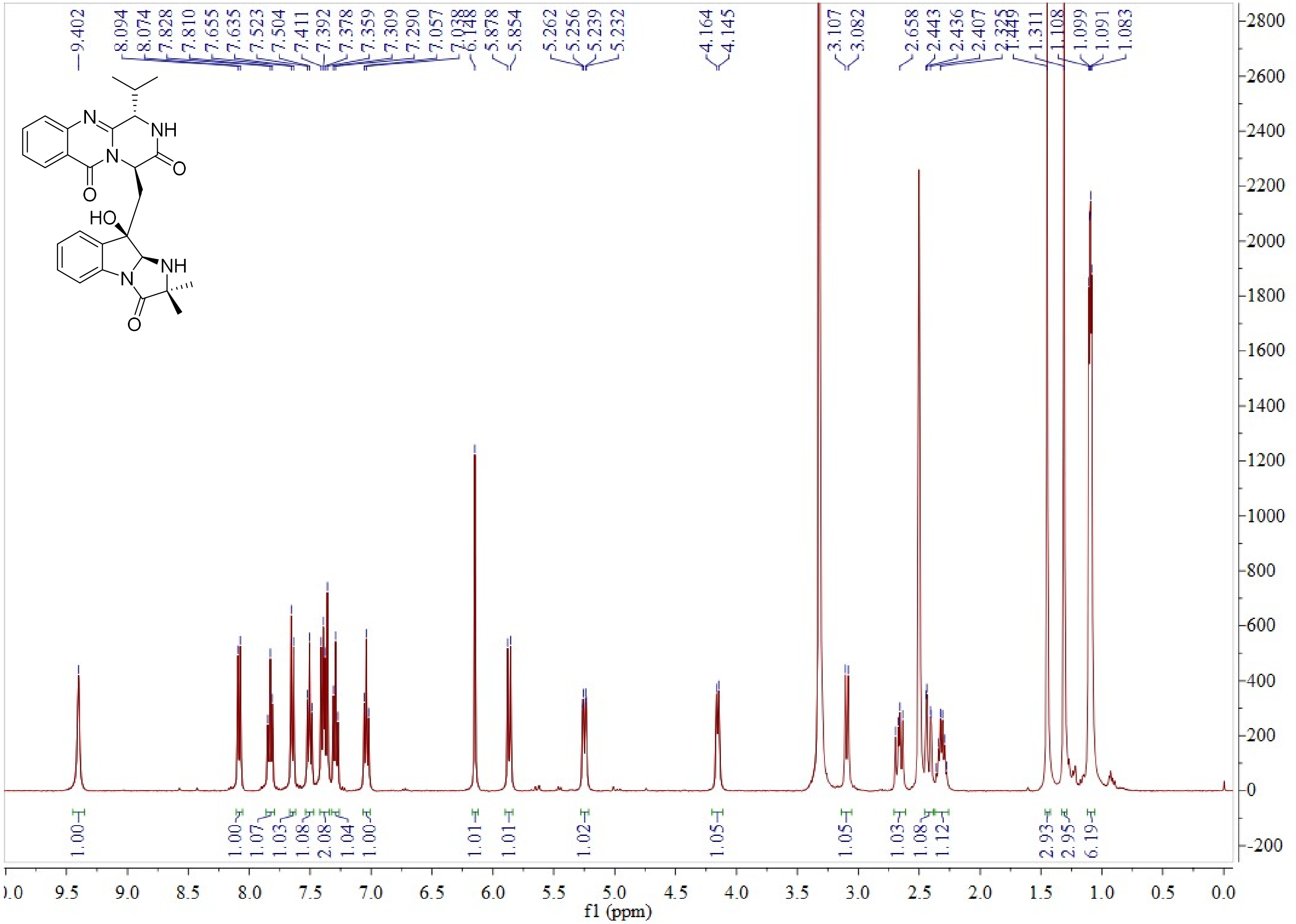

Figure S49. ^{13}C NMR spectrum of scequinadolines A (**5**) in $CDCl_3$

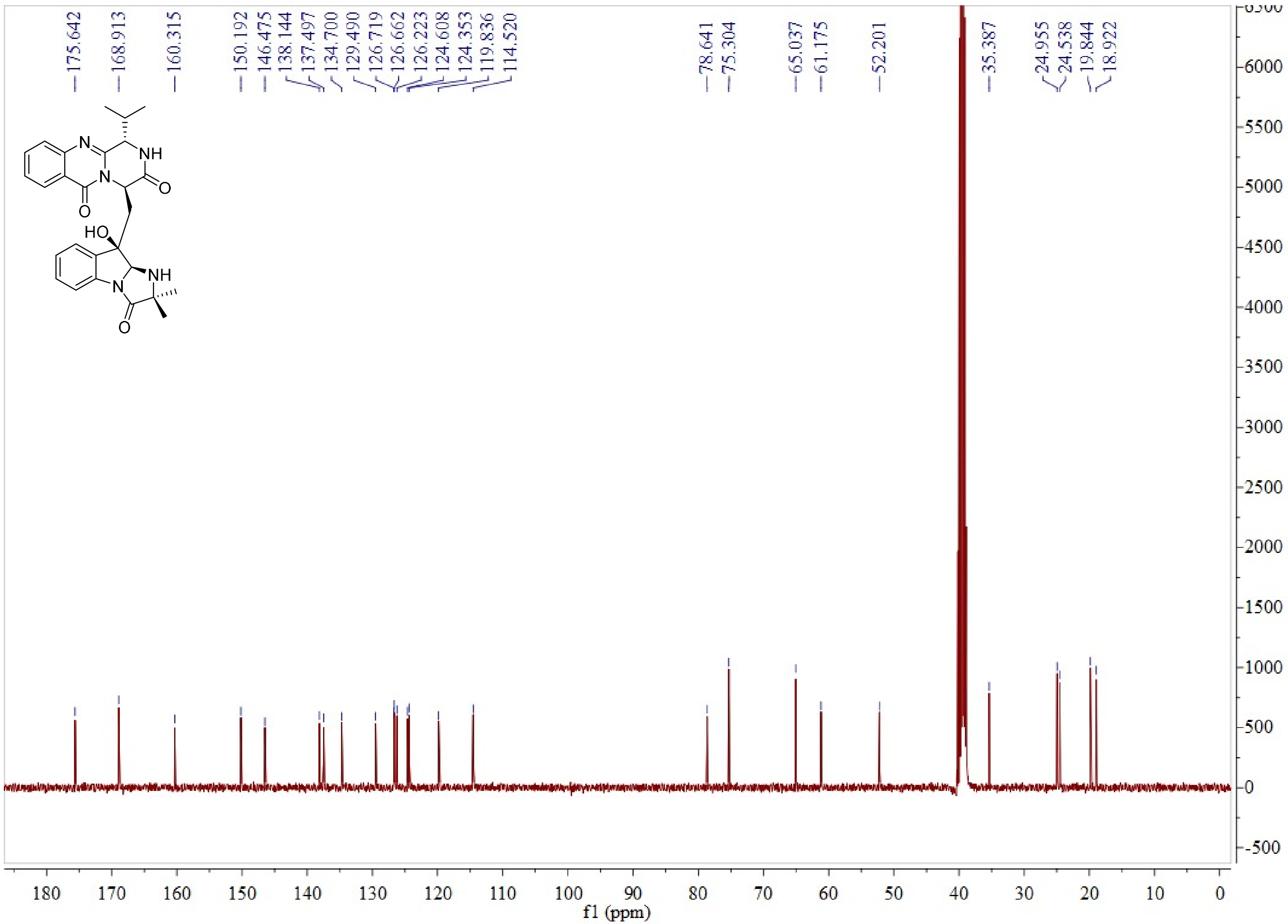

Figure S50. ^1H NMR spectrum of quinadoline A (**6**) in $CDCl_3$

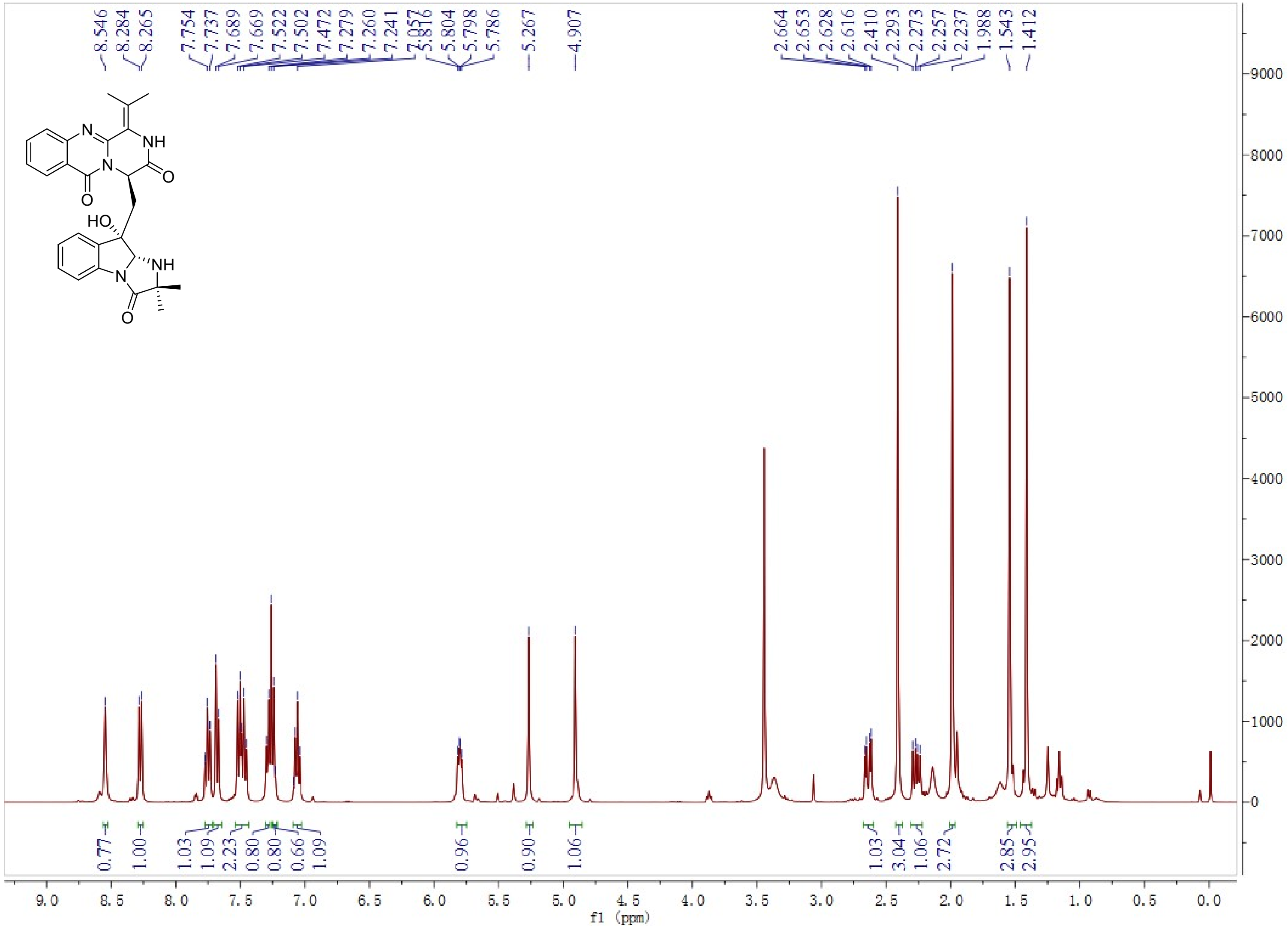

Figure S51. ^{13}C NMR spectrum of quinadoline A (**6**) in $CDCl_3$

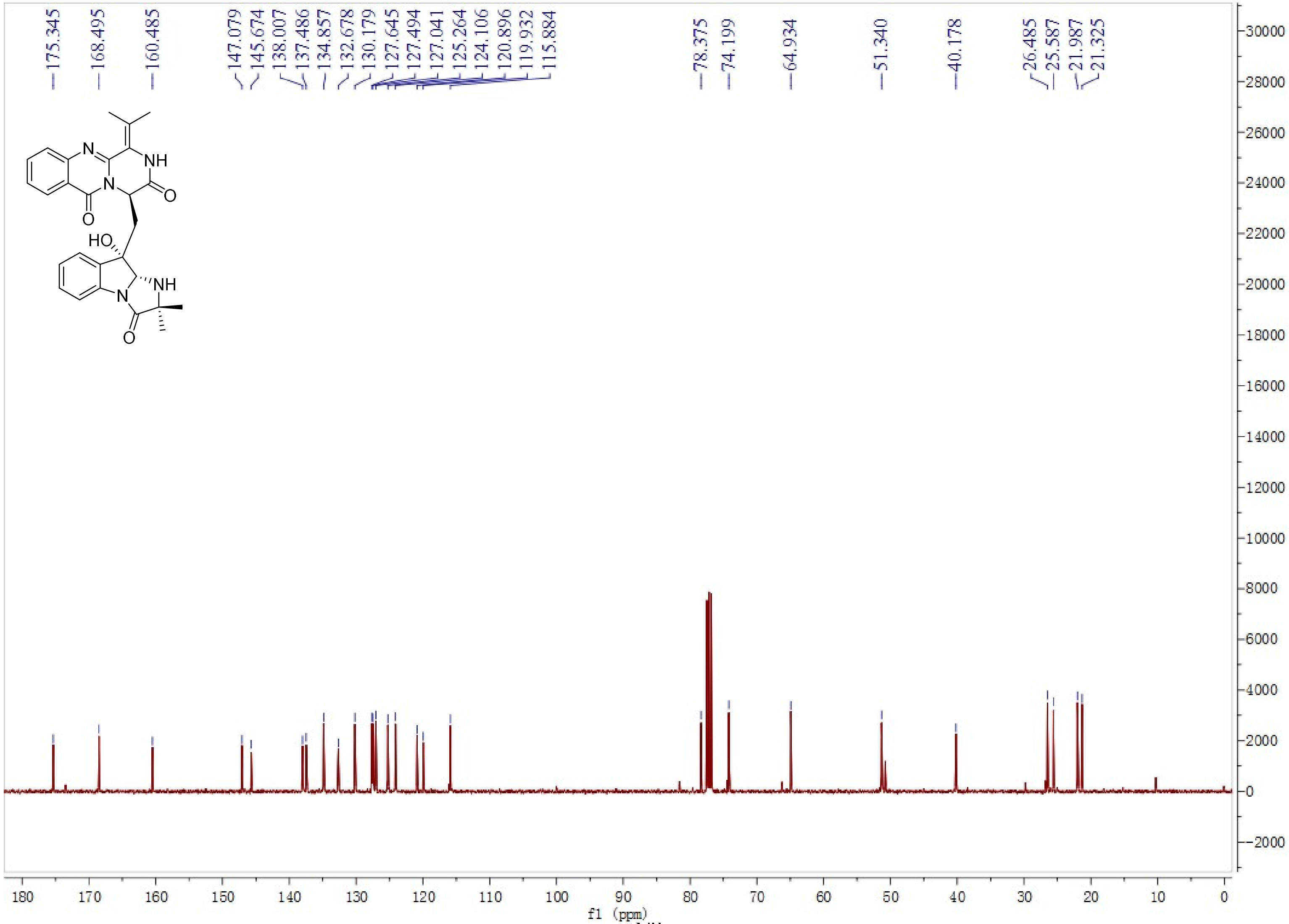

Figure S52. ^{1}H NMR spectrum of scequinadolines E (**7**) in CDCL3

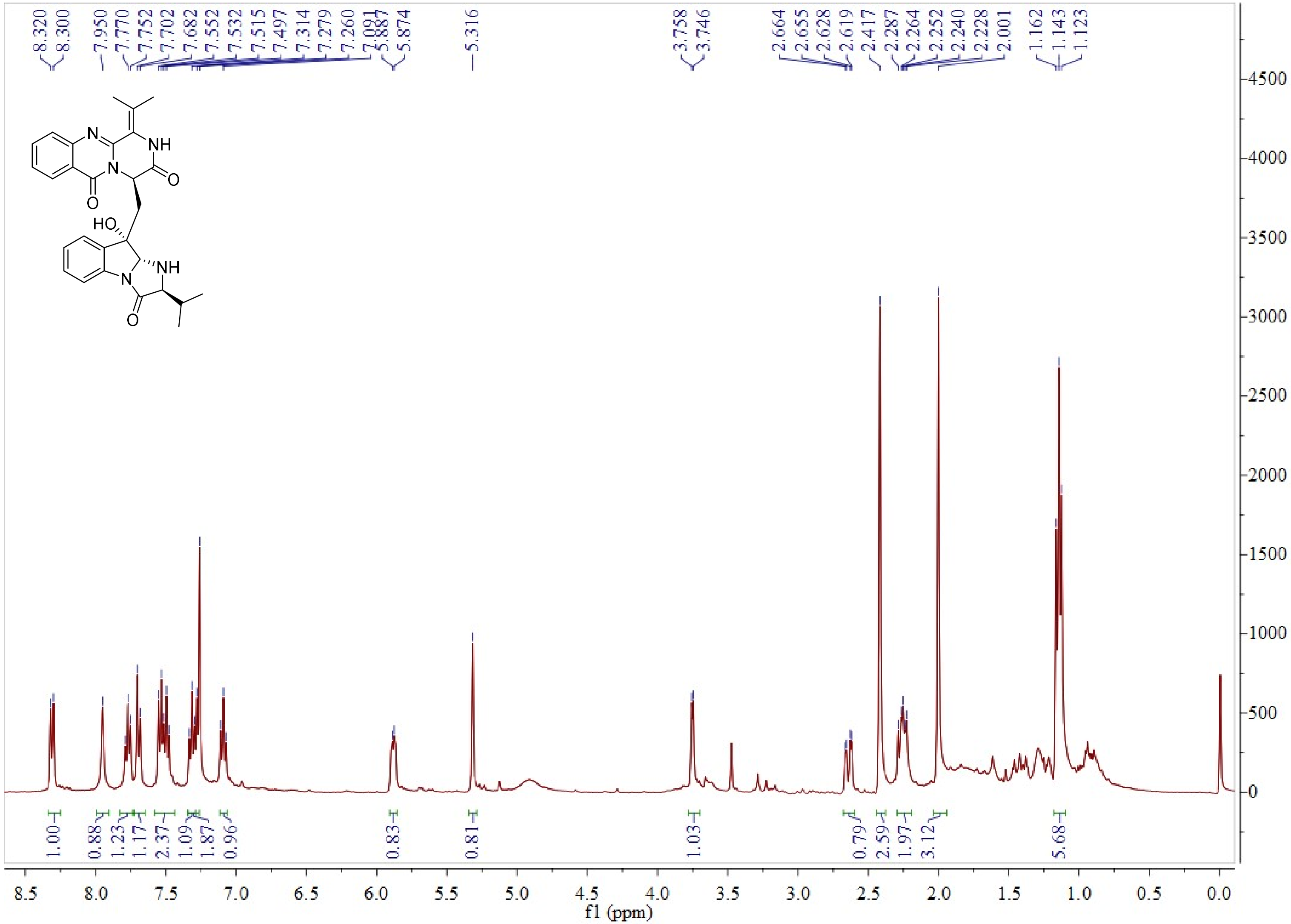

Figure S53. ^{13}C NMR spectrum of scequinadolines E (7) in CDCL$_3$

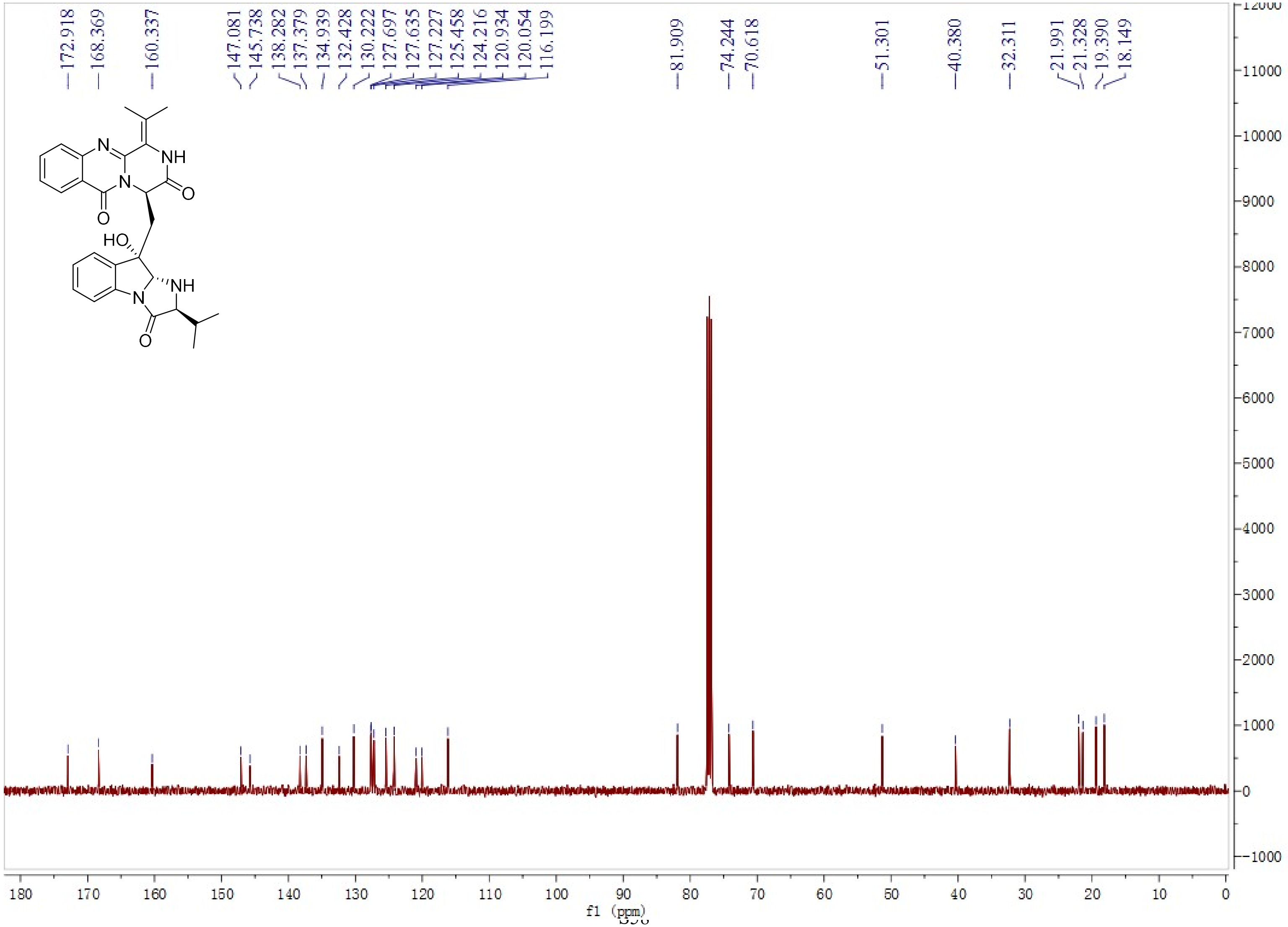

Supplement: Supplementary file 1 [file marinedrugs-16-00229-s001.pdf]
